# Effects of Halide Ions on the Carbamidocyclophane Biosynthesis in *Nostoc* sp. CAVN2

**DOI:** 10.3390/md14010021

**Published:** 2016-01-20

**Authors:** Michael Preisitsch, Stefan E. Heiden, Monika Beerbaum, Timo H. J. Niedermeyer, Marie Schneefeld, Jennifer Herrmann, Jana Kumpfmüller, Andrea Thürmer, Inga Neidhardt, Christoph Wiesner, Rolf Daniel, Rolf Müller, Franz-Christoph Bange, Peter Schmieder, Thomas Schweder, Sabine Mundt

**Affiliations:** 1 Institute of Pharmacy, Department of Pharmaceutical Biology, Ernst-Moritz-Arndt-University, Friedrich-Ludwig-Jahn-Straße 17, 17489 Greifswald, Germany; michael.preisitsch@uni-greifswald.de (M.P.); i.neidhardt@tu-braunschweig.de (I.N.); 2 Institute of Pharmacy, Department of Pharmaceutical Biotechnology, Ernst-Moritz-Arndt-University, Felix-Hausdorff-Straße 3, 17489 Greifswald, Germany; stefan.heiden@uni-greifswald.de (S.E.H.); jana.kumpfmueller@leibniz-hki.de (J.K.); schweder@uni-greifswald.de (T.S.); 3 Leibniz Institute for Molecular Pharmacology (FMP), Robert-Rössle-Straße 10, 13125 Berlin, Germany; beerbaum@fmp-berlin.de (M.B.); schmieder@fmp-berlin.de (P.S.); 4 Interfaculty Institute of Microbiology and Infection Medicine, Eberhard Karls University, Auf der Morgenstelle 28, 72076 Tübingen, Germany; timo.niedermeyer@uni-tuebingen.de; 5 German Centre for Infection Research (DZIF), Partner Site Tübingen (T.H.J.N.) and Partner Site Hannover-Braunschweig, Germany; schneefeld.marie@mh-hannover.de (M.S.); jennifer.herrmann@helmholtz-hzi.de (J.H.); rolf.mueller@helmholtz-hzi.de (R.M.); bange.franz@mh-hannover.de (F.-C.B.); 6 Institute for Medical Microbiology and Hospital Epidemiology, Hannover Medical School, Carl-Neuberg-Straße 1, 30625 Hannover, Germany; 7 Helmholtz Institute for Pharmaceutical Research Saarland, Helmholtz Centre for Infection Research, and Department of Pharmaceutical Biotechnology, Saarland University, Campus E8.1, 66123 Saarbrücken, Germany; 8 Leibniz Institute for Natural Product Research and Infection Biology, Hans Knöll Institute (HKI), Department of Biomolecular Chemistry, Beutenbergstraße 11a, 07745 Jena, Germany; 9 Institute of Microbiology and Genetics, Department of Genomic and Applied Microbiology and Göttingen Genomics Laboratory, Georg-August University, Grisebachstraße 8, 37077 Göttingen, Germany; athuerm@gwdg.de (A.T.); rdaniel@gwdg.de (R.D.); 10 Institute of Technology, Department of Pharmacology, Toxicology and Clinical Pharmacy, Technical University of Braunschweig, Mendelssohnstraße 1, 38106 Braunschweig, Germany; 11 Sealife PHARMA GmbH, Technopark 1/Obj.C/EG, 3430 Tulln, Austria; wiesner@sealifepharma.com

**Keywords:** *Nostoc* sp., carbamidocyclophane, [7.7]paracyclophane, halogen, bromo-analogue, biosynthesis, gene cluster, bioactivity, MRSA, *Mycobacterium*

## Abstract

In this study, the influence of halide ions on [7.7]paracyclophane biosynthesis in the cyanobacterium *Nostoc* sp. CAVN2 was investigated. In contrast to KI and KF, supplementation of the culture medium with KCl or KBr resulted not only in an increase of growth but also in an up-regulation of carbamidocyclophane production. LC-MS analysis indicated the presence of chlorinated, brominated, but also non-halogenated derivatives. In addition to 22 known cylindrocyclophanes and carbamidocyclophanes, 27 putative congeners have been detected. Nine compounds, carbamidocyclophanes M−U, were isolated, and their structural elucidation by 1D and 2D NMR experiments in combination with HRMS and ECD analysis revealed that they are brominated analogues of chlorinated carbamidocyclophanes. Quantification of the carbamidocyclophanes showed that chloride is the preferably utilized halide, but incorporation is reduced in the presence of bromide. Evaluation of the antibacterial activity of 30 [7.7]paracyclophanes and related derivatives against selected pathogenic Gram-positive and Gram-negative bacteria exhibited remarkable effects especially against methicillin- and vancomycin-resistant staphylococci and *Mycobacterium tuberculosis*. For deeper insights into the mechanisms of biosynthesis, the carbamidocyclophane biosynthetic gene cluster in *Nostoc* sp. CAVN2 was studied. The gene putatively coding for the carbamoyltransferase has been identified. Based on bioinformatic analyses, a possible biosynthetic assembly is discussed.

## 1. Introduction

Cyanobacteria have proven to be a prolific source of structurally highly diverse and biologically active secondary metabolites. Especially from marine species a huge array of bioactive lipopeptides has been isolated. These compounds are mostly derived from the combination of polyketide synthase (PKS) and non-ribosomal peptide synthetase (NRPS) based biosynthetic pathways [1,2,3].

[7.7]Paracyclophanes have been isolated exclusively from soil and freshwater cyanobacterial species belonging to the genera *Cylindrospermum* and *Nostoc*. Since the initial report of cylindrocyclophane A from *Cylindrospermum licheniforme* Kützing ATCC 29204 and nostocyclophane D from *Nostoc linckia* (Roth) Bornet UTEX B 1932 by Moore *et al.* in 1990 [4], 32 other naturally occurring [7.7]paracyclophanes have been published. Generally, these compounds possess a remarkable symmetric hydrocarbon macrocycle consisting of two resorcinols linked by two aliphatic chains. This core structure is decorated with a variety of substituents, such as methyl, hydroxy, acetoxy or carbamate groups. Furthermore, glycosylations of the phenolic moieties and halogenation to a varying degree have been reported. Regardless of the individual substitution patterns, the derivatives exhibit cytotoxicity against various cancer cell lines in the low micromolar range but also against non-tumorigenic cells [5,6,7,8,9,10,11].

The unique carbon backbone of the [7.7]paracyclophanes has attracted the interest of organic chemists, and several total syntheses of cylindrocyclophanes A and F have been developed [12,13]. Subsequent improvement of their synthetic routes led to both a reduction of required steps and a significant increase in yield [14,15]. The major (bio)synthetic theme in [7.7]paracyclophane formation—a head-to-tail cyclodimerization of monomeric alkylresorcinol intermediates towards the final *C*_2_-symmetric macrocyclic skeleton—was already proposed from feeding experiments with isotopically labeled sodium acetate by Bobzin and Moore in 1993, suggesting the [7.7]paracyclophane core to be of polyketide origin [16]. The recently published discovery of the cylindrocyclophane gene cluster in *C. licheniforme* UTEX ‘B 2014’ (also designated as ATCC 29412) by Nakamura *et al.* corroborated a monomeric, ‘unusual’ biosynthetic logic. Via a chemically guided genome mining approach and feeding studies, the authors showed that biosynthesis of a putative monomeric intermediate is accomplished in particular by PKS-mediated elongation and aromatization steps of decanoic acid, which is most likely the initial precursor for cylindrocyclophane biosynthesis [17,18,19]. However, the exact enzymatic mechanisms behind the accomplishment of intermolecular dimerization as well as the halogenation events by the biosynthetic machinery are still not completely resolved. According to recent research results, the hypothetical protein CylC of the cylindrocyclophane gene cluster and related homologues seem to present a hitherto unknown type of halogenase that facilitates halogenation of alkyl chains by an unusual C-H bond activation. CylC is also discussed to be involved in C-C bond activation of cylindrocyclophane biosynthesis [20,21].

Chlipala *et al.* isolated the first tetrabrominated [7.7]paracyclophane, cylindrocyclophane A_B4_, from the terrestrial cyanobacterium *Nostoc* sp. UIC 10022A cultured in KBr-enriched medium, indicating a low substrate specificity of the putative halogenase involved in the cylindrocyclophane biosynthesis of that strain [8]. However, studies on the ability to incorporate other halogen atoms than chlorine and bromine or to identify a homologous biosynthetic gene cluster from cyanobacterial strains known to biosynthesize halogenated [7.7]paracyclophanes have not been published so far.

In previous work, carbamidocyclophane derivatives, differing from other congeners by the presence of one or two carbamate moieties within the molecule, have been reported to exhibit pronounced bioactivity against Gram-positive bacteria such as methicillin-resistant *Staphylococcus aureus* (MRSA), *Streptococcus pneumoniae*, and *Mycobacterium tuberculosis* [6,7]. Based on these promising bioactivities, we decided to investigate the biosynthesis of carbamidocyclophanes in *Nostoc* sp. CAVN2 both on a molecular and metabolic level with the emphasis on the generation of further halogenated metabolites. Here, we describe cultivation approaches to evaluate the effect of halide salts on both the growth and the carbamidocyclophane biosynthesis. These studies led to the detection, isolation and structure elucidation of nine new brominated analogues. In addition, a panel of 30 [7.7]paracyclophanes and related congeners was tested against 16 biological targets, such as drug-susceptible and drug-resistant Gram-positive as well as Gram-negative bacteria. Moreover, first results of our ongoing effort to elucidate the biosynthetic assembly of carbamidocyclophanes are presented, and the putative biosynthesis gene cluster of *Nostoc* sp. CAVN2 is compared in detail with the cylindrocyclophane gene clusters of the *Cylindrospermum strains* UTEX ‘B 2014’ and PCC 7417.

## 2. Results and Discussion

### 2.1. Testing of Halide Anion Incorporation

In the field of natural product drug discovery from bacteria, the substitution of chloride by supplementation of other halide anions to the culture medium is a common strategy attempting to raise the structural diversity of halogenated compounds. This mostly led to the biosynthesis of bromo-derivatives, e.g., in case of chlortetracycline [22], chloramphenicol [23,24], pyrrolnitrin [25,26], monamycin [27], pyrrolomycin [28], streptopyrrole [29], and balhimycin [30].

Only very few fluorine or iodine containing compounds have been isolated based on that approach, e.g., iodinated calicheamicins [31,32]. Often, chlorinated bacterial compounds show higher biological activity than their non-halogenated or otherwise halogenated derivatives [25,26,29,33]. Notable exceptions are reported for bromobalhimycin [30], brominated pyrrolomycins [28], and some bromine-containing napyradiomycins [34,35] as well as the lantibiotic NAI-108 [36], and the synthetic fluoro-derivatives of marinopyrrole A [33]. In addition, the aforementioned cylindrocyclophane A_B4_ was the most active derivative in the 20S proteasome assay [8].

In an initial screening, we investigated the effect of the individual halides on both the biosynthesis of halogenated [7.7]paracyclophanes in *Nostoc* sp. CAVN2 and the cyanobacterial growth. In contrast to most of the above-mentioned studies, stock cultures, routinely grown in chlorine-containing BG-11 (≈ 0.5 mM Clˉ) [37] or modified WC (MBL) (≈ 0.5 mM Clˉ) [7] medium, were transferred to low-level halogen-containing Z½ medium (<0.1 µM halide ions, Table S1) and were subjected to multiple cultivation dilution until halogenated [7.7]paracyclophanes were only detectable in traces by HPLC-UV-MS analysis. Subsequently, potassium salts of chlorine, bromine, iodine, and fluorine were added to the culture broth to give a concentration of either 0.001, 0.01, 0.1, or 1.0%. Cultivation was performed at 25 °C, *i.e.*, at a temperature that leads to an optimal ratio between biomass production and carbamidocyclophane content [37]. Generally, lower halide salt concentrations did not influence cyanobacterial growth over 20, 25, and 30 days of cultivation. Supplementation with KI and KF in the two higher concentrations resulted in an up to twofold decrease of biomass production, but KBr and KCl in that concentration range seem to improve growth slightly (Table S2).

**Figure 1:**
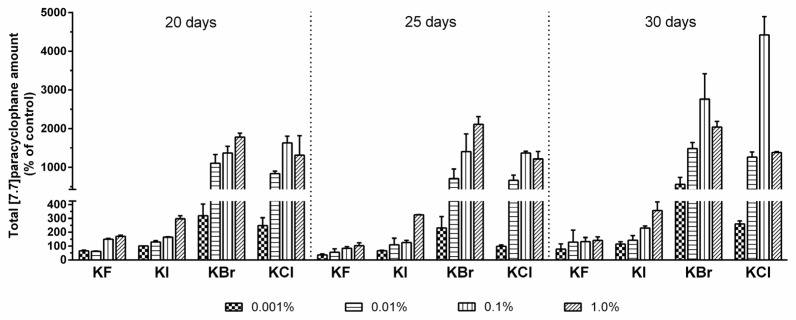
Total [7.7]paracyclophane amounts of *Nostoc* sp. CAVN2 cultures grown in the presence of different potassium halide salts at varying concentration levels for 20‒30 days. Values shown are expressed as the mean ± standard error of the mean (SEM), *n* = 2.

However, the total average [7.7]paracyclophane amounts generally correlated positively with increasing halogen salt concentrations and cultivation time. Especially by addition of chloride and bromide, highest compound production was observed in cultures supplemented with 0.1% of these halide salts after 30 days, which means an up to 44-fold and 28-fold, respectively, increase of the [7.7]paracyclophane quantity compared to the unspiked control samples (Figure 1).

**Figure 2:**
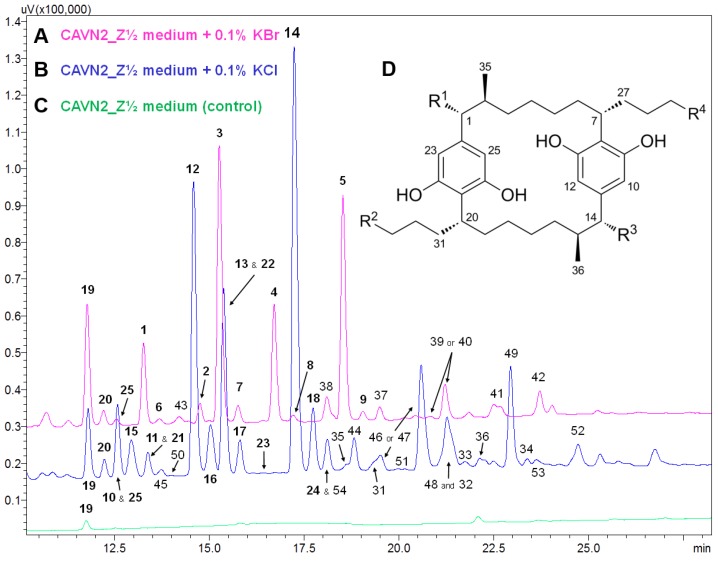
Overlay of HPLC-UV chromatograms (λ = 226 nm) of extracts from *Nostoc* sp. CAVN2 cultures grown in the presence of 0.1% KBr (**A**) or 0.1% KCl (**B**) for 30 days *versus* the control (**C**). (**D**) Carbamidocyclophane and cylindrocyclophane core structure. Specific substitution patterns of identified compounds and structural proposals of non-elucidated derivatives are listed in Table 1.

HPLC-UV-MS analysis of samples cultured in the presence of chloride or bromide indicated a variety of known [7.7]paracyclophanes and yet unknown compounds. An overlay of the HPLC-UV chromatograms from samples cultured with 0.1% KBr/KCl for 30 days is shown in Figure 2. Compound identifications as well as structural proposals of selected peaks are given in Table 1. In particular, chloride supplementation triggered the biosynthesis of carbamidocyclophanes and cylindrocyclophanes that have already been isolated in our previous study [7]. Samples cultured in the presence of bromide indicated a range of brominated carbamidocyclophanes with structures analogous to those obtained with chloride, the respective chlorine atoms substituted by bromine. In contrast, examination of the analytical data of the fluorine and iodine feeding studies did not reveal any fluorinated or iodinated congeners. Total [7.7]paracyclophane increase of these samples was especially due to non-halogenated derivatives, for example carbamidocyclophanes E (**19**) and H (**20**).

**Table 1:** Identification of known [7.7]paracyclophanes and structural proposals of unknown congeners according to selected peaks of Figure 2. Compound assignment based on accurate mass and HRMS data interpretation. ^a^ Residues R^1^‒R^4^ refer to the carbamido-/cylindrocyclophane core structure as depicted in Figure 2D.

Peak ^b^	Molecular Formula	[M − H]^−^ *m/z*		Δ ^c^ (ppm)	Iso Score	DBE	R^1^	R^2^	R^3^	R^4^	Compound, [Reference of First Structure Elucidation]
		Meas.	Pred.								
*Brominated derivatives*											
**1**	C_38_H_57_BrN_2_O_8_	747.3216 ^d^	747.3226	1.3	88.0	11	OCONH_2_	CH_3_	OCONH_2_	CH_2_Br	Carbamidocyclophane M (**1**) ^e^, [t.s.]
**2**	C_38_H_56_Br_2_N_2_O_8_	825.2336 ^d^	825.2331	0.6	100	11	OCONH_2_	CH_2_Br	OCONH_2_	CH_2_Br	Carbamidocyclophane N (**2**) ^e^, [t.s.]
**3**	C_38_H_56_Br_2_N_2_O_8_	825.2340 ^d^	825.2331	1.1	100	11	OCONH_2_	CH_3_	OCONH_2_	CHBr_2_	Carbamidocyclophane O (**3**) ^e^, [t.s.]
**4**	C_38_H_55_Br_3_N_2_O_8_	903.1425 ^d^	903.1436	1.2	100	11	OCONH_2_	CH_2_Br	OCONH_2_	CHBr_2_	Carbamidocyclophane P (**4**) ^e^, [t.s.]
**5**	C_38_H_54_Br_4_N_2_O_8_	981.0529 ^d^	981.0541	1.2	99.1	11	OCONH_2_	CHBr_2_	OCONH_2_	CHBr_2_	Carbamidocyclophane Q (**5**) ^e^, [t.s.]
**6**	C_37_H_56_BrNO_7_	704.3173 ^d^	704.3167	0.9	100	10	OCONH_2_	CH_3_	OH	CH_2_Br	Carbamidocyclophane R (**6**) ^e^, [t.s.]
**7**	C_37_H_55_Br_2_NO_7_	782.2266 ^d^	782.2272	0.8	95.0	10	OCONH_2_	CH_3_	OH	CHBr_2_	Carbamidocyclophane S (**7**) ^e^, [t.s.]
**8**	C_37_H_54_Br_3_NO_7_	860.1382 ^d^	860.1378	0.5	95.6	10	OCONH_2_	CH_2_Br	OH	CHBr_2_	Carbamidocyclophane T (**8**) ^e^, [t.s.]
**9**	C_37_H_53_Br_4_NO_7_	938.0499 ^d^	938.0483	1.7	100	10	OCONH_2_	CHBr_2_	OH	CHBr_2_	Carbamidocyclophane U (**9**) ^e^, [t.s.]
37	C_37_H_56_BrNO_6_	688.3207	688.3218	1.6	100	10	OCONH_2_	CH_3_	H	CH_2_Br	Putative new [7.7]paracyclophane ^f^
							H	CH_3_	OCONH_2_	CH_2_Br	
38	C_37_H_53_Br_2_NO_8_	796.2059	796.2065	0.8	87.6	11	OCONH_2_	CH_2_OH	O	CHBr_2_	Putative new [7.7]paracyclophane ^f^
							OCONH_2_	CHBrOH	O	CH_2_Br	
							O	CH_2_OH	OCONH_2_	CHBr_2_	
							O	CHBrOH	OCONH_2_	CH_2_Br	
39 40	C_37_H_55_Br_2_NO_6_	766.2316 766.2322	766.2323	0.9 0.1	85.4 92.7	10 10	OCONH_2_	CH_2_Br	H	CH_2_Br	Putative new [7.7]paracyclophanes ^f^
							OCONH_2_	CH_3_	H	CHBr_2_	
							H	CH_3_	OCONH_2_	CHBr_2_	
41	C_37_H_54_Br_3_NO_6_	844.1424	844.1428	0.5	97.5	10	OCONH_2_	CH_2_Br	H	CHBr_2_	Putative new[7.7]paracyclophane ^f^
							H	CH_2_Br	OCONH_2_	CHBr_2_	
42	C_37_H_53_Br_4_NO_6_	922.0541	922.0534	0.8	100	10	OCONH_2_	CHBr_2_	H	CHBr_2_	Putative new [7.7]paracyclophane ^f^
43	C_36_H_55_BrO_6_	661.3077	661.3109	4.8	79.5	9	OH	CH_3_	OH	CH_2_Br	Putative new [7.7]paracyclophane ^f^
*Chlorinated derivatives*											
**10**	C_38_H_57_ClN_2_O_8_	703.3731	703.3731	0.0	100	11	OCONH_2_	CH_3_	OCONH_2_	CH_2_Cl	Carbamidocyclophane D (**10**) ^e^, [5]
**11**	C_38_H_56_Cl_2_N_2_O_8_	737.3332	737.3341	1.2	87.5	11	OCONH_2_	CH_2_Cl	OCONH_2_	CH_2_Cl	Carbamidocyclophane J (**11**) ^e^, [7]
**12**	C_38_H_56_Cl_2_N_2_O_8_	737.3339	737.3341	0.3	88.9	11	OCONH_2_	CH_3_	OCONH_2_	CHCl_2_	Carbamidocyclophane C (**12**) ^e^, [5]
**13**	C_38_H_55_Cl_3_N_2_O_8_	771.2966	771.2951	1.9	91.1	11	OCONH_2_	CH_2_Cl	OCONH_2_	CHCl_2_	Carbamidocyclophane B (**13**) ^e^, [5]
**14**	C_38_H_54_Cl_4_N_2_O_8_	805.2559	805.2562	0.4	92.4	11	OCONH_2_	CHCl_2_	OCONH_2_	CHCl_2_	Carbamidocyclophane A (**14**) ^e^, [5]
**15**	C_37_H_56_ClNO_7_	660.3672	660.3673	0.2	96.7	10	OCONH_2_	CH_3_	OH	CH_2_Cl	Carbamidocyclophane I (**15**) ^e^, [7]
**16**	C_37_H_55_Cl_2_NO_7_	694.3269	694.3283	2.0	89.2	10	OCONH_2_	CH_3_	OH	CHCl_2_	Carbamidocyclophane K (**16**) ^e^, [7]
**17**	C_37_H_54_Cl_3_NO_7_	728.2903	728.2893	1.2	86.4	10	OCONH_2_	CH_2_Cl	OH	CHCl_2_	Carbamidocyclophane L (**17**) ^e^, [7]
**18**	C_37_H_53_Cl_4_NO_7_	762.2499	762.2503	0.5	100	10	OCONH_2_	CHCl_2_	OH	CHCl_2_	Carbamidocyclophane F (**18**) ^e^, [6]
**21**	C_36_H_55_ClO_6_	617.3626	617.3614	1.9	93.2	9	OH	CH_3_	OH	CH_2_Cl	Cylindrocyclophane A_1_(**21**) ^e^, [8]
**22**	C_36_H_54_Cl_2_O_6_	651.3219	651.3225	0.9	80.4	9	OH	CH_3_	OH	CHCl_2_	Cylindrocyclophane A_2_ (**22**) ^e^, [8]
**23**	C_36_H_53_Cl_3_O_6_	685.2828	685.2835	1.0	84.8	9	OH	CH_2_Cl	OH	CHCl_2_	Cylindrocyclophane A_3_ (**23**) ^e^, [8]
**24**	C_36_H_52_Cl_4_O_6_	719.2434	719.2445	1.5	86.9	9	OH	CHCl_2_	OH	CHCl_2_	Cylindrocyclophane A_4_ (**24**) ^e^, [8]
31	C_36_H_55_ClO_5_	601.3665	601.3665	0.5	99.3	9	OH	CH_3_	H	CH_2_Cl	Cylindrocyclophane C_1_, [8]
32	C_36_H_54_Cl_2_O_5_	635.3273	635.3276	0.5	95.6	9	OH	CH_3_	H	CHCl_2_	Cylindrocyclophane C_2_, [8]
33	C_36_H_53_Cl_3_O_5_	669.2898	669.2886	1.8	100	9	OH	CH_2_Cl	H	CHCl_2_	Cylindrocyclophane C_3_, [8]
34	C_36_H_52_Cl_4_O_5_	703.2485	703.2496	1.6	100	9	OH	CHCl_2_	H	CHCl_2_	Cylindrocyclophane C_4_, [8]
44	C_37_H_56_ClNO_6_	644.3724	644.3723	0.2	100	10	OCONH_2_	CH_3_	H	CH_2_Cl	Putative new [7.7]paracyclophane ^f^
							H	CH_3_	OCONH_2_	CH_2_Cl	
45	C_37_H_55_Cl_2_NO_7_	694.3264	694.3283	2.7	84.3	10	OCONH_2_	CH_2_Cl	OH	CH_2_Cl	Putative new [7.7]paracyclophane ^f^
							OH	CH_3_	OCONH_2_	CHCl_2_	
46 47	C_37_H_55_Cl_2_NO_6_	678.3330 678.3332	678.3334	0.6 0.3	100 100	10 10	OCONH_2_	CH_2_Cl	H	CH_2_Cl	Putative new [7.7]paracyclophanes ^f^
							OCONH_2_	CH_3_	H	CHCl_2_	
							H	CH_3_	OCONH_2_	CHCl_2_	
48	C_37_H_54_Cl_3_NO_6_	712.2952	712.2944	1.1	87.1	10	OCONH_2_	CH_2_Cl	H	CHCl_2_	Putative new [7.7]paracyclophane ^f^
							H	CH_2_Cl	OCONH_2_	CHCl_2_	
49	C_37_H_53_Cl_4_NO_6_	746.2559	746.2554	0.3	100	10	OCONH_2_	CHCl_2_	H	CHCl_2_	Putative new [7.7]paracyclophane ^f^
50	C_36_H_54_Cl_2_O_6_	651.3223	651.3225	0.9	79.3	9	OH	CH_2_Cl	OH	CH_2_Cl	Putative new [7.7]paracyclophane ^f^
51	C_36_H_54_Cl_2_O_5_	635.3275	635.3276	0.2	100	9	H	CH_3_	OH	CHCl_2_	Putative new [7.7]paracyclophane ^f^
							OH	CH_2_Cl	H	CH_2_Cl	
52	C_36_H_54_Cl_2_O_4_	619.3323	619.3326	0.5	100	9	H	CH_2_Cl	H	CH_2_Cl	Putative new [7.7]paracyclophane ^f^
							H	CH_3_	H	CHCl_2_	
*Non-halogenated derivatives*											
**19**	C_38_H_58_N_2_O_8_	669.4122	669.412	0.3	100	11	OCONH_2_	CH_3_	OCONH_2_	CH_3_	Carbamidocyclophane E (**19**) ^e^, [5]
**20**	C_37_H_57_NO_7_	626.4064	626.4062	0.3	100	10	OCONH_2_	CH_3_	OH	CH_3_	Carbamidocyclophane H (**20**) ^e^, [7]
**25**	C_36_H_56_O_6_	583.3994	583.4004	1.7	83.1	9	OH	CH_3_	OH	CH_3_	Cylindrocyclophane A (**25**) ^e^, [4]
35	C_36_H_56_O_5_	567.4004	567.4055	2.3	89.7	9	OH	CH_3_	H	CH_3_	Cylindrocyclophane C, [9]
36	C_36_H_56_O_4_	551.4102	551.4106	0.7	100	9	H	CH_3_	H	CH_3_	Cylindrocyclophane F, [9]
53	C_37_H_55_NO_7_	624.3888	624.3906	2.9	88.7	11	OCONH_2_	CH_3_	O	CH_3_	Putative new [7.7]paracyclophane ^f^
54	C_37_H_57_NO_6_	610.4113	610.4113	0.0	100	10	OCONH_2_	CH_3_	H	CH_3_	Putative new [7.7]paracyclophane ^f^

^a^ Abbreviations: Meas. = measured, Pred. = predicted, DBE = double bond equivalent, t.s. = this study; ^b^ Peak numbers in bold indicate compounds that were also evaluated for biological activity in this study (see Subsection 2.5); ^c^ Δ = relative mass error; ^d^ The measured accurate mass of the isolated compound is presented in Subsection 3.2.5. of the experimental section; ^e^ Compound identification was additionally confirmed by comparison to authentic standard; ^f^ Structural proposal: Indication of the residue positions is arbitrary and refers to the most likely substitution pattern.

### 2.2. Isolation and Structure Elucidation of Brominated Carbamidocyclophanes (**1**‒**9**)

For the isolation of the observed bromo-analogues, we scaled up the cultivation conditions to a 36 L column fermentation in Z½ medium as described previously [7]. According to the initial results of the halide salt feeding experiments, KBr was added to a final concentration of 0.1% two weeks after inoculation of the cyanobacterium. Cultivation was continued for another four weeks to obtain a maximal yield of brominated compounds. The extraction and enrichment procedure of bromo-carbamidocyclophanes was performed using the recently described biphasic solvent system [38]. The [7.7]paracyclophane-containing lower-phase-fraction was subjected to semi-preparative HPLC using our two-column-strategy as reported for the isolation of chlorinated carbamidocyclophane analogues [7] to yield compounds **1**‒**9**.

Based on the previous elucidation of 12 chlorinated carbamidocyclophanes [5,6,7], structure elucidation of the isolated bromo-analogues **1**‒**9** was straightforward. Interpretation of the tandem HRMS data indicated the degree of bromination of the compounds via the isotope pattern as well as the degree of carbamoylation (losses of 43 Da). HRMS data of the isolated compounds, named carbamidocyclophanes M‒U, as well as the deduced molecular formulas can be found in Table 1 and in the Experimental Section (see Section 3.2.5).

**Figure 3:**
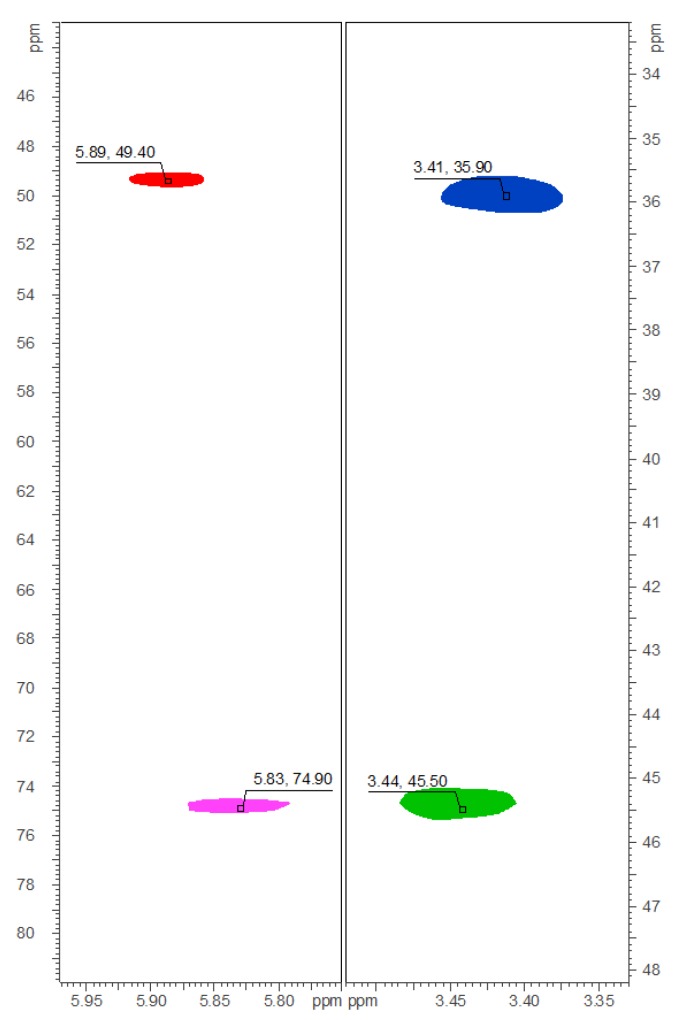
Details of the collated HMQC-DEPT-NMR spectra of **8** (red for CH-30, δ_C/H_ 49.4/5.89; blue for CH_2_-34, δ_C/H_ 35.9/3.41) and **17** (magenta for CH-30, δ_C/H_ 74.9/5.83; green for CH_2_-34, δ_C/H_ 45.6/3.43).

The ^1^H-, HMQC-DEPT-, and HMBC-NMR spectra of **1**‒**9** closely resembled the spectra of the respective chlorinated analogues. Substitution of chlorine by bromine at positions C-30 and C-34—as could be expected—mainly influenced the ^13^C chemical shift of the respective carbons, as the deshielding effect of bromine is smaller than that of chlorine. This is exemplified in Figure 3 for compound **8**: C-30 of carbamidocyclophane T (**8**), bearing two bromine atoms, resonates at δ_C_ 49.3, compared with δ_C_ 74.9 in the case of the dichlorinated analogue carbamidocyclophane L (**17**) [7]. C-34, featuring one bromine atom, is observable at δ_C_ 35.9 *vs.* δ_C_ 45.6 in **17** (Figure 3). All carbon and proton assignments (Table S3 and S4) were done in analogy to those described before in great detail for the chlorinated carbamidocyclophanes [5,6,7].

**Figure 4:**
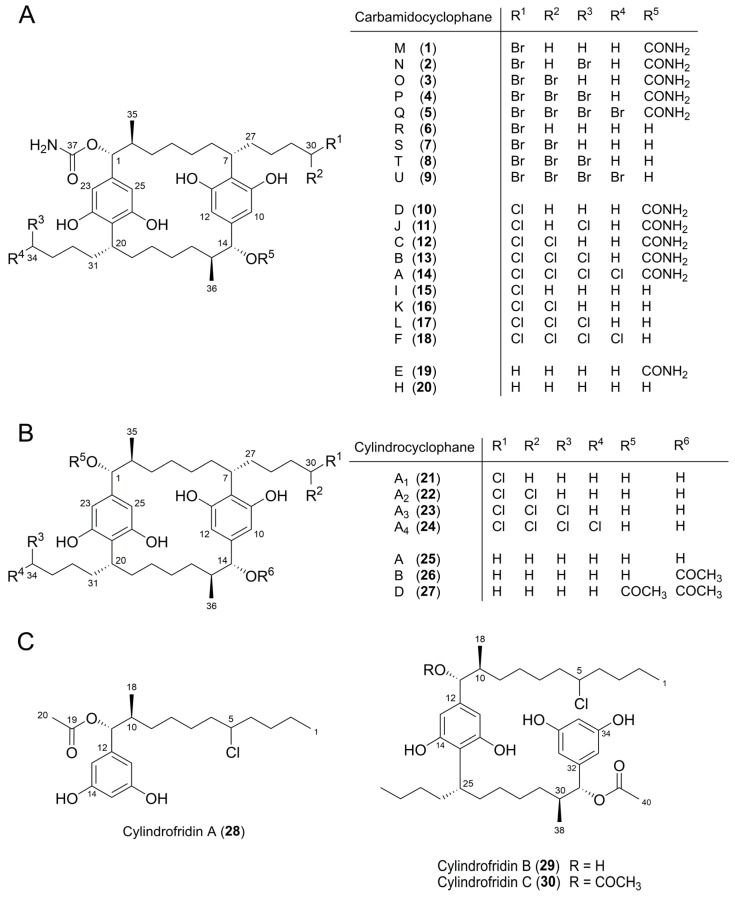
Compounds **1**‒**30** used for the determination of structure–activity relationships (SAR). (**A**) Carbamidocyclophanes. (**B**) Cylindrocyclophanes. (**C**) Cylindrofridins.

The stereoconfigurations at C-1, C-2, C-7, C-14, C-15, and C-20 of **1**‒**9** were determined by a combination of coupling constant analysis and comparison of the ECD spectra with those of carbamidocyclophane analogues. The ^3^*J*_H-1/H-14,H-2/H-15_ values for **1**‒**9** (9.5‒10.6 Hz) were in the same range as those reported for the chlorinated congeners **10**‒**18** [5,6,7]. This similarity indicates that H-1/H-14 and H-2/H-15 of brominated carbamidocyclophanes are in anti-conformation as well. The absolute configurations of **1**‒**9** were established comparing the respective ECD spectra to those of the chlorinated carbamidocyclophanes. ECD spectra of **1**‒**5** were similar to those of **10**‒**14** [7], revealing a positive Cotton effect in the range of 224‒237 nm (Δε from 0.89 to 1.80) and a negative Cotton effect in the range of 273−282 nm (Δε from −0.38 to −1.21). Furthermore, compounds **6**‒**9** showed comparable spectra with a negative Cotton effect in the range 217‒222 (Δε from ‒0.61 to ‒3.84) and a second negative Cotton effect in the range 273‒280 nm (Δε from −0.37 to −1.11) to those reported for **15**‒**18** [6,7]. Due to these data, we conclude that **1**‒**9** have the same absolute configuration at the six stereogenic carbons as **10**‒**18**, namely 1*R*, 2*S*, 7*R*, 14*R*, 15*S*, and 20*R* (Figure 4A).

### 2.3. Quantification of Carbamidocyclophanes (**1**‒**20**)

Our initial screening data revealed that the unknown halogenase involved in [7.7]paracyclophane biosynthesis of *Nostoc* sp. CAVN2 can utilize chloride as well as bromide as the halide substrate. This is in agreement with results of previous studies on halogenated natural products, for which an involvement of FADH_2_-dependent halogenases is discussed, e.g., for chloramphenicol [23], chlortetracycline [22], pyrrolnitrin [26], and balhimycin [30], and seems to be widespread among this class but has also been reported for other halogenating enzymes [39,40]. Thus, we investigated the growth and the compound biosynthesis in the presence of KBr and KCl in detail with an emphasis on the quantification of every single carbamidocyclophane **1**‒**20** to evaluate the halide preference of the halogenating enzyme on the metabolic level.

**Figure 5:**
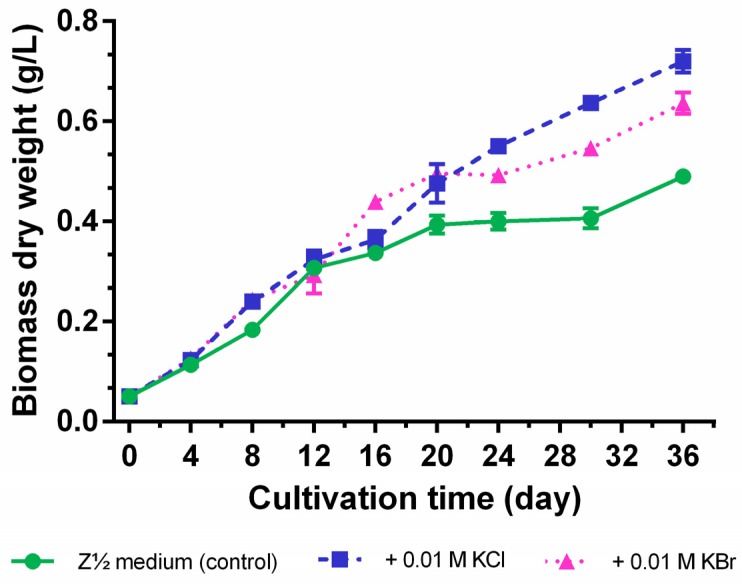
Growth curves of *Nostoc* sp. CAVN2 cultivated in halide-enriched medium. Values shown are expressed as the mean ± SEM, *n* = 3.

Cultivation in the presence of equimolar chloride and bromide concentrations (0.01 M) for 36 days showed that the supplementation of these halide salts positively influenced cyanobacterial growth (maximum growth rate *µ*_max_ = 0.24 ± 0.04 day^−1^ for 0.01 M KCl, *µ*_max_ = 0.24 ± 0.03 day^−1^ for 0.01 M KBr, and *µ*_max_ = 0.21 ± 0.05 day^−1^ for the control). In addition, the dry biomass yielded a maximum of 0.72 ± 0.02 g/L for the KCl-enrichment and 0.64 ± 0.02 g/L for KBr-enrichment, but only 0.49 ± 0.02 g/L for the control (Z½ medium without additional halide salt supplementation) at the end of the cultivation period (Figure 5). Especially, the control culture already left the log phase and entered a stationary phase after 16−20 days, but revealed again a slight increase in biomass production within the last six days. As this hampering effect on cyanobacterial growth was less pronounced for the KBr-supplementation and not observed for the KCl-enriched culture, we conclude that in particular the nature of the halide anion, and not potassium as the halide counterion, is responsible for this phenomenon.

**Figure 6:**
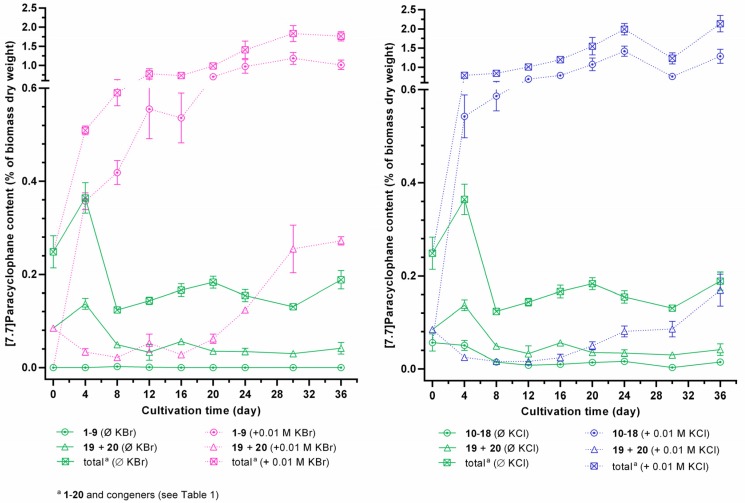
Intracellular [7.7]paracyclophane contents of *Nostoc* sp. CAVN2 cultivated either in bromide-enriched (+ 0.01 M KBr), chloride-enriched (+ 0.01 M KCl) or in unmodified (<0.1 µM halide ions, Ø KBr/Ø KCl) Z½ medium. Values shown are expressed as the mean ± SEM, *n* = 3.

Quantification of halogenated carbamidocyclophanes **1**−**18** generally showed similar profiles in the presence of halide ions in the cultivation medium (Figures S1 and S2), *i.e.*, a continuous increase up to a maximum content between day 24 and 36. In this time range, the average total content of chlorinated carbamidocyclophanes was nearly the same as determined for brominated carbamidocyclophanes (1.26% ± 0.21% and 1.27% ± 0.10%, respectively, of biomass dry weight). In addition, the average total [7.7]paracyclophane contents were also not significantly different between the halide treatments. As depicted in Figure 6, however, compound contents in KCl-enriched medium showed an up to 1.9-fold decrease on day 30 compared to day 24 or day 36, similar as previously seen for the quantification of carbamidocyclophanes A−E in *Nostoc* sp. CAVN10 biomass [37]. This was not observed for the KBr-enriched samples. Among individual carbamidocyclophanes, chlorinated carbamidocyclophane A (**14**) (0.59% ± 0.03% of dry biomass weight on day 24) and C (**12**) (0.28% ± 0.02% on day 36) as well as brominated Q (**5**) (0.44% ± 0.05% on day 30) and O (**3**) (0.43% ± 0.02% on day 30) were most abundant (Figures S1 and S2).

As already observed for total [7.7]paracyclophane amount in the initial halide screening, the absence of chloride and bromide resulted in an up to 50-fold reduction of the carbamidocyclophane/total [7.7]paracyclophane content and prevented the formation of equivalent amounts of non-halogenated derivatives (Figure 6, Figures S1 and S2). This is in contrast to what has been observed for chlortetracycline biosynthesis [22]. Moreover, also non-halogenated carbamidocyclophanes, in particular carbamidocyclophane E (**19**), were increasingly biosynthesized in the presence of chloride or bromide (Figures S1 and S2).

**Figure 7:**
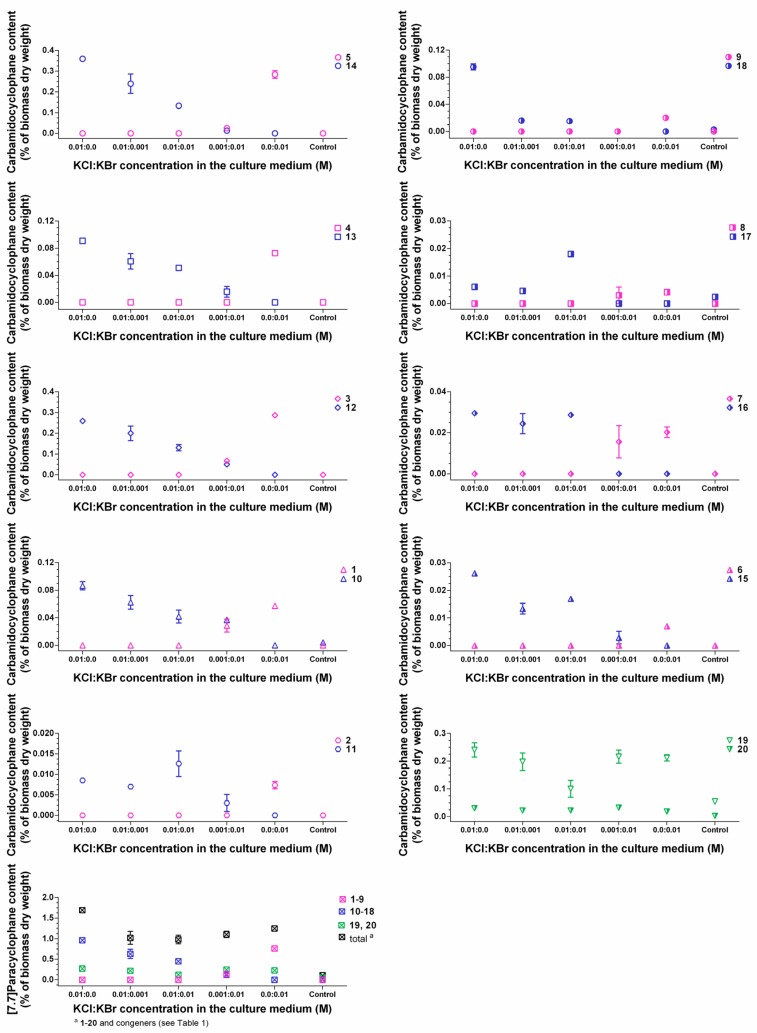
Comparison of intracellular contents between brominated (**1**‒**9**), chlorinated (**10**‒**18**), and non-halogenated (**19**, **20**) carbamidocyclophanes in *Nostoc* sp. CAVN2 biomass cultivated in the presence of KCl-KBr-mixtures. Values shown are expressed as the mean ± SEM, *n* = 3.

For evaluating the incorporation efficiency of bromine and chlorine, we further cultured strain CAVN2 in the presence of both halides at different mixing ratios (Figure 7). The quantification results of **1**−**20** revealed that bromide competed with chloride depending on the ratio of both ions in the medium. Chloride is the preferred halide ion for incorporation into the carbamidocyclophane-scaffold. Only minor chloro-carbamidocyclophanes **11**, **15**, **16**, and **17** were less affected by the presence of bromide and showed partly a slight increase of content. However, *Nostoc* sp. CAVN2 was less able to accept bromide for the production of bromo-analogues **1**−**9** in the presence of chloride. The cyanobacterium utilized bromide only to co-produce these compounds in small amounts when present at the highest concentration ratio tested, *i.e.*, KBr:KCl = 10:1. Notably at this ratio, HPLC-MS analysis indicated the presence of a hybrid derivative. At equimolar concentrations, however, **1**−**9** were not detected, but this ratio revealed a significantly improved growth of the strain (Table S5). Similar effects of chloride and bromide have previously been reported for the fermentation of ochratoxin [41], chloramphenicol [24], salinosporamide [42] and monamycin [27]. On the other hand, the opposite is reported for the balhimycin biosynthesis, *i.e.*, supplementation with equimolar amounts of bromide and chloride resulted in chloro-, bromo- and even chlorobromobalhimycin without any preference for either halide [30]. Likewise to the ochratoxin and chloramphenicol production, balhimycin halogenation is most likely mediated through a FADH_2_-dependent halogenase [40]. Thus, the preference of a certain halide seems to depend highly on the respective protein itself, and the outcome of a resulting halogenation event is very strain-specific.

### 2.4. Identification of the Carbamidocyclophane Gene Cluster in Nostoc sp. CAVN2

The [7.7]paracyclophane diversity in *Nostoc* sp. CAVN2 encouraged us not only to investigate carbamidocyclophane biosynthesis on the metabolite level but also on the genomic level. Whole genome shotgun sequencing of CAVN2 DNA resulted in 208,054 total reads with an average read length of 356 nt. After assembly, the draft genome consisted of 682 contigs with a size of at least 500 nucleotides. The MegaBLAST search of the cylindrocyclophane biosynthetic gene cluster (acc. no. JX477167) against a local BLAST+ database resulted in significant hits on three contigs (contig00129, contig00638 and contig00697) of the *Nostoc* sp. CAVN2 draft genome. By mapping these contigs to the reference cluster, their order and orientation towards each other and the approximate distance between them was revealed. While the gap between contig00638 and contig00697 actually spanned the proposed 10 bp, amplification and sequencing of the other gap led to a 652 bp region between contig00129 and contig00638, as opposed to the predicted 175 bp. This longer region is due to an additional ACP domain in the protein-coding gene *cabD*. After the first functional assessment, two frameshifts were discovered in the coding regions for CabC and CabD, which resulted in a premature stop codon, and a second overlapping downstream open reading frame (ORF) at both sites. Closer investigation revealed that both frameshifts occurred in a homopolymer region. Pyrosequencing reads are prone to incorrect determination of length at these sites, thus leading to insertions or deletions by over- or undercalled bases [43]. We refuted the putative frameshifts by resequencing the questionable regions with the Sanger chain-termination method [44]. A single additional nucleotide at both sites reconstituted the correct full-length ORFs.

**Figure 8:**
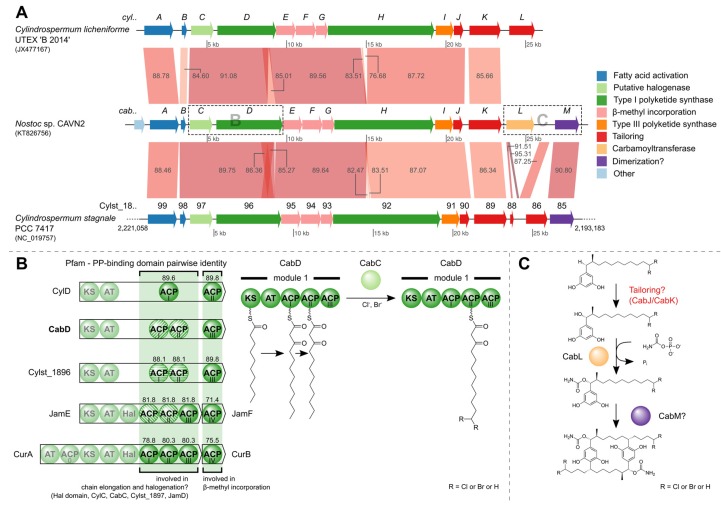
The carbamidocyclophane (*cab*) biosynthetic gene cluster. (**A**) MegaBLAST comparison of gene clusters with shared synteny. The gene loci are colored according to functional categories given on the right. Numbers on the synteny blocks indicate percent identity. For *C. stagnale* PCC 7417, the compared region is a subset of the complete genome, as indicated by the genome positions at the region ends; (**B**) The PP-binding domain (Pfam) contains the active site serine residue to which the 4′-phosphopantetheine is attached as prosthetic group. Pairwise identities between the CabD PP-binding domains of ACP_I_/ACP_II_ and ACP_III_, and their homologues are depicted above the ACP domains. Domains with 100% identity are illustrated with the same striped pattern. The halogenation by the putative halogenase CabC might take place while the nascent polyketide is bound to ACP_II_. Abbreviations: AT = acyl transferase, ACP = acyl carrier protein, KS = beta-ketoacyl synthase, Hal = halogenase; (**C**) Putative actions of the carbamoyltransferase encoded by *cabL* and the Rieske [2Fe-2S] iron-sulphur domain-containing protein CabM.

The final contig has a size of 28,341 nucleotides and harbors 14 open reading frames, 13 of which presumably constitute the gene cluster required for biosynthesis of carbamidocyclophanes (*cab*, Table S6). Its sequence has been deposited in GenBank under accession number KT826756. The cluster shares synteny with two clusters that are involved in biosynthesis of the chemically related cylindrocyclophanes. More precisely, nucleotide comparisons employing MegaBLAST searches showed that most of the cluster shares at least 76% and 82% sequence similarity with clusters from *C. licheniforme* UTEX ‘B 2014’ and *Cylindrospermum stagnale* PCC 7417, respectively (Figure 8A, Tables S7 and S8). One striking difference is the presence of the carbamoyltransferase CabL. In *Nostoc* sp. CAVN2 the gene locus coding for this key enzyme of carbamidocyclophane biosynthesis can be found in close proximity to other putative tailoring enzymes, whereas it is absent in the otherwise related biosynthetic gene clusters of *Cylindrospermum* species. Nakamura *et al.* [17] discussed the possibility of a dimerization event catalyzed by Rieske oxygenase homologues, which have been shown to form C-C bonds *in vivo*. Interestingly, both clusters of *C. stagnale* PCC 7417 and *Nostoc* sp. CAVN2 possess a gene-coding region (*cabM*, Cylst_1885) downstream of *cabL* which contains a Rieske [2Fe-2S] iron-sulphur domain. The presence of this ORF in *Nostoc* sp. CAVN2 corroborates our previous hypothesis that this enzyme might be responsible for the final macrocyclization event [38]. The assumed catalytic actions carried out by CabL and CabM are depicted in Figure 8C. In order to confirm the postulated carbamoyltransferase activity of CabL, a knockout of the corresponding gene was started according to a protocol for conjugal transfer of DNA to cyanobacteria [45]. However, since *Nostoc* sp. CAVN2 has not been transformed before, this work requires some adaptation and is still in progress.

Another difference that is readily visible in Figure 8A is the aforementioned size discrepancy between CabD and CylD. While CabD and the protein encoded by the Cylst_1896 gene locus differ by 28 amino acids (1385 aa *vs.* 1357 aa), a size difference of 157 aa (CylD: 1228 aa) led us to examine the domain organization more closely. Figure 8B (left) shows the protein domains of the type I polyketide synthases encoded by *cylD*, *cabD* and Cylst_1896 as well as proteins from two other biosynthetic gene clusters (*jam*, *cur*) [46,47]. The Pfam PP-binding domains of all depicted proteins were aligned and the pairwise identities with reference to the ACP_I_/ACP_II_ and ACP_III_ domains of CabD are shown. Besides their participation in chain elongation, ACPs are supposedly involved in other biosynthetic reactions. They are involved in β-methyl incorporation [48] and also serve as attachment site during halogenation by a *cis*-acting halogenase domain (Hal) as shown for curacin A [49]. Notably, tandem di- or tri-domain ACPs have been shown to promote consecutive multi-enzyme reactions that have a synergistic effect [50]. This division of work might also hold true for the ACP domains of the three carbamidocyclophane/cylindrocyclophane clusters (Figure 8B, bottom) and explain the fact, that so far no halogenated cylindrocyclophanes have been isolated from *C. licheniforme* UTEX ‘B 2014’. For though an ORF with similarity to recently described putative halogenases (ColD, ColE) [20] is found in the *cyl* gene cluster (CylC) [18], the type I PKS has only one ACP domain involved in chain elongation. The second ACP actually resembles the free-standing ACPs JamF and CurB and is involved in the β-methyl incorporation, as is the ACP_III_ of CabD and the Cylst_1896 protein.

Based on the putative halogenases ColD and ColE, further recent studies have proposed the existence of a halogenase (JamD, BrtJ) [21,47] within their described biosynthetic gene clusters. We created a multiple sequence alignment using most of the described homologues and further proteins obtained by a BlastP search (Figure S3). The included AurF Chain A, a *p*-aminobenzoate *N*-oxygenase (AurF) of *Streptomyces thioluteus,* contains several conserved glutamic acid and histidine residues that coordinate a di-iron core. All but one (annotated with Glu-196) of the seven sites show 100% conservation among all considered sequences. This might indicate a different mode of action than what is known for mononuclear non-heme iron and α-ketoglutaric acid-dependent halogenases [51] and lead to further investigations in the field.

### 2.5. Biological Evaluation of Extracts and Brominated Carbamidocyclophanes (**1**‒**9**) as well as Other Congeners (**10**‒**30**)

The initial screening for biological evaluation of the CAVN2 methanol extract after cultivation in KBr-enriched medium was carried out using a set of pathogenic bacteria such as methicillin-resistant *Staphylococcus aureus* (MRSA) 1, *Streptococcus pneumoniae* 7 (also known as ATCC 49619), *Escherichia coli* 13, kanamycin-resistant *Klebsiella pneumoniae* (KRKP) 18, and multidrug-resistant (MDR) *Pseudomonas aeruginosa* 22 as well as non-tumorigenic HaCaT cells (Table 2). In accordance with previous bioactivity results of [7.7]paracyclophane-containing crude samples [7,38], the extract revealed remarkable activity against the Gram-positive pathogens, but not against the Gram-negative bacteria. This extract showed stronger cytotoxicity compared to previously reported data of both the carbamido- and cylindrocyclophane-containing extract from *Nostoc* sp. CAVN2 and the cylindrofridin- and cylindrocyclophane-containing extract from *C. stagnale* PCC 7417 [7,38].

**Table 2:** Biological activity of [7.7]paracyclophane-containing extracts ^a^.

	MIC/IC_50_ (μg/mL)			
	*Nostoc* sp. CAVN2 Extract (KBr enriched) ^b^	*Nostoc* sp. CAVN2 Extract ^c^	*C. stagnale* PCC 7417 Extract ^d^	POS
*A. baumannii* DSM-30008	>1000	>1000	>1000	n.t.
*B. cenocepacia* DSM-16553	>1000	>1000	>1000	n.t.
*E. aerogenes* DSM-30053	>1000	>1000	>1000	0.2 ^e^
*E. coli* 13	>50	>50	>50	0.0062 ^e,f,g^, 62.5 ^h^
*E. coli* DSM-1116	>1000	>1000	>1000	0.01 ^e^
*E. coli* (TolC-deficient)	>1000	>1000	>1000	0.003 ^e^
*K. pneumoniae* 18 (KRKP)	>50	>50	>50	1.25 ^e,g^, 0.62 ^f^
*P. aeruginosa* 22 (MDR)	>50	>50	>50	0.025 ^g^, 250 ^h^
*P. aeruginosa* DSM-1128	>1000	>1000	>1000	0.1 ^e^
*E. faecium* DSM-20477	31.3	31.3	31.3	2.0 ^h^
*E. faecium* DSM-17050 (VREF)	15.6	7.8	7.8	>64 ^h^
*M. bovis* DSM-43990 (BCG)	<200	<200	<200	n.t.
*M. smegmatis* mc^2^ 155	500	250	125	0.125 ^i^
*N. asteroides* DSM-43757	7.8	15.6	31.3	8.0 ^j^, 0.25 ^k^, 2.0 ^l^
*S. aureus* 1 (MRSA)	0.1	0.8	0.04−0.08	2.0 ^h,m^
*S. aureus* DSM-11822 (MRSA)	1	1	1	1.0 ^h^
*S. aureus* M50 (MRSA/VISA)	0.5	1	1	16.0 ^h^
*S. aureus* Newman (MSSA)	0.5	1	1	0.5 ^h^
*S. aureus* N315 (MRSA)	1	1	1	1.0 ^h^
*S. carnosus* DSM-20501	<0.5	1	1	0.25 ^h^
*S. pneumoniae* 7 (ATCC 49619)	0.2	3.2	0.2	2.0 ^h,m^
*S. pneumoniae* DSM-20566	62.5	15.6	31.3	<0.03 ^n^
*S. pneumoniae* DSM-11865 (PRSP)	31.3	62.5	62.5	>64 ^n^
*C. albicans* DSM-1665	>1000	>1000	>1000	67°
HaCaT cells	0.9	14.0	2.8	1.7 ^p^

^a^ Abbreviations: POS = positive control, KRKP = kanamycin-resistant *K. pneumoniae*, MDR = multi-drug resistant (for detailed resistance profile, see Pretsch *et al.* [52]), VREF = vancomycin-resistant *E. faecium*, BCG = bacillus Calmette-Guérin, MRSA = methicillin-resistant *S. aureus*, VISA = vancomycin-intermediate *S. aureus*, MSSA = methicillin-sensitive *S. aureus*, PRSP = penicillin-resistant *S. pneumoniae*, n.t. = not tested; ^b^ Extract contains especially brominated carbamidocyclophanes **1**‒**9**; ^c^ Extract contains a wide array of chlorinated and non-halogenated carbamidocyclophanes and cylindrocyclophanes; Data shown against *S. aureus* 1, *S. pneumoniae* 7, *E. coli* 13, *K. pneumoniae* 18, *P. aeruginosa* 22, and HaCaT cells have previously been reported; For further details, see Preisitsch *et al.* [7]; ^d^ Extract contains various cylindrocyclophanes and cylindrofridins; Data shown against *S. aureus* 1, *S. pneumoniae* 7, *E. coli* 13, *K. pneumoniae* 18, *P. aeruginosa* 22, and HaCaT cells have previously been reported; For further details, see Preisitsch *et al.* [38]; ^e^ ciprofloxacin; ^f^ moxifloxacin; ^g^ levofloxacin; ^h^ vancomycin; ^i^ gentamicin; ^j^ erythromycin; ^k^ imipenem; ^l^ tobramycin; ^m^ fusidic acid; ^n^ ampicillin; °nystatin; ^p^ mitoxantrone.

Due to these results and a reported antifungal as well as anti-*Enterococcus* and anti-*Mycobacterium* activity by Luo *et al.* [6], we extended the panel of microorganisms to various drug-susceptible and drug-resistant indicator strains, such as *Enterococcus faecium*, *Mycobacterium bovis* and *Mycobacterium smegmatis*, *Nocardia asteroides*, *Staphylococcus carnosus*, *Acinetobacter baumannii*, *Burkholderia cenocepacia*, *Enterobacter aerogenes* and *Candida albicans.* Furthermore, we included additional *S. aureus*, *S. pneumoniae*, *E. coli* and *P. aeruginosa* strains. In summary, all three extracts showed no activity against any of the Gram-negative bacteria and were not antifungal. In contrast, the extracts revealed strong activity against most of the Gram-positive bacteria, including drug-resistant strains, e.g., against *E. faecium* (MICs in the range 7.8‒31.3 µg/mL) and *S. pneumoniae* (MICs in the range 15.6‒62.5 µg/mL), but in particular against *S. aureus* (MICs of ≤0.5‒1 µg/mL) (Table 2).

Subsequently, we selected drug-susceptible *E. faecium*, vancomycin-resistant *E. faecium* (VREF), methicillin-sensitive *S. aureus* (MSSA) several MRSA strains and a vancomycin-intermediate *S. aureus* (VISA) isolate, drug-susceptible *S. pneumoniae*, and penicillin-resistant *S. pneumoniae* (PRSP) for a structure–activity relationship (SAR) study of purified compounds. The compound set included 30 [7.7]paracyclophanes and related congeners (Figure 4), namely carbamidocyclophanes **1**‒**20**, cylindrocyclophanes (**21**‒**27**), and cylindrofridins **28**‒**30**. Although the MIC values of extracts against *Mycobacterium* spp. were rather high compared with those obtained against the other Gram-positive bacteria, we also included virulent *Mycobacterium tuberculosis* strain H37Rv in our assay panel as this species, but not other species of this genus, has recently been reported to be susceptible to carbamidocyclophanes [6]. The bacterial panel was completed by different *E. coli* strains as well as *K. pneumoniae* and *P. aeruginosa* (Table 3 and Table 4)*.*

Most of the [7.7]paracyclophanes showed remarkable potency against *Staphylococcus* species (MICs between 0.1 and 1.5 µM) that were generally independent from the degree of halogenation as well as the nature of the halogen atom (Table 3). Moreover, the activity against MRSA and VISA strains equals that against MSSA strain Newman, corroborating our previous results that the target of the [7.7]paracyclophanes is different to that of methicillin and vancomycin [7]. Except for cylindrocyclophane A (**25**), non-carbamoylated or diacetylated [7.7]paracyclophanes (e.g., cylindrocyclophane A_1_ (**21**) and D (**27**)) tended to be slightly less potent against MRSA, especially against strains N315 and Mu50 (MICs in the range 0.8‒12.9 µM) than the other derivatives, as it has been reported previously for MRSA strain 1 [7].

The [7.7]paracyclophane activity data against selected *Enterococcus* and *Streptococcus* strains generally showed a similar trend as described above for staphylococci. However, on average, MIC values were increased by a factor of 2 and 28 against streptococci and enterococci, respectively. In addition to the latter testing, monocarbamoylated compounds, except brominated carbamidocyclophane R (**6**), revealed on average 3.5-fold lower MIC values than their dicarbamoylated congeners. In particular, carbamidocyclophane Q (**5**) showed decreased activity (MICs in the range 16.2‒32.4 µM), which means both an up to 13-fold loss in the anti-*Enterococcus* activity compared to the most potent derivative, brominated carbamidocyclophane S (**7**) (MIC = 2.5 µM), and an up to 324-fold reduction with regard to the strong anti-MRSA activity of **7**. On the other hand, diacetylated compound **27** revealed the lowest antimicrobial activity against *Streptococcus* spp. (MIC = 3.0‒12.0 µM), and was up to 60-fold less effective than the most potent compounds, such as its monoacetylated congener cylindrocyclophane B (**26**).

The *M. tuberculosis* bioactivity assay revealed most of the [7.7]paracyclophanes tested to be highly active with MIC values in the range 1.1‒6.8 µM. Based on the prerequisite to identify a compound as relevantly active revealing an MIC lower than 8 µg/mL and 10 µM [6], respectively, only carbamidocyclophanes M (**1**) and R (**6**) slightly exceeded the threshold. Furthermore, we identified the non-carbamoylated cylindrocyclophanes A_1_ (**21**), A_2_ (**22**) and A_4_ (**24**) to be non-active against *M. tuberculosis* H37Rv, which was also confirmed for dicarbamoylated and tetrachlorinated carbamidocyclophane A (**14**) [6]. Contrary to our expectations, the tetrabrominated analogue **5** was found to be active revealing a MIC value of 2.0‒4.1 µM. This result means an up to 40-fold increase in bioactivity by substituting bromine for all chlorine atoms. As discussed above, notable increased potency of brominated compounds compared to their chlorinated analogues is rarely observed. Especially for the substitution event as seen in terms of **5** and **14**, it has been reported even less frequently, such as for duocarmycins [53]. To the best of our knowledge, this significant difference in the antibiotic activity is unprecedented. In addition, we confirmed the identity and stability of **5** using subsequent HPLC-UV-MS analysis of the tested sample (Figure S4). In contrast to previous results, we determined carbamidocyclophane B (**13**) (MIC = 1.3‒1.9 µM) and C (**12**) (MIC = 2.7‒5.4 µM) as active in this study. With reference to our data, the presence of a hydroxy group at C-14 seems not to play a significant role for the antimycobacterial activity of [7.7]paracyclophanes as previously stated [6] but is rather subject to a complex contribution of various substituents.

The linear cylindrocyclophane-related cylindrofridins **28**‒**30** also showed antimicrobial activity in the low µM range against *S. aureus* Newman (MICs between 3.0 and 11.3 µM) and *S. pneumoniae* (MICs in the range 2.8‒12.1 µM; only DSM-strains). Furthermore, in contrast to diacetylated cylindrofridin C (**30**), monoacetylated congeners A (**28)** and B (**29)** were also active against MRSA strains N315 and Mu50 (MICs between 6.0 and 21.6 µM). Notably for **29**, this is the first report of an anti-MRSA activity, since **29** and **30** were not active against MRSA strain 1 and *S. pneumoniae* strain 7 [38]. Furthermore, no activity against *M. tuberculosis* and only a very low activity against *Enterococcus* was observed for **28** and **29** (MIC in the range 43.1‒96.5 µM).

However, MIC values of cylindrofridins were generally reduced by a factor of 6‒8 against streptococci and of 10‒28 against staphylococci compared to [7.7]paracyclophanes. This trend was even more pronounced for cytotoxicity against HaCaT cells, revealing an up to 21‒85 fold lower toxicity of these alkylresorcinols compared to the [7.7]paracyclophanes (Table 4). Furthermore, cytotoxicity of brominated carbamidocyclophanes is similar to that of the chlorinated analogues. Non-carbamoylated derivatives are slightly less cytotoxic. These results highlight once again that cytotoxicity of [7.7]paracyclophanes is primarily based on the macrocyclic core structure [54].

All compounds tested showed no activity against *E. coli* strains, including an efflux-deficient (TolC) strain, *K. pneumoniae*, and *P. aeruginosa*. However, the addition of polymyxin B nonapeptide (PMBN) for permeabilization of the outer bilayer membrane of the TolC-deficient *E. coli* cell wall, and thus facilitating compound penetration, resulted in the determination of strong antimicrobial activities of most [7.7]paracyclophanes. Except for **5** and **21**, especially carbamoylated compounds but also non-acetylated cylindrocyclophanes revealed pronounced bioactivity (MICs in the range 0.7−6.0 µM) similar to obtained MIC values against Gram-positive bacteria. Interestingly, acetylated cylindrocyclophanes **26** and **27** (MICs of 3.2‒6.4 and 95.7 µM) as well as cylindrofridins **28** and **29** (MICs of 10.8 and 12.1‒48.2 µM) tended to be less potent, and diacetylated cylindrofridin **30** was not active in the concentration range tested. These data may suggest that hindered compound uptake mainly—and not compound efflux—leads to the lack of activity against Gram-negative species. 

**Table 3:** Biological activity of compounds **1**‒**30** against selected drug-susceptible and drug-resistant Gram-positive bacteria ^a^.

#	MIC (µM) ^b^									
	*E. faecium*	*E. faecium* DSM-17050 (VREF)	*M. tuberculosis* ATCC 25618 (H37Rv)	*S. aureus* Newman (MSSA)	*S. aureus* N315 (MRSA)	*S.* *aureus* 1 (MRSA) ^c^	*S. aureus* Mu50 (MRSA/VISA)	*S. pneumoniae*	*S. pneumoniae*	*S. pneumoniae* DSM-11865 (PRSP)
	DSM-20477							7 (ATCC 49619) ^c^	DSM-20566	
		
**1**	10.7	2.7‒10.7	10.7‒16.0	1.3	0.3‒0.7	0.1	0.7	0.3	1.3	2.7
**2**	9.7	4.8‒9.7	2.4‒6.0	0.3	0.3‒0.6	n.t.	0.3‒0.6	n.t.	1.2	0.3‒0.6
**3**	9.7	9.7	1.2‒1.8	0.2‒0.3	0.2	0.8	0.2‒0.3	0.2	0.6	0.3‒0.6
**4**	8.8	4.4‒8.8	0.6‒1.7	0.6	0.3‒0.6	0.1	0.3	0.2	1.1	0.6
**5**	32.4	16.2‒32.4	2.0‒4.1	0.3	0.3	0.2	0.1	0.2	1.0	0.5‒2.0
**6**	n.t.	11.3‒22.6	5.7‒11.3	n.t.	0.7	n.t.	n.t.	n.t.	n.t.	1.4
**7**	2.5	2.5	0.6‒1.9	0.2	0.1‒0.2	0.1	0.2	0.3	0.3	0.3
**8**	4.6	4.6	2.3‒5.8	0.3	0.1‒0.3	n.t.	0.3	n.t.	0.6‒1.2	0.6
**9**	8.5	4.2	0.6‒1.9	0.1‒0.3	0.1	0.2	0.5	0.2	0.3‒0.5	0.3
**10**	5.7	5.7	2.8‒4.3	0.2‒0.4	0.2	0.1	0.2	0.3	0.7‒1.4	0.4‒0.7
**11**	n.t.	10.8	2.7‒5.4	n.t.	0.4	0.1	n.t.	0.3	n.t.	0.3
**12**	5.4	2.7‒5.4	2.7‒5.4	0.2‒0.3	0.2	0.1	0.2	0.3	0.3‒0.7	0.3
**13**	5.2	5.2	1.3‒1.9	0.2‒0.3	0.2	0.1	0.2	0.3	0.3‒0.6	0.2‒0.3
**14**	4.9	4.9	39.6‒79.1	0.3	0.2	0.1	0.2	0.3	0.3‒0.6	0.3‒0.6
**15**	nt	6.0	3.0‒4.5	n.t.	0.4	0.1	n.t.	0.3	n.t.	0.8
**16**	2.9	2.9	2.2‒2.9	0.2‒0.4	0.1‒0.2	0.1	0.2	0.3	0.4	0.7
**17**	2.7	2.7	2.7‒6.8	0.2‒0.3	0.1‒0.2	0.1	0.2	0.3	0.3	0.3
**18**	2.6‒5.2	2.6	1.3‒2.0	0.2	0.1	0.1	0.2	0.3	0.3	0.3
**19**	6.0‒11.9	11.9	3.0‒4.5	0.2	0.2	0.1	0.2	0.3	0.7	0.4
**20**	n.t.	3.2	2.4‒3.2	n.t.	0.2‒0.4	0.1	n.t.	0.3	n.t.	0.4
**21**	n.t.	12.9	>12.9	1.6‒3.2	0.8	1.0	12.9	2.1	n.t.	3.2
**22**	n.t.	n.t.	>12.2	n.t.	n.t.	1.0	n.t.	2.0	n.t.	n.t.
**23**	n.t.	5.8‒11.6	2.9‒5.8	0.7	0.7	0.5	1.5	0.9	n.t.	n.t.
**24**	n.t.	n.t.	>11.1	n.t.	n.t.	0.5	n.t.	0.9	n.t.	n.t.
**25**	6.8	3.4	3.4‒6.8	0.2	0.2	0.5	0.2	1.0	0.4	0.4
**26**	6.4	3.2	0.8‒1.6	0.2	0.2	0.1	0.2	0.3	0.8	0.2
**27**	12.0	3.0	1.5‒3.0	1.6‒3.2	0.7‒3.0	0.9	3.0‒12.0	2.4	3.0‒12.0	6.0‒12.0
**28**	43.1	43.1	>43.1	10.8	10.8‒21.6	8.6	10.8‒21.6	16.8	10.8	10.8
**29**	96.5	96.5	>24.1	3.0‒6.0	6.0	>75.4	6.0‒12.1	>75.4	3.0‒12.1	3.0
**30**	>90.7	>90.7	>22.7	5.7‒11.3	>90.7	>70.9	>90.7	>70.9	5.7	2.8‒5.7
**POS**	1.4 ^d^	>44 ^d^	0.05‒0.07 ^e^ 0.4‒0.7 ^f^ 0.2‒0.6 ^g^ 0.2‒1.0 ^h^	0.4 ^d^	0.7 ^d^	1.4 ^d^ 3.8 ^i^	11.0 ^d^	1.4 ^d^ 3.8 ^i^	<0.09 ^j^	>183 ^j^

^a^ Abbreviations: see footnote ‘a’ of Table 2; ^b^ For unprocessed data in µg/mL, see Table S10; ^c^ Equivalent data for **10**‒**30** have previously been reported; For further details, see Preisitsch *et al.* [7] (**10**‒**25**) and Preisitsch *et al.* [38] (**26**‒**30**); ^d^ vancomycin; ^e^ delamanid; ^f^ pretomanid (formerly known as PA-824); ^g^ isoniazid; ^h^ rifampicin; ^i^ fusidic acid; ^j^ ampicillin.

**Table 4:** Biological activity of compounds **1**‒**30** against selected drug-susceptible and drug-resistant Gram-negative bacteria and HaCaT cells ^a^.

#	MIC (µM)					IC_50_ (µM)
	*E. coli*	*E. coli*	*E. coli* TolC-deficient + PMBN	*K. pneumoniae* 18 (KRKP) ^c^	*P. aeruginosa* 22 (MDR) ^c^	HaCaT Cells ^c^
	13 ^c^	TolC-deficient				
	
**1**	>66.7	>85.4	2.7	>66.7	>66.7	3.9
**2**	n.t.	>77.2	1.2‒2.4	n.t.	n.t.	n.t.
**3**	>60.3	>77.2	2.4	>60.3	>60.3	2.5
**4**	>55.1	>70.5	1.1	>55.1	>55.1	3.9
**5**	>50.7	>64.9	8.1‒16.2	>50.7	>50.7	7.5
**6**	n.t.	n.t.	n.t.	n.t.	n.t.	n.t.
**7**	>63.6	>81.5	1.3	>63.6	>63.6	3.1
**8**	n.t.	>74.0	1.2	n.t.	n.t.	n.t.
**9**	>53.0	>67.8	1.1	>53.0	>53.0	7.9
**10**	>70.9	>90.7	1.4‒2.8	>70.9	>70.9	5.6
**11**	>67.6	>86.5	n.t.	>67.6	>67.6	3.0
**12**	>67.6	>86.5	1.4	>67.6	>67.6	2.8
**13**	>64.6	>82.7	1.3	>64.6	>64.6	4.4
**14**	>61.8	>79.1	1.2‒2.5	>61.8	>61.8	4.8
**15**	>75.1	>96.6	n.t.	>75.1	>75.1	5.8
**16**	>71.8	>91.9	0.7‒1.4	>71.8	>71.8	3.8
**17**	>68.4	>87.5	0.7	>68.4	>68.4	4.6
**18**	>65.3	>83.6	0.7‒1.3	>65.3	>65.3	4.7
**19**	>74.5	>95.4	1.5‒6.0	>74.5	>74.5	3.7
**20**	>79.6	>101.9	n.t.	>79.6	>79.6	7.6
**21**	>80.7	>103.3	6.5‒12.9	>80.7	>80.7	11.3
**22**	>76.5	n.t.	n.t.	>76.5	>76.5	11.5
**23**	>72.7	>93.0	2.9	>72.7	>72.7	8.6
**24**	>69.2	n.t.	n.t.	>69.2	>69.2	9.3
**25**	>85.5	>109.4	1.7‒3.4	>85.5	>85.5	5.0
**26**	>79.8	>102.1	3.2‒6.4	>79.8	>79.8	2.9
**27**	>74.8	>95.7	95.7	>74.8	>74.8	10.9
**28**	>134.8	>172.6	10.8	>134.8	>134.8	100.4
**29**	>75.4	>96.5	12.1‒48.2	>75.4	>75.4	24.9
**30**	>70.9	>90.7	>90.7	>70.9	>70.9	24.8
**POS**	0.019 ^d^ 0.015 ^e^ 0.017 ^f^ 43.1 ^g^	0.009 ^d^	0.009 ^d^	3.8 ^d^ 1.5 ^e^ 3.5 ^f^	0.069 ^f^ 172.4 ^g^	3.9 ^h^

^a^ Abbreviations: PMBN = polymyxin B nonapeptide, see also footnote ‘a’ of Table 2; ^b^ For unprocessed data in µg/mL, see Table S10; ^c^ Equivalent data for **10**‒**30** have previously been reported; For further details, see Preisitsch *et al.* [7] (**10**‒**25**) and Preisitsch *et al.* [38] (**26**‒**30**); ^d^ ciprofloxacin; ^e^ moxifloxacin; ^f^ levofloxacin; ^g^ vancomycin; ^h^ mitoxantrone.

## 3. Experimental Section

### 3.1. General Experimental Procedures

Optical rotations were determined on a Jasco P-2000 polarimeter. UV spectra were measured on a Shimadzu UVmini-1240 UV-vis spectrophotometer in the wavelength range from 190 to 400 nm. ECD spectra were recorded on a Jasco J-810 spectropolarimeter. Attenuated total reflexion-infrared (ATR-IR) spectra were recorded using a Thermo Scientific Nicolet IR 200 Fourier transform infrared (FT-IR) spectrometer. HPLC-UV-MS analysis was conducted on a Shimadzu LC-20A Prominence liquid chromatography system with a SPD-M20A diode array detector (DAD) coupled either to a Shimadzu LCMS-8030 triple quadrupole (QqQ) mass spectrometer or to a Shimadzu Ion Trap-Time of Flight (IT-TOF) mass spectrometer equipped with an electrospray ionization (ESI) source. Semi-preparative HPLC was performed on a Shimadzu HPLC system including a SPD-M10Avp DAD. More detailed information of above-mentioned general experimental conditions is described by Preisitsch *et al.* [7]. Samples for NMR spectroscopy were dissolved in 600 µL MeOH-*d*_4_ to yield concentrations of 3.3 mM (**1**), 1.8 mM (**2**), 4.6 mM (**3**), 3.1 mM (**4**), 5.3 mM (**5**), 0.7 mM (**6**), 2.3 mM (**7**), 1.7 mM (**8**), and 3.5 mM (**9**). NMR spectra were recorded at 600 MHz (^1^H frequency) on Bruker AV-III spectrometers using either a cryogenically cooled 5 mm TCI-triple resonance probe or a room-temperature 5 mm QXI probe, both equipped with one-axis self-shielded gradients at 300 K. For the samples with the highest concentrations a full set of homonuclear and heteronuclear two-dimensional spectra was recorded first. Homonuclear spectra (DQF-COSY [55], TOCSY [56,57]) were recorded using 2048 × 512 complex data points using 8 scans. Several mixing times were used for the TOCSYs (10-60 msec). Heteronuclear two-dimensional ^13^C-HMQC [58], ^13^C-DEPT-HMQC [59], ^13^C-HMQC-TOCSY [60], and ^13^C-HMQC-COSY [60] spectra were recorded using 512 × 512 complex data points using 8 scans; in the TOCSY the mixing time was 20 and 30 msec. All the above heteronuclear spectra were recorded using a BIRD pulse for suppression of protons bound to ^12^C [61]. Gradient-enhanced-^13^C-HMBC [62] spectra were recorded with 2048 × 1024 complex data points using 72 scans. For the other samples a reduced set of spectra was recorded (DQF-COSY, ^13^C-DEPT-HMQC, ^13^C-HMQC-TOCSY and ^13^C-HMBC), only the number of scans was adjusted for the lower concentrations. The three compounds that contained only one carbamoyl moiety and less than 4 bromines required an additional TOCSY with CHIRP-z-Filter [63] to clarify on which side of the molecule the carbamoyl was attached. The TOCSY was recorded with a mixing time of 150 msec and 96 scans, using the same 2048 × 512 complex data points as above. Spectra were referenced indirectly. ^13^C and ^1^H chemical shifts were extracted from the two-dimensional ^13^C-spectra.

### 3.2. Biological Material, Culture Conditions and Sample Processing Procedures

#### 3.2.1. *Nostoc* sp. CAVN2

The unialgal cyanobacterium *Nostoc* sp. strain CAVN2 is part of the culture collection of the Institute of Pharmacy, Ernst-Moritz-Arndt-University, Greifswald. Taxonomic identification has been described previously [7].

#### 3.2.2. Analytical Investigation on Halogen Atom Incorporation and Compound Quantification

To investigate the biosynthetic incorporation of different halide ions, 5-mL aliquots (corresponding to 1.9 ± 0.5 mg biomass) of *Nostoc* sp. CAVN2 stock culture were used to inoculate 100-mL Erlenmeyer flasks containing 45 mL Z½ medium (for detailed composition, see Table S1). Additionally, cultures were grown either in the presence of KBr, KCl, KF, or KI to give a final concentration of 0.01, 0.1, 1.0, and 10.0 g/L. The flasks were shaken (100 rpm) on a New Brunswick Innova 2100 open-air laboratory shaker at 25 ± 1 °C and exposed to a continuous illumination of 18 ± 1 µmol photons m^‒2^·s^‒1^ (Osram fluorescent lamp Lumilux 36 W/840) for 20, 25, and 30 days. All cultivations were performed twice with two technical replicates per treatment. The biomass was harvested by centrifugation (5300 *g*, 5 min at 20 °C). The resulting cell pellet was washed with 15 mL of distilled H_2_O, centrifuged again, freeze-dried, and stored at ‒20 °C until use. Biomass samples of 10.0 mg were subjected to the cyclophane-directed extraction and enrichment procedure using the biphasic solvent mixture consisting of *n*-heptane, EtOAc, EtOH and H_2_O (5:2:5:2, v/v/v/v) as described previously [38]. This time, however, the volumes of the lower and upper phase were increased to 5 mL each. Extraction was performed by stirring (750 rpm) for 24 h. After centrifugation (3300 *g*, 5 min at 20 °C), 2.5 mL of the lower phase were reduced to dryness by centrifugal evaporation, dissolved again in 80% MeOH (HPLC grade) to a final concentration of 15 mg of treated dry biomass per mL MeOH. The solution was filtrated through a 0.2 µm PTFE syringe filter, and 20 µL sample were subjected to analytical reversed-phase HPLC-DAD analysis using a Phenomenex Luna PFP (2) column (250 × 4.6 mm, 5 µm, 100 Å) and a binary gradient of MeOH in deionized H_2_O with a flow rate of 0.8 mL/min from 60% to 85% MeOH in 32 min at 25 °C. Determination of the total [7.7]paracyclophane amount was based on peak area integration of compounds eluting in the range 16.0‒39.5 min as well as revealing carbamidocyclophane-like UV spectra [7], and data were calculated in relation to control data, which represent samples cultivated without halogen supplement. The average value of control samples was defined as 100% total [7.7]paracyclophane amount. HPLC separation of 5-µL-samples for subsequent DAD-MS analysis was performed using a Phenomenex Kinetex column (100 × 4.6 mm, 2.6 µm, 100 Å) and the binary gradient as already described for HPLC-DAD analysis. Based on corresponding HR-ESI-MS/MS data, structural proposals of unknown compounds and identification of known [7.7]paracyclophanes were made for selected peaks of the corresponding UV chromatogram as described previously [7].

For further comparison of the influence of chloride and bromide on cyanobacterial growth and carbamidocyclophane biosynthesis in *Nostoc* sp. CAVN2, samples were cultured in a Sartorius CERTOMAT BS-1 shaking incubator either in the presence of 0.01 M KBr or KCl. After inoculation of 2.2 ± 0.2 mg biomass, the flasks were shaken at 80 rpm at 25 ± 1 °C and exposed to a continuous illumination of 15 ± 1 µmol photons m^‒2^·s^‒1^ (two Sylvania fluorescent lamps GROLUX T8 F18W) over 36 days. The biomass was harvested and freeze-dried according to aforementioned procedure. Cultivation was done three times with two technical replicates per treatment. Growth was determined by measuring the dry weights. The deduced growth curves were the basis for the calculation of maximum specific growth rate (*µ*_max_) [37]. For compound analysis, a 10.0 mg biomass aliquot was subjected to the above-mentioned one-step biomass extraction and [7.7]paracyclophane enrichment procedure [38]. Biomass samples were extracted according to the protocol for 24 h. After sample preparation [38], compounds were separated using a Phenomenex Kinetex HPLC column (250 × 4.6 mm, 5 µm, 100 Å) and a gradient of MeOH in deionized H_2_O with a flow rate of 1.0 mL/min from 60% to 85% MeOH in 32 min at 40 °C. Carbamidocyclophane quantification was performed by UV analysis at 226 nm wavelength. Routinely, samples were subsequently analyzed by QqQ-MS. For non-halogenated and chlorinated [7.7]paracyclophanes, carbamidocyclophane A (**14**) was utilized as external reference substance, and carbamidocyclophane Q (**5**) was utilized as reference standard for brominated analogues, respectively.

CAVN2 cultures, containing 0.01 M KBr, 0.01 KCl or both KBr and KCl in the composition of 0.01:0.001, 0.01:0.01, or 0.001:0.01 M, were grown with a shaking speed of 100 rpm on a New Brunswick Innova 2100 open-air laboratory shaker at 25 ± 1 °C. The culture were exposed to a continuous illumination of 21 ± 1 µmol photons m^‒2^·s^‒1^ (Sylvania fluorescent lamp GROLUX T5 F39W) for 20 days. Cultivation was performed three times with two technical replicates per treatment. Inoculation, sample processing and compound quantification was carried out as described in the previous paragraph.

#### 3.2.3. Large Scale Cultivation in KBr-Enriched Medium

Halogen-depleted stock culture of *Nostoc* sp. CAVN2 was cultured in a 40 L bioreactor [64] containing 36 L Z½ medium under similar conditions as previously described for biomass production to isolate chlorinated carbamidocyclophanes and cylindrocyclophanes [7]. Two weeks after inoculation, KBr was added to reach a final concentration of 0.1% in the medium, and cultivation was continued for four weeks. The biomass was harvested by centrifugation, freeze-dried and stored at ‒20 °C until use. The yield of lyophilized biomass was 224 mg/L.

#### 3.2.4. Compound Isolation

For initial evaluation of bioactivity, 0.5 g of aforementioned *Nostoc* sp. CAVN2 biomass was extracted by stirring (stirring bar 25 × 8 mm, 300 rpm) using 50 mL MeOH. After centrifugation (3300 *g*, 10 min at 4 °C), the supernatant was removed from the biomass pellet. Extraction was repeated twice under described conditions, and supernatants were combined and evaporated to yield 56.1 mg MeOH extract. For compound isolation, two separate portions of 2.0 g were extracted by stirring (stirring bar 40 × 8 mm; 750 rpm) over 24 hours in recently described biphasic solvent system [38] consisting of 100 mL of the upper and the lower phase. After centrifugation (3300 *g*, 10 min at 4 °C), the supernatants were removed from the biomass residues. Extraction was repeated twice under described conditions. The supernatants were combined and filtrated through Whatman folded filter papers 595 1/2 (Ø 185 mm). The lower solvent phase was evaporated. The residue was dissolved in 80 or 100% MeOH, filtered through a 0.2 µm PVDF syringe filter and re-evaporated to yield 388.5 mg. This extract was subjected to semi-preparative reversed-phase HPLC using a Phenomenex Synergi Polar RP column (250 × 10.0 mm, 4 µm) and a binary gradient of MeOH in deionized water from 67% to 88% MeOH in 32 min with a flow rate of 3.5 mL/min at 15 °C. Multiple rounds of isolation yielded eight fractions, namely: PBr1 (9.5 mg), PBr2 (4.2 mg), PBr4 (9.1 mg), PBr6 (33.5 mg), PBr7 (3.4 mg), PBr8 (27.5 mg), PBr10 (73.2 mg), and PBr11 (55.8 mg). For final isolation of compounds, fractions were subjected to semi-preparative reversed-phase HPLC, this time using a Phenomenex Luna PFP(2) column (250 × 10.0 mm, 5 µm, 100 Å) and a flow rate of 3.5 mL/min at 30 °C. A portion of PBr4 (8.9 mg) yielded **1** (2.9 mg, 0.07% of dry biomass) and **6** (0.3 mg, 0.01% of dry biomass) by using isocratic conditions of 64% MeOH in H_2_O. Several rounds of isolation, using a binary MeOH-H_2_O gradient from 63% to 84% MeOH in 32 min, yielded **2** (1.3 mg, 0.03% of dry biomass), **3** (20.0 mg, 0.50% of dry biomass), and **7** (2.2 mg, 0.06% of dry biomass) from PBr6 (33.2 mg). Isocratic working conditions of 65.5% MeOH in H_2_O resulted in the isolation of **4** (6.8 mg, 0.17% of dry biomass) and **8** (1.2 mg, 0.03% of dry biomass) from PBr8 (27.1 mg). A portion of PBr10 (66.6 mg) yielded **5** (51.1 mg, 1.4% of dry biomass) and **9** (3.6 mg, 0.10% of dry biomass). For tracking and purity control of brominated compounds, analytical HPLC-DAD-QqQ-MS analysis of PBr1‒PBr11 and isolated compounds was performed by using a Phenomenex Kinetex PFP column (100 × 4.6 mm, 2.6 µm, 100 Å) and a gradient of MeOH in deionized H_2_O with a flow rate of 0.8 mL/min from 60% to 80.3% MeOH in 32 min at 40 °C.

#### 3.2.5. Physical and Spectroscopic Data of **1**‒**9**

Carbamidocyclophane M (**1**): white, amorphous powder; ${{\left[\alpha \right]}^{20}}_{D}$ +20.0 (*c* 0.2, MeOH); UV (MeOH) λ_max_ (log ε)221 (4.19), 227 (sh) (4.14), 275.5 (3.30) nm; ECD (*c* 0.0022; MeOH) λ_max_ (Δε) 202.5 (23.21), 211 (7.48), 224 (1.19), 238 (1.03), 258.5 (−0.39), 273 (−0.85) nm; ATR-IR (film) ν_max_ 3378 (br), 2928, 2856, 1698, 1590, 1432, 1374, 1334, 1044, 1018, 832 cm^−1^; ^1^H and ^13^C NMR data, see Table S3; HRESIMS *m/z* 747.3201 [M − H]^−^ (calcd for C_38_H_56_^79^BrN_2_O_8_, 747.3226; Δ = 3.4 ppm).

Carbamidocyclophane N (**2**): White, amorphous powder; ${{\left[\alpha \right]}^{20}}_{D}$ +10.0 (*c* 0.2, MeOH); UV (MeOH) λ_max_ (log ε) 216 (4.31), 226 (sh) (4.10), 275 (3.15), 282.5 (3.12) nm; ECD (*c* 0.0025; MeOH) λ_max_ (Δε) 209.5 (5.03), 220.5 (0.35), 228 (0.07), 237 (0.89), 280.5 (−0.61) nm; ATR-IR (film) ν_max_ 3366, 2927, 2855, 1697, 1589, 1431, 1372, 1338, 1042, 1016, 832, 648 cm^−1^; ^1^H and ^13^C NMR data, see Table S3; HRESIMS *m/z* 825.2331 [M − H]^−^ (calcd for C_38_H_55_^79^Br_2_N_2_O_8_, 825.2331; Δ = 0.0 ppm).

Carbamidocyclophane O (**3**): White, amorphous powder; ${{\left[\alpha \right]}^{20}}_{D}$ +10.0 (*c* 0.2, MeOH) UV (MeOH) λ_max_ (log ε) 218 (4.41), 226.5 (sh) (4.28), 275 (3.34), 283 (3.31) nm; ECD (*c* 0.0025; MeOH) λ_max_ (Δε) 202 (−2.18), 204.5 (−6.79), 210.5 (6.23), 220 (0.86), 231.5 (1.80), 278 (−1.21) nm; ATR-IR (film) ν_max_ 3488, 3361 (br), 2927, 2856, 1699, 1615, 1588, 1431, 1389, 1374, 1334, 1044, 1016, 830, 778, 668, 649 cm^−1^; ^1^H and ^13^C NMR data, see Table S3; HRESIMS *m/z* 825.2331 [M − H]^−^ (calcd for C_38_H_55_^79^Br_2_N_2_O_8_, 825.2331; Δ = 0.0 ppm).

Carbamidocyclophane P (**4**): White, amorphous powder; ${{\left[\alpha \right]}^{20}}_{D}$ +5.0 (*c* 0.2, MeOH); UV (MeOH) λ_max_ (log ε) 215.5 (4.45), 227 (sh) (4.23), 275 (3.39), 283.5 (3.36) nm; ECD (*c* 0.0027; MeOH) λ_max_ (Δε) 210.5 (8.20), 218.5 (−0.68), 234 (1.79), 254.5 (0.03), 271 (−0.29), 282 (−0.38) nm; ATR-IR (film) ν_max_ 3490, 3385 (br), 2922, 2856, 1696, 1617, 1587, 1431, 1374, 1333, 1042, 1002, 831, 778, 669, 649cm^−1^; ^1^H and ^13^C NMR data, see Table S3; HRESIMS *m/z* 903.1455 [M − H]^−^ (calcd for C_38_H_54_^79^Br_3_N_2_O_8_, 903.1436; Δ = 2.1 ppm).

Carbamidocyclophane Q (**5**): White, amorphous powder; ${{\left[\alpha \right]}^{20}}_{D}$ ±0.0 (*c* 0.2, MeOH); UV (MeOH) λ_max_ (log ε) 215.5 (4.49), 227 (sh) (4.26), 275 (3.43), 282 (3.41) nm; ECD (*c* 0.0030; MeOH) λ_max_ (Δε) 202 (−5.05), 212.5 (2.50), 220.5 (0.76), 233.5 (1.67), 256 (−0.19), 282 (−0.68) nm; ATR-IR (film) ν_max_ 3498, 3366 (br), 2930, 2854, 1699, 1616, 1588, 1431, 1389, 1374, 1332, 1042, 1014, 831, 779, 668, 559 cm^−1^; ^1^H and ^13^C NMR data, see Table S3; HRESIMS *m/z* 981.0547 [M − H]^−^ (calcd for C_38_H_53_^79^Br_4_N_2_O_8_, 981.0541; Δ = 0.6 ppm).

Carbamidocyclophane R (**6**): White, amorphous powder; ${{\left[\alpha \right]}^{20}}_{D}$ +25.0 (*c* 0.2, MeOH); UV (MeOH) λ_max_ (log ε) 224 (4.22), 275 (3.47) nm; ECD (*c* 0.0041; MeOH) λ_max_ (Δε) 206 (0.11), 210.5 (1.34), 219.5 (−0.61), 227 (−0.15), 230 (−0.18), 238 (0.13), 252.5 (−0.20), 280 (−0.37) nm; ATR-IR (film) ν_max_ 3358 (br), 2925, 2854, 1699, 1588, 1430, 1365, 1043, 1018, 833, 646, 619 cm^−1^; ^1^H and ^13^C NMR data, see Table S4; HRESIMS *m/z* 704.3170 [M − H]^−^ (calcd for C_37_H_55_^79^BrNO_7_, 704.3167; Δ = 0.4 ppm).

Carbamidocyclophane S (**7**): White, amorphous powder; ${{\left[\alpha \right]}^{20}}_{D}$ ‒20.0 (*c* 0.2, MeOH); UV (MeOH) λ_max_ (log ε) 216.5 (4.44), 226 (sh) (4.27), 275 (3.42), 282 (3.38) nm; ECD (*c* 0.0024; MeOH) λ_max_ (Δε) 206 (−3.19), 211.5 (0.09), 222 (−2.47), 266 (−0.29), 273 (−0.53) nm; ATR-IR (film) ν_max_ 3346 (br), 2918, 2850, 1699, 1589, 1430, 1378, 1334, 1017, 832, 553, 536, 453 cm^−1^; ^1^H and ^13^C NMR data, see Table S4; HRESIMS *m/z* 782.2259 [M − H]^−^ (calcd for C_37_H_54_^79^Br_2_NO_7_, 782.2272; Δ = 1.7 ppm).

Carbamidocyclophane T (**8**): White, amorphous powder; ${{\left[\alpha \right]}^{20}}_{D}$ ‒10.0 (*c* 0.2, MeOH); UV (MeOH) λ_max_ (log ε) 215.5 (4.41), 226 (sh) (4.17), 274 (3.34), 282 (3.31) nm; ECD (*c* 0.0026; MeOH) λ_max_ (Δε) 208.5 (4.64), 217 (−0.93), 227.5 (−0.15), 233 (−0.66), 247.5 (−0.10), 277.5 (−0.44) nm; ATR-IR (film) ν_max_ 3398 (br), 2916, 2849, 1663, 1579, 1403, 1356, 1118, 1039, 1017, 835, 764, 726, 533 cm^−1^; ^1^H and ^13^C NMR data, see Table S4; HRESIMS *m/z* 860.1360 [M − H]^−^ (calcd for C_37_H_53_^79^Br_3_NO_7_, 860.1378; Δ = 2.1 ppm).

Carbamidocyclophane U (**9**): White, amorphous powder; ${{\left[\alpha \right]}^{20}}_{D}$ ‒20.0 (*c* 0.2, MeOH); UV (MeOH) λ_max_ (log ε) 215.5 (4.52), 226 (sh) (4.32), 274.5 (3.56), 282 (3.52) nm; ECD (*c* 0.0028; MeOH) λ_max_ (Δε) 203.5 (−8.85), 208.5 (2.65), 217 (−3.84), 250.5 (−0.18), 279 (−1.11) nm; ATR-IR (film) ν_max_ 3345 (br), 2925, 2855, 1702, 1585, 1430, 1364, 1336, 1045, 1014, 831, 669, 620, 567 cm^−1^; ^1^H and ^13^C NMR data, see Table S4; HRESIMS *m/z* 938.0486 [M − H]^−^ (calcd for C_37_H_52_^79^Br_4_NO_7_, 938.0483; Δ = 0.3 ppm).

### 3.3. Carbamidocyclophane Gene Cluster Identification

#### 3.3.1. Genomic DNA Isolation and Whole Genome Shotgun Sequencing

Genomic DNA of axenic *Nostoc* sp. CAVN2 was isolated according to the methodology described by Wu *et al.* [65]. The extracted DNA was used to generate 454-shotgun libraries according to the manufacturer’s protocols. The libraries were sequenced using a 454 GS-FLX system (Titanium GS70 chemistry; Roche Life Sciences, Mannheim, Germany). The assembly was performed *de novo* with the Roche Newbler assembly software 2.1.

#### 3.3.2. Gene Cluster Identification and Gap-Closure

A local BLAST+ [66,67] nucleotide database was created from the draft genome of *Nostoc* sp. CAVN2. Subsequently, a MegaBLAST search of the published cylindrocyclophane biosynthetic gene cluster of *C. licheniforme* UTEX ‘B 2014’ (acc. no. JX477167) against this database was performed. Matching contigs were mapped to the cylindrocyclophane gene cluster using tools included in Geneious version 6.1.8 (http://www.geneious.com, [68]). Oligonucleotide primers (Table S9) were derived from the respective contig ends and standard PCR technique (Phusion High-Fidelity DNA polymerase; Life Technologies, Darmstadt, Germany) was used to amplify regions spanning the gap between contigs. PCR products were purified using the High Pure PCR Product Purification Kit (Roche Life Sciences, Mannheim, Germany) and sequenced by Eurofins Genomics (Ebersberg, Germany). Assembly of contigs and sequenced amplicons was done in Geneious.

#### 3.3.3. Frameshift Refutation and Annotation

A preliminary functional assessment employed MetaGene [69] for open reading frame (ORF) prediction, the GenDB system [70] for protein similarity searches and MicHanThi [71] for automatic annotation. The existence of putative frameshifts was refuted by Sanger dideoxy sequencing [44] after amplification of questionable regions using standard PCR technique (Phusion High-Fidelity DNA polymerase; New England Biolabs, Ipswich, MA, and OptiTaq DNA Polymerase; Roboklon, Berlin, Germany) with adapted MgCl_2_ concentration (5 mM) and additional DMSO (3%). Oligonucleotide primers (Table S9) were designed using Primer3 [72,73]. The final ORF prediction on the curated gene cluster was done using Prodigal version 2.6.2 [74]. Annotations were added manually considering BlastP and InterProScan [75,76] search results.

#### 3.3.4. Gene Cluster Comparison

The biosynthetic gene cluster of *C. stagnale* PCC 7417 was identified along the lines of the *Nostoc* sp. CAVN2 cluster by use of a local BLAST+ database. Bl2seq (MegaBLAST, E-value 1e-5) was employed to directly compare the carbamidocyclophane gene cluster with its homologues. Sequence data and the BLAST comparison files were drawn with the R package genoPlotR version 0.8.4 [77] and edited in Inkscape version 0.91.

Multiple sequence alignments of the PP-binding domains of acyl carrier proteins (ACPs) and of CabC and homologous sequences were calculated with MAFFT version 7.017 [78] using the L-INS-i algorithm and otherwise default parameters.

### 3.4. Bioassays

#### 3.4.1. Cytotoxicity Assay

The cytotoxicity was determined using the CellTiter-Blue assay and human adult low calcium high temperature keratinocytes (HaCaT), which were purchased from the German Cancer Research Center (DKFZ), Heidelberg, Germany. Testing was performed twice in triplicates with compound concentrations between 0.002 and 100 µg/mL as described previously [7]. Values are reported as average of calculated half maximal inhibitory concentration (IC_50_) values.

#### 3.4.2. Antimicrobial Assays

Antimicrobial evaluation on methicillin-resistant *S. aureus* 1 (MRSA) (B690208), *S. pneumoniae* 7 (B635308; also known as ATCC 49619), *E. coli* 13 (V676803), kanamycin-resistant *K. pneumoniae* 18 (KRKP) (B224106), and multidrug-resistant (MDR) *P. aeruginosa* 22 (V143708) was carried out determining the minimum inhibitory concentration (MIC) in accordance with the EUCAST criteria as previously described for these strains [7]. The bacterial strains have been isolated from infected patients and belong to the Sealife Pharma MDR pathogen collection. In-house strain designations are given in brackets. MIC assays were performed at least twice in triplicates using compound concentrations in the range 0.01‒50 µg/mL, and data shown are average values.

All other bacterial cultures were handled according to standard procedures and were obtained from the German Collection of Microorganisms and Cell Cultures (DSMZ), Braunschweig, Germany and the American Type Culture Collection (ATCC), Manassas, VA, United States, or were part of the internal strain collection of the HZI-HIPS (for respective strain designations, see Table 2). For microdilution assays, bacteria in mid-log phase were diluted to achieve a final inoculum of ca. 5 × 10^5^‒5 × 10^6^ cfu/mL in Tryptic Soy broth (1.7% peptone casein, 0.3% peptone soymeal, 0.25% glucose, 0.5% NaCl, 0.25% K_2_HPO_4_; pH 7.3; *E. faecium*, *S. pneumoniae*), M7H9 medium (Difco™ Middlebrook 7H9 broth supplemented with BBL^TM^ Middlebrook ADC enrichment and 2 mL/L glycerol; *M. bovis* DSM-43990 and *M. smegmatis* mc^2^ 155), Gym medium (0.4% glucose, 0.4% yeast extract, 1% malt extract; pH 7.2; *N. asteroides*) or Mueller-Hinton broth (1.75% casein hydrolysate, 0.2% beef infusion, 0.15% starch; pH 7.4; used for all other listed bacteria). *C. albicans* was grown in Myc medium (1% phytone peptone, 1% glucose, 50 mM HEPES, pH 7.0), *E. faecalis* and *S. pneumoniae* cultures were grown under microaerophilic conditions without shaking at 37 °C. All other listed microorganisms were grown on a shaker (200 rpm) at their optimal growth temperature. The vancomycin-intermediate *S. aureus* (VISA) strain Mu50 was cultured in the presence of 4 µg/mL vancomycin and *E. coli* was grown with or without PMBN (polymyxin B nonapeptide) at sublethal concentration (3 µg/mL) for permeabilization. Serial dilutions of crude extracts (0.5–1000 µg/mL) and purified compounds (0.03‒64 µg/mL) were prepared from MeOH stocks in sterile 96-well plates. The cell suspension was added and microorganisms were grown for 16‒20 h (2‒4 day for *M. bovis*, *M. smegmatis*, and *N. asteroides*). Shown MIC values are average data from two independent experiments.

Susceptibility testing on virulent *M. tuberculosis* strain H37Rv (ATCC 25618) was carried out using the MGIT 960 system as recommended by the manufacturer (Becton Dickinson, New Jersey, United States). We reduced the preset volumes of the MGIT vials of 7 mL by 2.782 mL. Tests with rifampicin were done in reduced volumes of the MGIT vials compared to those with 7 mL. The reduced volume had no impact on the efficiency of the test system. The MGIT tubes were supplemented with 0.482 mL OADC (BBL™ MGI™ OADC Enrichment; Becton Dickinson) and inoculated with 0.3 mL of *M. tuberculosis* suspension as recommended by the manufacturer. A 1:100 dilution of the bacterium was included as a compound-free growth control. Different compound concentrations ranging from 0.032 µg/mL to 128 µg/mL were used to obtain the MIC. The MGIT tubes were continuously monitored by the MGIT 960 system using the EpiCenter (version V5.80A) TB eXiST software (Becton Dickinson). The results were interpreted as previously described by Springer *et al.* [79]. Briefly, once the growth index of the growth control was >400, a growth index in the compound-containing vial of ≥100 was interpreted as resistant, whereas a compound-containing vial of <100 was interpreted as intermediate. Remained the growth index in the compound-containing vial <100 for another seven days after the growth control had reached a growth index of >400, the result was interpreted as sensitive. If the growth index was determined at least as intermediate, the MIC was obtained. Means were calculated from three independent experiments.

## 4. Conclusions

In this study, we showed that particularly the presence of bromide or chloride in the culture medium of *Nostoc* sp. CAVN2 resulted in a positive influence on both the growth and the biosynthesis of halogenated as well as non-halogenated carbamidocyclophanes. Supplementation of the culture medium with equimolar concentrations of bromide and chloride revealed that chloride appears to be the preferred halide substrate for the halogenase involved in [7.7]paracyclophane biosynthesis of strain CAVN2, but chloro-carbamidocyclophane generation is negatively affected in the presence of bromide.

Nevertheless, employing a combination of some key cultivation steps, such as the upstream-directed halide withholding and subsequent fermentation in individually halide-enriched culture medium, led to the isolation and structure elucidation of nine new brominated carbamidocyclophanes. First results of ongoing investigations to unravel the biosynthetic machinery required for carbamidocyclophane assembly in *Nostoc* sp. CAVN2 and the bioinformatic analysis of available [7.7]paracyclophane gene clusters indicate that the putative halogenase is highly conserved and consistently present. Nevertheless, as halogenated [7.7]paracyclophanes are not detected in all producing strains, they may not only be processed due to the occurrence and activity of this single enzyme, but arise most likely from an interaction with other proteins that are also involved in precursor elongation.

The remarkable antimicrobial activity of [7.7]paracyclophanes against Gram-positive bacteria, especially against MRSA, virulent *M. tuberculosis*, and PRSP, is mostly attributed to the unique framework. Additional functional groups attached to that macrocycle, such as hydroxy, acetoxy and carbamate moieties, or the presence as well as a varying degree of halogen atoms in the molecule, seem to have a rather modifying character for both the antimicrobial activity of [7.7]paracyclophanes and the cytotoxicity associated with these natural products. In some cases, however, as seen for the anti-*Mycobacterium* activity of carbamidocyclophane Q (**5**) or for the intra- and inter-species MRSA susceptibility testing of cylindrocyclophanes A_1_ (**21**) as well as D (**27**) and the cylindrofridins (**28**‒**30**), the replacement of specific substituents becomes of significant impact on the resulting bioactivity range. Whether chloro-bromo-hybrids of carbamido-/[7.7]paracyclophanes may mean an additional activity benefit, as reported in case of napyradiomycins [34,35], remains unanswered. Efforts regarding the directed biosynthesis of these derivatives and their isolation for bioactivity profiling are underway.

## Supplementary Files

Supplementary File 1
