# Supplementary material for: Effects of Halide Ions on the Carbamidocyclophane Biosynthesis in Nostoc sp. CAVN2
Source: Mar Drugs. 2016 Jan 20;14(1):21. doi: 10.3390/md14010021 (PMC4728517; doi:10.3390/md14010021)
Supplement: Supplementary File 1 [file marinedrugs-14-00021-s001.pdf]

# Supplementary Materials: Effects of Halide Ions on the Carbamidocyclophane Biosynthesis in *Nostoc* sp. CAVN2

**Michael Preisitsch, Stefan E. Heiden, Monika Beerbaum, Timo H. J. Niedermeyer, Marie Schneefeld, Jennifer Herrmann, Jana Kumpfmüller, Andrea Thürmer, Inga Neidhardt, Christoph Wiesner, Rolf Daniel, Rolf Müller, Franz-Christoph Bange, Peter Schmieder, Thomas Schweder and Sabine Mundt**

**Table S1.** Composition of Z½ medium used in this study.

| Ingredient | mg/L $H_2O$ |
|---|---|
| *Macronutrients* [a] | |
| $NaNO_3$ | 233.5 |
| $Ca(NO_3)_2{\cdot}4H_2O$ | 29.5 |
| $K_2HPO_4$ | 15.5 |
| $MgSO4{\cdot}7H_2O$ | 12.5 |
| $Na_2CO_3$ | 10.6 |
| *Micronutrients* [b] | |
| $H_3BO_3$ | 0.24800 |
| $MnSO_4{\cdot}4H_2O$ | 0.17840 |
| KBr | 0.00952 |
| KI | 0.00664 |
| $ZnSO_4{\cdot}7H_2O$ | 0.02296 |
| $(NH_4)_6Mo_7O_{24}{\cdot}4H_2O$ | 0.00704 |
| $Na_2WO_4{\cdot}2H_2O$ | 0.00264 |
| $KCr(SO_4)_2{\cdot}12H_2O$ | 0.00400 |
| $AlK(SO_4)_2{\cdot}12H_2O$ | 0.07584 |
| $Cd(NO_3)_2{\cdot}4H_2O$ | 0.01232 |
| $Co(NO_3)_2{\cdot}6H_2O$ | 0.01168 |
| $CuSO_4$ | 0.00640 |
| $NiSO_4{\cdot}7H_2O$ | 0.01128 |
| $NH_4VO_3$ | 0.00184 |
| *Other Components* [c] | |
| $FeSO_4{\cdot}7H_2O$ | 5.0 |
| $Na_2EDTA$ | 20.0 |

[a] Half quantity of macronutrients found in Zehnder-Medium according to Falch *et al.* [1]; [b] Micronutrient according to Gaffron's solution reported by Hughes *et al.* [2] in modified form; [c] Fe-EDTA-supplement according to Meffert *et al.* [3] in modified form.

**Table S2.** Biomass dry weights of *Nostoc* sp. CAVN2 cultures grown in the presence of different halogen salts at varying concentrations for 20–30 days. Values are presented as mean ± standard error of the mean (SEM), $n = 2$.

| **CAVN2 Culture** | **Biomass Dry Weight (g/L)** | | |
|---|---|---|---|
| | **20 Days** | **25 Days** | **30 Days** |
| Control | 0.39 ± 0.03 | 0.40 ± 0.05 | 0.47 ± 0.00 |
| +0.001% KF | 0.40 ± 0.02 | 0.46 ± 0.02 | 0.50 ± 0.02 |
| +0.01% KF | 0.42 ± 0.01 | 0.42 ± 0.04 | 0.50 ± 0.04 |
| +0.1% KF | 0.28 ± 0.03 | 0.33 ± 0.03 | 0.39 ± 0.03 |
| +1.0% KF | 0.17 ± 0.01 | 0.20 ± 0.02 | 0.23 ± 0.02 |
| +0.001% KI | 0.36 ± 0.00 | 0.39 ± 0.01 | 0.44 ± 0.05 |
| +0.01% KI | 0.36 ± 0.03 | 0.43 ± 0.04 | 0.43 ± 0.03 |
| +0.1% KI | 0.20 ± 0.00 | 0.25 ± 0.02 | 0.38 ± 0.11 |
| +1.0% KI | 0.17 ± 0.03 | 0.15 ± 0.05 | 0.19 ± 0.05 |
| +0.001% KBr | 0.38 ± 0.04 | 0.51 ± 0.04 | 0.45 ± 0.05 |
| +0.01% KBr | 0.36 ± 0.02 | 0.46 ± 0.05 | 0.51 ± 0.01 |
| +0.1% KBr | 0.32 ± 0.04 | 0.43 ± 0.00 | 0.40 ± 0.02 |
| +1.0% KBr | 0.44 ± 0.03 | 0.47 ± 0.02 | 0.48 ± 0.07 |
| +0.001% KCl | 0.45 ± 0.08 | 0.65 ± 0.06 | 0.52 ± 0.00 |
| +0.01% KCl | 0.41 ± 0.04 | 0.49 ± 0.11 | 0.54 ± 0.06 |
| +0.1% KCl | 0.38 ± 0.05 | 0.42 ± 0.04 | 0.52 ± 0.05 |
| +1.0% KCl | 0.50 ± 0.05 | 0.54 ± 0.01 | 0.63 ± 0.00 |

**Table S3.** $^{1}$H (600 MHz,) and $^{13}$C NMR (150 MHz) spectroscopic data for compounds **1–5** in MeOH-$d_4$ [a].

| No. [b] | 1 | | 2 | | 3 | | 4 | | 5 | |
|---|---|---|---|---|---|---|---|---|---|---|
| | $\delta_C$, Type | $\delta_H$ (*J* in Hz) | $\delta_C$, Type | $\delta_H$ (*J* in Hz) | $\delta_C$, Type | $\delta_H$ (*J* in Hz) | $\delta_C$, Type | $\delta_H$ (*J* in Hz) | $\delta_C$, Type | $\delta_H$ (*J* in Hz) |
| 1 | 84.9, CH | 4.93, *d* (10.1) | 84.9, CH | 4.90, *d* (10.4) | 84.9, CH | 4.93, *d* (10.1) | 84.6, CH | 4.90, *d* (10.1) | 84.8, CH | 4.90, *d* (10.2) |
| 2 | 41.7, CH | 1.81, *m* | 41.7, CH | 1.81, *m* | 41.8, CH | 1.84, *m* | 41.8, CH | 1.81, *m* | 41.7, CH | 1.81, *m* |
| 3 | 35.9, $CH_2$ | 0.87, 0.79, *m* | 36.0, $CH_2$ | 0.87, 0.79, *m* | 35.9, $CH_2$ | 0.91, 0.82, *m* | 35.8, $CH_2$ | 0.87, 0.80, *m* | 35.8, $CH_2$ | 0.87, 0.80, *m* |
| 4 | 31.0, $CH_2$ | 1.52, n.o., *m* | 31.0, $CH_2$ | 1.52, n.o., *m* | 31.0, $CH_2$ | 1.56, 0.92, *m* | 30.9, $CH_2$ | 1.53, 0.92, *m* | 30.9, $CH_2$ | 1.53, 0.92, *m* |
| 5 | 31.7, $CH_2$ | 1.03, 0.81, *m* | 31.8, $CH_2$ | 1.04, 0.81, *m* | 31.8, $CH_2$ | 1.07, 0.84, *m* | 31.8, $CH_2$ | 1.05, 0.81, *m* | 31.8, $CH_2$ | 1.05, 0.81, *m* |
| 6 | 36.6, $CH_2$ | 2.12, 1.41, *m* | 36.6, $CH_2$ | 2.13, 1.41, *m* | 36.6, $CH_2$ | 2.15, 1.44, *m* | 36.6, $CH_2$ | 2.13, 1.42, *m* | 36.6, $CH_2$ | 2.13, 1.42, *m* |
| 7 | 37.9, CH | 3.27, *m* | 37.9 | 3.27, *m* | 37.7, CH | 3.31, *m* | 37.7, CH | 3.28, *m* | 37.7, CH | 3.28, *m* |
| 8 | 119.1, C | | 119.1, C | | 119.7, C | | 119.9, C | | 118.7, C | |
| 9 | 158.4, C | | 160.2, C | | 160.2, C | | 161.2, C | | 160.2, C | |
| 10 | 106.6, CH | 6.28, *s* | 106.6, CH | 6.28, *s* | 106.6, CH | 6.24, *s* | 106.8, CH | 6.22, *s* | 106.6, CH | 6.22, *s* |
| 11 | 141.2, C | | 141.3, C | | 141.4, C | | 141.8, C | | 141.5, C | |
| 12 | 110.7, CH | 6.21, *s* | 110.7, CH | 6.21, *s* | 110.8, CH | 6.31, *s* | 110.8, CH | 6.28, *s* | 110.8, CH | 6.28, *s* |
| 13 | 158.4, C | | 158.5, C | | 158.4, C | | 158.3, C | | 158.4, C | |
| 14 | 84.9, CH | 4.93, *d* (10.1) | 84.9, CH | 4.90, *d* (10.4) | 84.9, CH | 4.93, *d* (10.1) | 84.6, CH | 4.90, *d* (10.1) | 84.8, CH | 4.90, *d* (10.2) |
| 15 | 41.7, CH | 1.81, *m* | 41.7, CH | 1.81, *m* | 41.8, CH | 1.84, *m* | 41.8, CH | 1.81, *m* | 41.7, CH | 1.81, *m* |
| 16 | 35.9, $CH_2$ | 0.87, 0.79, *m* | 36.0, $CH_2$ | 0.87, 0.79, *m* | 35.9, $CH_2$ | 0.91, 0.82, *m* | 35.8, $CH_2$ | 0.87, 0.80, *m* | 35.8, $CH_2$ | 0.87, 0.80, *m* |
| 17 | 31.0, $CH_2$ | 1.52, n.o., *m* | 31.0, $CH_2$ | 1.52, n.o., *m* | 31.0, $CH_2$ | 1.56, 0.92, *m* | 30.9, $CH_2$ | 1.53, 0.92, *m* | 30.9, $CH_2$ | 1.53, 0.92, *m* |
| 18 | 31.7, $CH_2$ | 1.03, 0.81, *m* | 31.8, $CH_2$ | 1.04, 0.81, *m* | 31.8, $CH_2$ | 1.07, 0.84, *m* | 31.8, $CH_2$ | 1.05, 0.81, *m* | 31.8, $CH_2$ | 1.05, 0.81, *m* |
| 19 | 36.6, $CH_2$ | 2.12, 1.41, *m* | 36.6, $CH_2$ | 2.13, 1.41, *m* | 36.6, $CH_2$ | 2.15, 1.44, *m* | 36.6, $CH_2$ | 2.13, 1.42, *m* | 36.6, $CH_2$ | 2.13, 1.42, *m* |
| 20 | 38.1, CH | 3.24, *m* | 37.9 | 3.27, *m* | 38.2, CH | 3.27, *m* | 37.7, CH | 3.28, *m* | 37.7, CH | 3.28, *m* |
| 21 | 119.7, C | | 119.1, C | | 118.7, C | | 119.9, C | | 118.7, C | |
| 22 | 158.4, C | | 160.2, C | | 160.2, C | | 161.2, C | | 160.2, C | |
| 23 | 106.6, CH | 6.28, *s* | 106.6, CH | 6.28, *s* | 106.6, CH | 6.24, *s* | 106.8, CH | 6.22, *s* | 106.6, CH | 6.22, *s* |
| 24 | 141.2, C | | 141.3, C | | 141.4, C | | 141.8, C | | 141.5, C | |
| 25 | 110.7, CH | 6.21, *s* | 110.7, CH | 6.21, *s* | 110.8, CH | 6.31, *s* | 110.8, CH | 6.28, *s* | 110.8, CH | 6.28, *s* |
| 26 | 158.4, C | | 158.5, C | | 158.4, C | | 158.3, C | | 158.4, C | |
| 27 | 35.4, $CH_2$ | 2.06, 1.60, *m* | | 2.06, 1.59, *m* | 34.7, $CH_2$ | 2.14, 1.64, *m* | 34.7, $CH_2$ | 2.11, 1.61, *m* | 34.7, $CH_2$ | 2.11, 1.61, *m* |
| 28 | 29.3, $CH_2$ | 1.39, 1.32, *m* | 29.3, $CH_2$ | 1.39, 1.32, *m* | 29.2, $CH_2$ | 1.64, 1.49, *m* | 29.2, $CH_2$ | 1.61, 1.46, *m* | 29.2, $CH_2$ | 1.61, 1.46, *m* |
| 29 | 35.8, $CH_2$ | 1.90, 1.84, *m* | 35.8, $CH_2$ | 1.91, 1.83, *m* | 48.3, $CH_2$ | 2.50, 2.38, *m* | 48.3, $CH_2$ | 2.47, 2.35, *m* | 48.3, $CH_2$ | 2.47, 2.35, *m* |
| 30 | 35.8, $CH_2$ | 3.42, *td* (7.0, 0.9) | 35.8, $CH_2$ | 3.42, *td* (7.1, 0.8) | 49.3, CH | 5.91, *t* (6.3) | 49.3, CH | 5.88, *t* (6.4) | 49.3, CH | 5.88, *t* (6.3) |

**Table S3.** *Cont.*

| | | | | | | | | | | |
|---|---|---|---|---|---|---|---|---|---|---|
| 31 | 36.2, $CH_2$ | 2.00, 1.58, *m* | 35.4, $CH_2$ | 2.06, 1.59, *m* | 36.2, $CH_2$ | 2.03, 1.61, *m* | 35.4, $CH_2$ | 2.06, 1.60, *m* | 34.7, $CH_2$ | 2.11, 1.61, *m* |
| 32 | 33.0, $CH_2$ | 1.29, 1.19, *m* | 29.3, $CH_2$ | 1.39, 1.32, *m* | 33.0, $CH_2$ | 1.29, 1.19, *m* | 29.1, $CH_2$ | 1.46, 1.33, *m* | 29.2, $CH_2$ | 1.61, 1.46, *m* |
| 33 | 25.3, $CH_2$ | 1.33, n.o., *m* | 35.8, $CH_2$ | 1.91, 1.83, *m* | 25.3, $CH_2$ | 1.40, 1.34, *m* | 35.7, $CH_2$ | 1.91, 1.84, *m* | 48.3, $CH_2$ | 2.47, 2.35, *m* |
| 34 | 15.8, $CH_3$ | 0.90, *t* (7.2) | 35.8, $CH_2$ | 3.42, *td* (7.1, 0.8) | 15.9, $CH_3$ | 0.93, *t* (7.2) | 35.8, $CH_2$ | 3.42, *td* (7.0, 1.2) | 49.3, CH | 5.88, *t* (6.3) |
| 35 | 17.9, $CH_3$ | 1.09, *d* (6.4) | 17.9, $CH_3$ | 1.09, *d* (6.4) | 17.9, $CH_3$ | 1.12, *d* (6.4) | 17.9, $CH_3$ | 1.09, *d* (6.4) | 17.9, $CH_3$ | 1.09, *d* (6.4) |
| 36 | 17.9, $CH_3$ | 1.09, *d* (6.4) | 17.9, $CH_3$ | 1.09, *d* (6.4) | 17.9, $CH_3$ | 1.12, *d* (6.4) | 17.9, $CH_3$ | 1.09, *d* (6.4) | 17.9, $CH_3$ | 1.09, *d* (6.4) |
| 37 | 161.3, C | | 161.2, C | | 161.3, C | | 162.3, C | | 161.2, C | |
| 38 | 161.3, C | | 161.2, C | | 161.3, C | | 162.3, C | | 161.2, C | |

[a] $^{13}C$ chemical shifts obtained from HMQC and HMBC spectra; Abbreviation: n.o. = not observed; [b] Numbering of the carbamidocyclophane framework, see Figure 4A.

**Table S4.** $^{1}H$ (600 MHz) and $^{13}C$ NMR (150 MHz) spectroscopic data for compounds **6–9** in MeOH-$d_4$ [a].

| No.[b] | 6 | | 7 | | 8 | | 9 | |
|---|---|---|---|---|---|---|---|---|
| | **δC, Type** | **δH (*J* in Hz)** | **δC, Type** | **δH (*J* in Hz)** | **δC, Type** | **δH (*J* in Hz)** | **δC, Type** | **δH (*J* in Hz)** |
| 1 | 84.9, CH | 4.93, *d* (10.4) | 84.9, CH | 4.90, *d* (10.3) | 84.9, CH | 4.90, *d* (10.6) | 85.0, CH | 4.89, *d* (10.4) |
| 2 | 41.8, CH | 1.85, *m* | 41.8, CH | 1.82, *m* | 41.8, CH | 1.82, *m* | 41.7, CH | 1.82, *m* |
| 3 | 35.9, $CH_2$ | 0.89, 0.79, *m* | 35.9, $CH_2$ | 0.88, 0.79, *m* | 35.9, $CH_2$ | 0.88, 0.80, *m* | 35.9, $CH_2$ | 0.88, 0.79, *m* |
| 4 | 31.2, $CH_2$ | 1.54, n.o., *m* | 31.2, $CH_2$ | 1.51, 0.91, *m* | 31.1, $CH_2$ | 1.51, 0.90, *m* | 31.0, $CH_2$ | 1.53, 0.90, *m* |
| 5 | 31.9, $CH_2$ | 1.03, 0.80, *m* | 31.9, $CH_2$ | 1.04, 0.80, *m* | 31.8, $CH_2$ | 1.03, 0.81, *m* | 31.8, $CH_2$ | 1.03, 0.81, *m* |
| 6 | 36.8, $CH_2$ | 2.15, 1.43, *m* | 36.7, $CH_2$ | 2.13, 1.41, *m* | 36.7, $CH_2$ | 2.13, 1.41, *m* | 36.7, $CH_2$ | 2.14, 1.41, *m* |
| 7 | 37.9, CH | 3.30, *m* | 37.7, CH | 3.28, *m* | 37.8, CH | 3.28, *m* | 37.7, CH | 3.28, *m* |
| 8 | 119.2, 119.7, C | | 119.2, 119.7, C | | 119.2, 119.7, C | | 119.9, C | |
| 9 | 160.3, C | | 160.3, C | | 160.2, C | | 160.3, C | |
| 10 | 106.6, CH | 6.32, *s* | 106.7, CH | 6.31, *s* | 106.6, CH | 6.30, *s* | 106.9, CH | 6.29, *s* |
| 11 | 141.2, C | | 141.3, C | | 141.4, C | | 141.8, C | |
| 12 | 110.8, CH | 6.20, *s* | 110.9, CH | 6.24, *s* | 110.8, CH | 6.22, *s* | 111.0, CH | 6.22, *s* |
| 13 | 158.4, C | | 158.4, C | | 158.4, C | | n.o. | |
| 14 | 83.1, CH | 3.86, *d* (9.8) | 83.1, CH | 3.84, *d* (9.6) | 83.1 CH | 3.84, *d* (9.5) | 83.2, CH | 3.83, *d* (9.5) |
| 15 | 43.4, CH | 1.66, *m* | 43.4, CH | 1.63, *m* | 43.4, CH | 1.63, *m* | 43.4, CH | 1.63, *m* |
| 16 | 36.6, $CH_2$ | 0.85, 0.74, *m* | 36.6, $CH_2$ | 0.83, 0.72, *m* | 36.5, $CH_2$ | 0.82, 0.71, *m* | 36.5, $CH_2$ | 0.83, 0.73, *m* |

**Table S4.** *Cont.*

| | | | | | | | | |
|---|---|---|---|---|---|---|---|---|
| 17 | 31.2, $CH_2$ | 1.54, n.o., *m* | 31.2, $CH_2$ | 1.51, 0.91, *m* | 31.1, $CH_2$ | 1.51, 0.91, *m* | 31.1, $CH_2$ | 1.51, n.o., *m* |
| 18 | 31.9, $CH_2$ | 1.03, 0.80, *m* | 31.9, $CH_2$ | 1.04, 0.80, *m* | 31.8, $CH_2$ | 1.03, 0.81, *m* | 31.8, $CH_2$ | 1.03, 0.81, *m* |
| 19 | 36.8, $CH_2$ | 2.15, 1.43, *m* | 36.7, $CH_2$ | 2.13, 1.41, *m* | 36.7, $CH_2$ | 2.13, 1.41, *m* | 36.7, $CH_2$ | 2.14, 1.41, *m* |
| 20 | 38.2, CH | 3.27, *m* | 38.2, CH | 3.24, *m* | 37.8, CH | 3.28, *m* | 37.7, CH | 3.28, *m* |
| 21 | 118.5, 119.0, C | | 118.5, 119.0, C | | 118.5, 119.0, C | | 118.3, C | |
| 22 | 160.2, C | | 160.4, C | | 160.3, C | | 160.3, C | |
| 23 | 106.4, CH | 6.18, *s* | 106.5, CH | 6.36, *s* | 106.4, CH | 6.34, *s* | 106.6, CH | 6.33, *s* |
| 24 | 145.4, C | | 145.5, C | | 145.6, C | | 146.0, C | |
| 25 | 110.2, CH | 6.35, *s* | 110.3, CH | 6.19, *s* | 110.2, CH | 6.16, *s* | 110.4, CH | 6.16, *s* |
| 26 | 158.4, C | | 158.5, C | | 158.4, C | | n.o. | |
| 27 | 35.4, $CH_2$ | 2.11, 1.61, *m* | 35.4, $CH_2$ | 2.06, 1.59, *m* | 34.8, $CH_2$ | 2.13, 1.60, *m* | 34.7, $CH_2$ | 2.12, 1.60, *m* |
| 28 | 29.2, $CH_2$ | 1.38, 1.33, *m* | 29.2, $CH_2$ | 1.60, 1.45, *m* | 29.2, $CH_2$ | 1.60, 1.45, *m* | 29.1, $CH_2$ | 1.60, 1.45, *m* |
| 29 | 35.8, $CH_2$ | 1.93, n.o., *m* | 48.3, $CH_2$ | 2.49, 2.35, *m* | 48.2, $CH_2$ | 2.48, 2.34, *m* | 48.3, $CH_2$ | 2.48, 2.34, *m* |
| 30 | 35.9, $CH_2$ | 3.43, *m* | 49.4, CH | 5.89, *t* (6.3) | 49.4, CH | 5.89, *td* (6.3, 1.4) | 49.3, CH | 5.88, *td* (6.3, 1.4) |
| 31 | 36.2, $CH_2$ | 2.03, 1.59, *m* | 36.2, $CH_2$ | 2.01, 1.57, *m* | 35.3, $CH_2$ | 2.08, 1.59, *m* | 34.7, $CH_2$ | 2.12, 1.60, *m* |
| 32 | 33.1, $CH_2$ | 1.28,1.17, *m* | 33.2, $CH_2$ | 1.26, 1.19, *m* | 29.2, $CH_2$ | 1.42, 1.35, *m* | 29.1, $CH_2$ | 1.60, 1.45, *m* |
| 33 | 25.3, $CH_2$ | 1.40, 1.32, *m* | 25.3, $CH_2$ | 1.37, 1.28, *m* | 35.5, $CH_2$ | 1.91, 1.84, *m* | 48.3, $CH_2$ | 2.48, 2.34, *m* |
| 34 | 15.9, $CH_3$ | 0.92, *m* | 15.9, $CH_3$ | 0.90, *m* | 35.9, $CH_2$ | 3.41, *m* | 49.3, CH | 5.88, *td* (6.3, 1.4) |
| 35 | 18.0, $CH_3$ | 1.12, *d* (6.1) | 18.0, $CH_3$ | 1.09, *d* (6.3) | 17.9, $CH_3$ | 1.09, *d* (6.4) | 17.9, $CH_3$ | 1.09, *d* (6.4) |
| 36 | 18.3, $CH_3$ | 1.17, *d* (6.1) | 18.3, $CH_3$ | 1.15, *d* (6.3) | 18.3, $CH_3$ | 1.14, *d* (6.4) | 18.3, $CH_3$ | 1.15, *d* (6.4) |
| 37 | 161.3, C | | 161.3, C | | 161.2, C | | 161.5, C | |

[a] $^{13}C$ chemical shifts obtained from HMQC and HMBC spectra; Abbreviation: n.o. = not observed; [b] Numbering of the carbamidocyclophane framework, see Figure 4A.

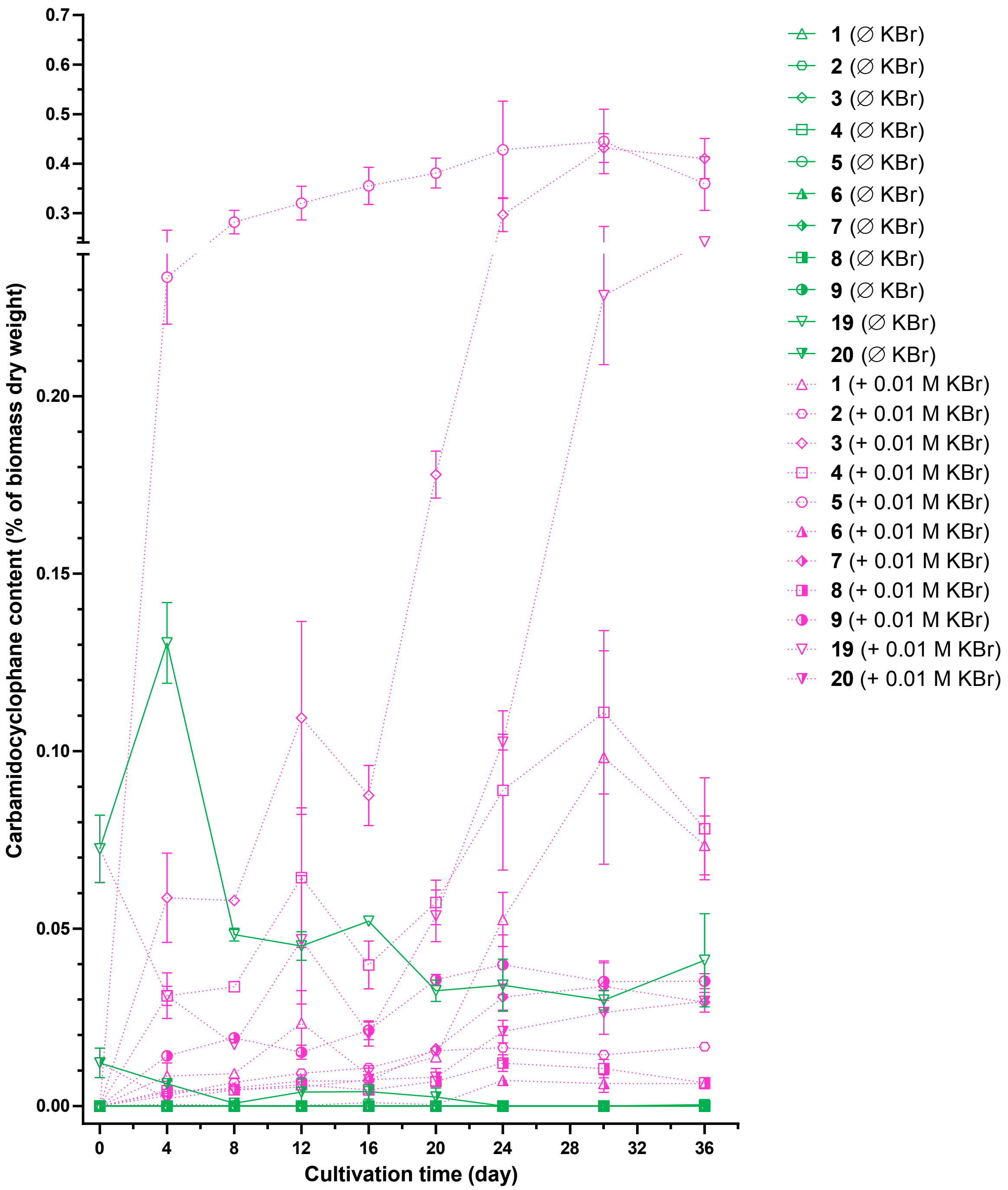


**Figure S1.** Intracellular contents of brominated (**1**–**9**) and non-halogenated (**19** and **20**) carbamidocyclophanes of *Nostoc* sp. CAVN2 cultivated in Z½ medium as control containing <0.1 μM halide ions (Ø KBr) or in bromide-enriched (+0.01 M KBr) Z½ medium. Values shown are expressed as the mean ± SEM, *n* = 3.

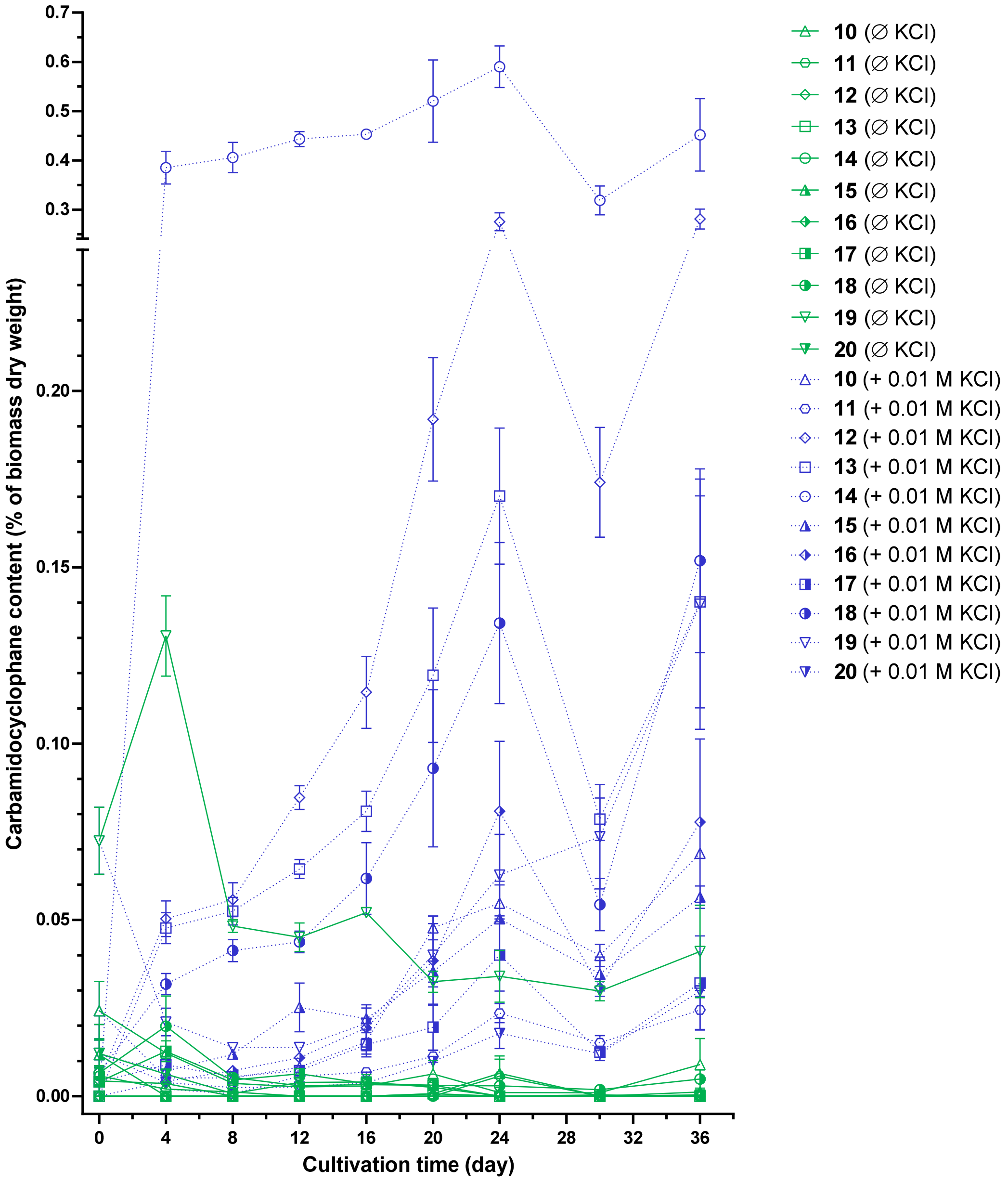


**Figure S2.** Intracellular contents of chlorinated (**10**–**18**) and non-halogenated (**19** and **20**) carbamidocyclophanes of *Nostoc* sp. CAVN2 cultivated in Z½ medium as control containing <0.1 µM halide ions (Ø KCl) or in chloride-enriched (+ 0.01 M KCl) Z½ medium. Values shown are expressed as the mean ± SEM, $n = 3$.

**Table S5.** Biomass dry weights of *Nostoc* sp. CAVN2 cultures grown in the presence of KCl and KBr at different mixing ratios for 20 days. Values are presented as mean ± SEM, $n = 3$.

| CAVN2 Culture | Biomass Dry Weight (g/L) | Statistical Significance *versus* the Control [a] |
|---|---|---|
| Control | 0.35 ± 0.01 | |
| +0.01 M KCl and 0.01 M KBr | 0.77 ± 0.03 | ** |
| +0.01 M KCl and 0.001 M KBr | 0.68 ± 0.02 | No |
| +0.001 M KCl and 0.01 M KBr | 0.64 ± 0.03 | No |

[a] Two asterisks (**) indicate a significant difference of $p < 0.01$ versus the control; Data were tested for Gaussian distribution using the Kolmogorov-Smirnov test; Statistical analysis was performed using the Kruskal-Wallis test followed by Dunn's multiple comparisons test.

**Table S6.** List of predicted open reading frames (ORF) of the *cab* gene cluster [a].

| ORF | Annotation | Protein | Amino Acids | Top BlastP Result–Protein [Organism] (Acc. No.) | Ident/Sim/Qcov (%), E-Value | InterPro Results |
|---|---|---|---|---|---|---|
| 1 | Caspase-like domain-containing protein | | 220 | Hypothetical protein [*Scytonema hofmanni* UTEX B 1581] (WP_051502726) | 56/68/87.27, 8.16e-57 | Caspase-like domain(IPR029030) |
| 2 | Long-chain-fatty-acid—CoA ligase | CabA | 604 | AMP-dependent synthetase/ligase [*Cylindrospermum licheniforme* UTEX B 2014] (AFV96135) | 88/94/99.83, 0 | AMP-dependent synthetase/ligase (IPR000873) |
| 3 | Acyl carrier protein | CabB | 103 | Acyl carrier protein [*C. licheniforme* UTEX B 2014] (AFV96136) | 87/90/100, 1.61e-57 | Acyl carrier protein-like (IPR009081) |
| 4 | Hypothetical protein | CabC | 471 | Hypothetical protein [*C. licheniforme* UTEX B 2014] (AFV96137) | 96/97/100, 0 | None |
| 5 | Polyketide synthase | CabD | 1,385 | Polyketide synthase [*C. licheniforme* UTEX B 2014] (AFV96138) | 88/94/77.11, 0 | Polyketide synthase, beta-ketoacyl synthase domain (IPR020841), acyl transferase domain (IPR020801), 3× phosphopantetheine-binding domain (IPR020806) |
| 6 | Beta-ketoacyl synthase | CabE | 413 | Beta-ketoacyl synthase [*C. licheniforme* UTEX B 2014] (AFV96139) | 92/96/100, 0 | Polyketide synthase, beta-ketoacyl synthase domain (IPR020841) |
| 7 | Hydroxymethylglutaryl-CoA synthase | CabF | 419 | 3-hydroxy-3-methylglutaryl CoA synthase [*Cylindrospermum stagnale*] (WP_015207399) | 94/98/100, 0 | Hydroxymethylglutaryl-coenzyme A synthase, *N*-terminal (IPR013528), *C*-terminal domain (IPR013746) |
| 8 | Enoyl-CoA hydratase | CabG | 258 | Enoyl-CoA hydratase/isomerase [*C. licheniforme* UTEX B 2014] (AFV96141) | 90/95/98.45, 1.41e-162 | Crotonase superfamily (IPR001753) |

**Table S6.** *Cont.*

| | | | | | | |
|---|---|---|---|---|---|---|
| 9 | Polyketide synthase | CabH | 2077 | Polyketide synthase family protein [*C. stagnale*] (WP_015207397) | 84/91/68.85, 0 | Crotonase superfamily (IPR001753); Polyketide synthase, enoylreductase domain (IPR020843), beta-ketoacyl Synthase domain (IPR020841), acyl transferase domain (IPR020801), phosphopantetheine-binding domain (IPR020806) thioesterase (IPR001031) |
| 10 | Type III polyketide synthase | CabI | 373 | Naringenin-chalcone synthase [*C. stagnale*] (WP_015207396) | 92/97/100, 0 | FAE1/Type III polyketide synthase-like protein (IPR013601); chalcone/stilbene synthase, C-terminal (IPR012328) |
| 11 | Isoprenylcysteine carboxyl methyltransferase | CabJ | 199 | Phospholipid methyltransferase [*C. licheniforme* UTEX B 2014] (AFV96144) | 84/94/100, 3.66e-122 | Isoprenylcysteine carboxyl methyltransferase (IPR007269) |
| 12 | Beta-propeller repeat-containing protein | CabK | 687 | Hemolysin-type calcium-binding region [*C. licheniforme* UTEX B 2014] (AFV96145) | 79/90/92.87, 0 | Quinonprotein alcohol dehydrogenase-like superfamily (IPR011047) Beta-propeller repeat (IPR010620) |
| 13 | Carbamoyltransferase | CabL | 581 | Hypothetical protein [Methylococcaceae bacterium Sn10-6] (WP_052700247) | 52/68/71.08, 6.74e-150 | Carbamoyltransferase (IPR003696) |
| 14 | Rieske [2Fe-2S] iron-sulphur domain-containing protein | CabM | 502 | Ring-hydroxylating dioxygenase [*C. stagnale*] (WP_015207391) | 89/95/100, 0 | Rieske [2Fe-2S] iron-sulphur domain (IPR017941); pheophorbide a oxygenase (IPR013626) |

[a] Abbreviations: Acc. No. = accession number; Ident = identity, Sim = similarity, Qcov = query coverage, E-value = expectation value.

**Table S7.** Results of the MegaBLAST search of the *C. licheniforme* UTEX "B 2014" *cyl* gene cluster (query) against the *Nostoc* sp. CAVN2 *cab* gene cluster (subject).

| Identity (%) | Alignment Length | Mismatches | Gap Opens | Query | | Subject | | E-Value | Bit Score |
|---|---|---|---|---|---|---|---|---|---|
| | | | | Start | End | Start | End | | |
| 88.78 | 1880 | 206 | 4 | 1005 | 2879 | 1337 | 3216 | 0 | 2298.0 |
| 84.60 | 448 | 51 | 10 | 3345 | 3788 | 3275 | 3708 | 4e-121 | 429.0 |
| 91.08 | 4954 | 423 | 9 | 3924 | 8865 | 3833 | 8779 | 0 | 6682.0 |
| 85.01 | 467 | 68 | 2 | 8478 | 8943 | 8791 | 9256 | 2e-134 | 473.0 |
| 89.56 | 5767 | 573 | 19 | 8886 | 14646 | 9276 | 15019 | 0 | 7287.0 |
| 83.51 | 97 | 16 | 0 | 14691 | 14787 | 14923 | 15019 | 2e-19 | 91.6 |
| 76.68 | 223 | 36 | 7 | 14832 | 15054 | 14923 | 15129 | 5e-25 | 110.0 |
| 87.72 | 6148 | 687 | 40 | 15058 | 21189 | 15044 | 21139 | 0 | 7108.0 |
| 85.66 | 1980 | 273 | 10 | 21414 | 23388 | 21468 | 23441 | 0 | 2073.0 |

**Table S8.** Results of the MegaBLAST search of the *Nostoc* sp. CAVN2 *cab* gene cluster (query) against the *Cylindrospermum stagnale* PCC 7417 gene cluster (subject).

| Identity (%) | Alignment Length | Mismatches | Gap Opens | Query | | Subject | | E-Value | Bit Score |
|---|---|---|---|---|---|---|---|---|---|
| | | | | Start | End | Start | End | | |
| 88.46 | 1881 | 207 | 6 | 1337 | 3216 | 797 | 2668 | 0 | 2263.0 |
| 89.75 | 5979 | 565 | 25 | 3275 | 9223 | 2853 | 8813 | 0 | 7601.0 |
| 86.36 | 396 | 54 | 0 | 8384 | 8779 | 8372 | 8767 | 3e-122 | 433.0 |
| 85.27 | 387 | 57 | 0 | 8791 | 9177 | 7981 | 8367 | 3e-122 | 399.0 |
| 89.64 | 5744 | 565 | 20 | 9300 | 15019 | 8799 | 14536 | 0 | 7284.0 |
| 82.47 | 97 | 17 | 0 | 14923 | 15019 | 14581 | 14677 | 1e-17 | 86.1 |
| 83.51 | 97 | 16 | 0 | 14923 | 15019 | 14722 | 14818 | 2e-19 | 91.6 |
| 87.07 | 6282 | 726 | 43 | 14923 | 21139 | 14863 | 21123 | 0 | 7023.0 |
| 86.34 | 1999 | 264 | 8 | 21468 | 23461 | 21349 | 23343 | 0 | 2170.0 |
| 91.51 | 106 | 8 | 1 | 23775 | 23880 | 23650 | 23754 | 2e-35 | 145.0 |
| 95.31 | 128 | 6 | 0 | 23871 | 23998 | 24026 | 24153 | 3e-53 | 204.0 |
| 87.25 | 659 | 55 | 13 | 25853 | 26482 | 24185 | 24843 | 0 | 725.0 |
| 90.80 | 1630 | 135 | 4 | 26727 | 28341 | 25970 | 27599 | 0 | 2165.0 |

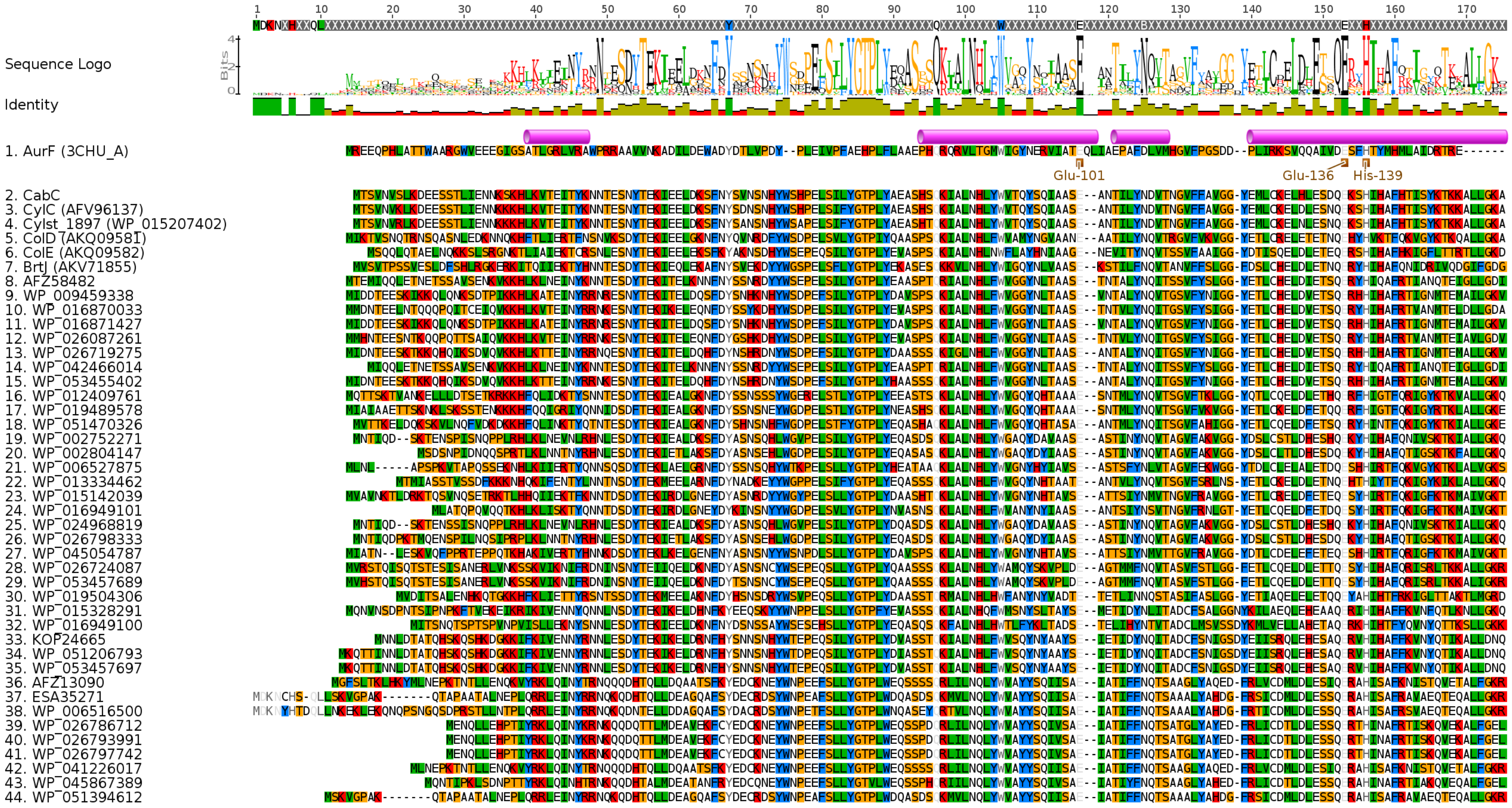


**Figure S3.** *Cont.*

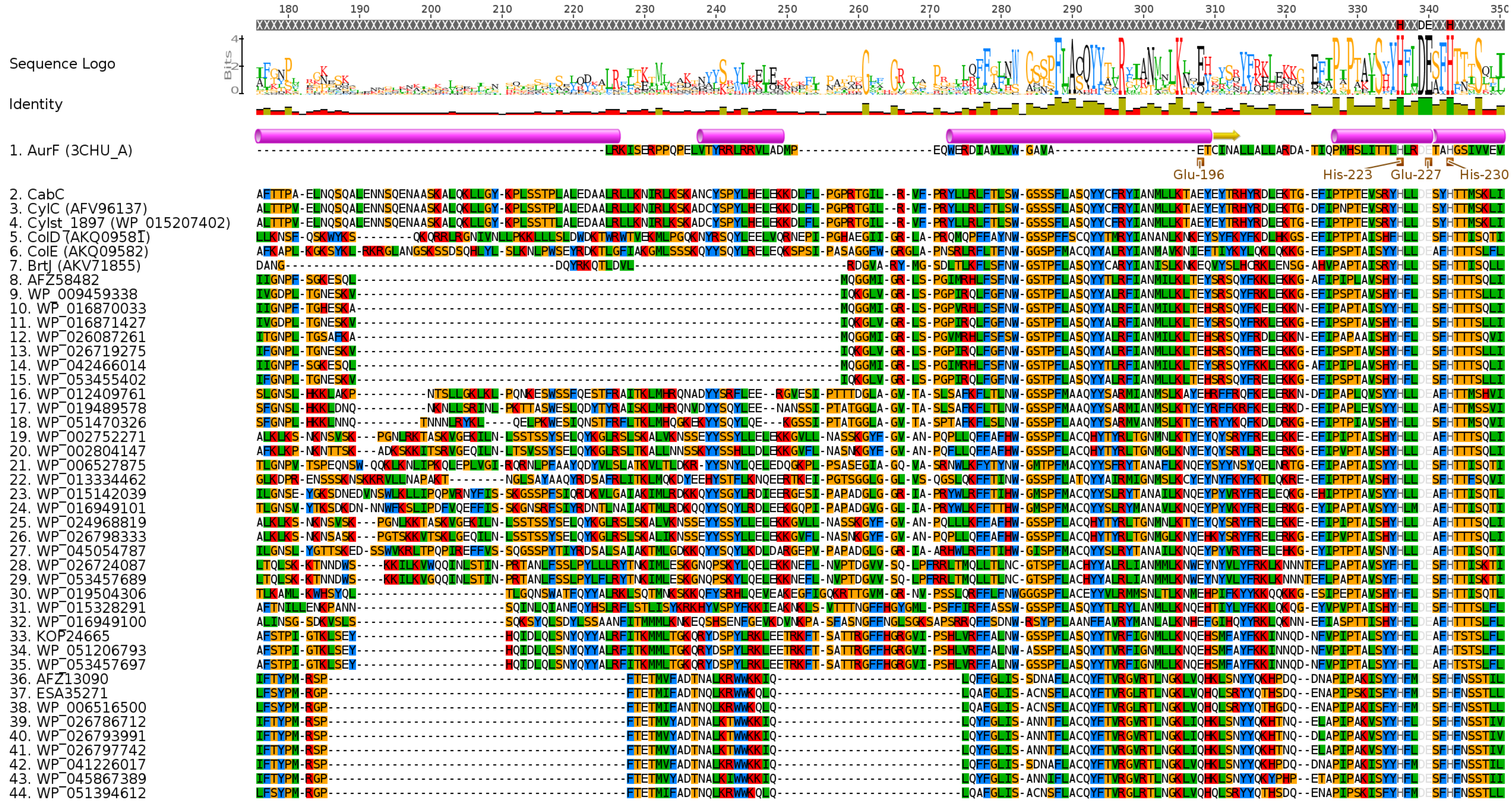


**Figure S3.** *Cont.*

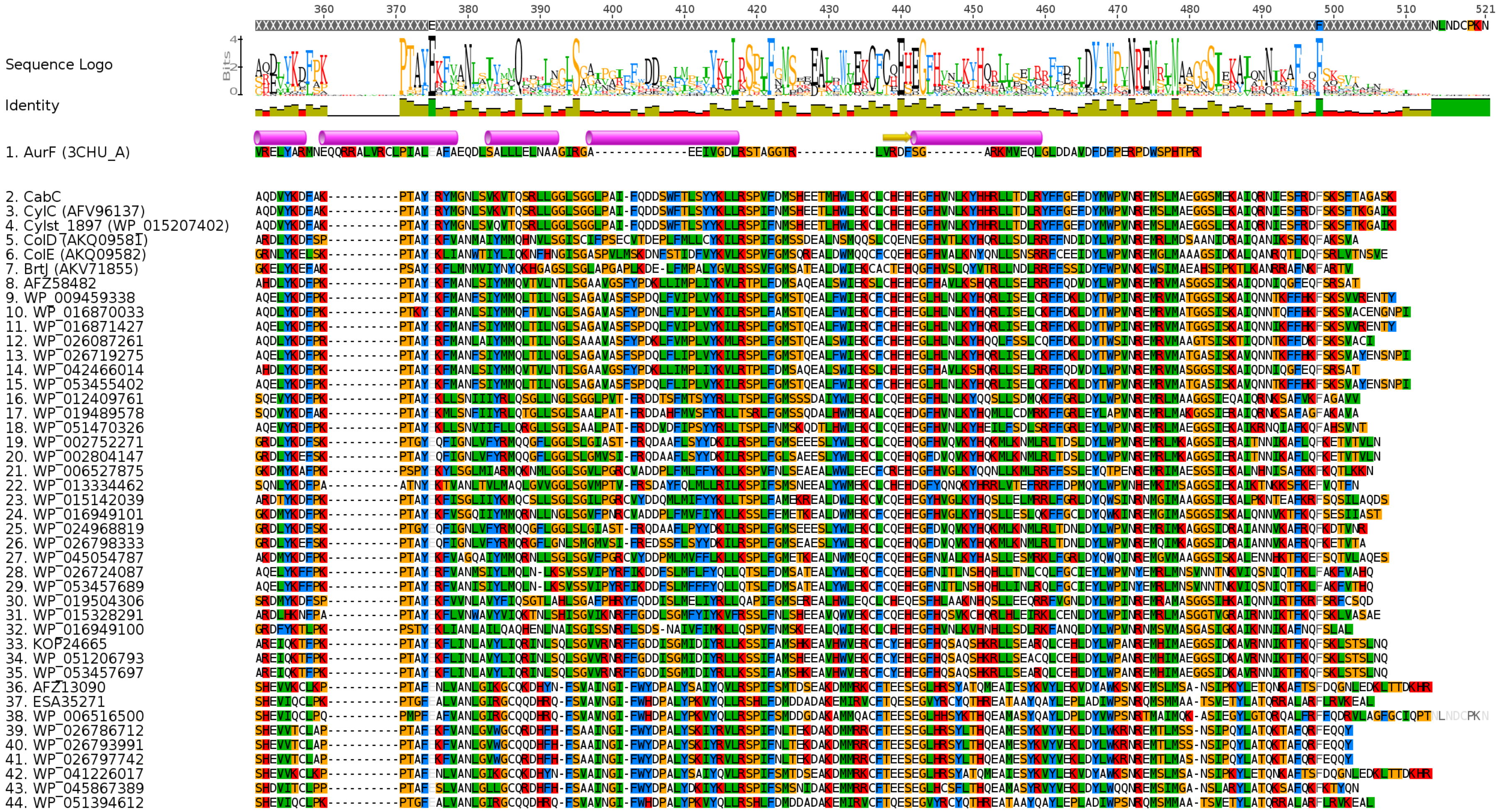


**Figure S3.** Protein sequence alignment of CabC and homologues. The consensus sequence based on a 100% conservation rule along with a sequence logo and an identity graph is depicted at the top. The first sequence shows the *p*-aminobenzoate *N*-oxygenase (AurF) Chain A of *Streptomyces thioluteus*. Conserved glutamic acid and histidine residues responsible for di-iron coordination are depicted below this sequence.

**Table S9.** Oligonucleotide primers used for gap-closure and frameshift refutation.

| Name | Sequence 5′–3′ | Usage [a] | Comment |
|---|---|---|---|
| MP01_129-for | GGAAATTTGAACCACCGAC | A | Gap-closure between contig00129 and contig00638 |
| MP02_129-for_seq | CCTTGAACTTTACTACTAG | S | |
| MP03_638-rev_seq | AGGTATTGGATTACGAATC | S | |
| MP04_638-rev | AGTAGCAATAGCCGCCATC | A | |
| MP05_638-for | ATCTGCTTATGATGTAGCG | A | Gap-closure between contig00638 and contig00697 |
| MP06_638-for_seq | AGCATAGTGAACTGCGACC | S | |
| MP07_697-rev_seq | TCCCTTTGTCTAGCAGGAC | S | |
| MP08_697-rev | GCTAAACCAACAGCATATG | A | |
| MP09_129-for_seq2 | GAGATTCAGCAGGTTCGG | S | Gap-closure between contig00129 and contig00638 |
| SH1_CAVN2-F | CACCCCCTTTAGAAGACCTGG | A | Refutation of two putative frameshifts in 454-based assembly |
| SH2_CAVN2-R | GTGCATCTTCCCAAGCCTCT | A | |
| SH3_CAVN2-F | GTTACAAGCCCCTAAGTTCG | S | |
| SH4_CAVN2-F | CGTCTAGCTGGTGTTAGTGC | S | |
| SH5_CAVN2-F | TCCCAAGAGAATGCAGCCAG | A, S | |
| SH6_CAVN2-R | GCCATCAAGCTCATTTCCCG | A, S | |
| SH7_CAVN2-F | GTAAGGCGCTGCAAGAACTG | A, S | |
| SH8_CAVN2-R | ATCTGGTTTGATGCCCCAGG | A, S | |

[a] Abbreviations: A = amplification, S = sequencing.

**Table S10.** Basic data of biological activity for compounds **1–30** against Gram-positive as well as Gram-negative bacteria and HaCaT cells.[a]

| # | MIC (µg/mL) | | | | | | | | | | | | | | | $IC_{50}$ (µg/mL) |
|---|---|---|---|---|---|---|---|---|---|---|---|---|---|---|---|---|
| | *E. faecium* DSM-20477 | *E. faecium* DSM-17050 (VREF) | *M. tuberculosis* ATCC 25618 (H37Rv) | *S. aureus* Newman (MSSA) | *S. aureus* N315 (MRSA) | *S. aureus* 1 (MRSA)[b] | *S. aureus* Mu50 (MRSA/VISA) | *S. pneumoniae* 7 (ATCC 49619)[b] | *S. pneumoniae* DSM-20566 | *S. pneumoniae* DSM-11865 (PRSP) | *E. coli* 13[b] | *E. coli* TolC-deficient | *E. coli* TolC-deficient + PMBN | *K. pneumoniae* 18 (KRKP)[b] | *P. aeruginosa* 22 (MDR)[b] | HaCaT[b] |
| **1** | 8 | 2–8 | 8–12 | 1 | 0.25–0.5 | 0.08 | 0.5 | 0.2 | 1 | 2 | >50 | >64 | 2 | >50 | >50 | 2.9 |
| **2** | 8 | 4–8 | 2–5 | 0.25 | 0.25–0.5 | n.t. | 0.25–0.5 | n.t. | 1 | 0.25–0.5 | n.t. | >64 | 1–2 | n.t. | n.t. | n.t. |
| **3** | 8 | 8 | 1–1.5 | 0.125–0.25 | 0.125 | 0.63 | 0.125–0.25 | 0.2 | 0.5 | 0.25–0.5 | >50 | >64 | 2 | >50 | >50 | 2.1 |
| **4** | 8 | 4–8 | 0.5–1.5 | 0.5 | 0.25–0.5 | 0.08 | 0.25 | 0.2 | 1 | 0.5 | >50 | >64 | 1 | >50 | >50 | 3.5 |
| **5** | 32 | 16–32 | 2–4 | 0.25 | 0.25 | 0.16 | 0.125 | 0.2 | 1 | 0.5–2 | >50 | >64 | 8–16 | >50 | >50 | 7.4 |
| **6** | n.t. | 8–16 | 4–8 | n.t. | 0.5 | n.t. | n.t. | n.t. | n.t. | 1 | n.t. | n.t. | n.t. | n.t. | n.t. | n.t. |
| **7** | 2 | 2 | 0.5–1.5 | 0.125 | 0.06–0.125 | 0.04 | 0.125 | 0.2 | 0.25 | 0.25 | >50 | >64 | 1 | >50 | >50 | 2.4 |
| **8** | 4 | 4 | 2–5 | 0.25 | 0.125–0.25 | n.t. | 0.25 | n.t. | 0.5–1 | 0.5 | n.t. | >64 | 1 | n.t. | n.t. | n.t. |
| **9** | 8 | 4 | 0.5–2 | 0.125–0.25 | 0.125 | 0.16 | 0.5 | 0.2 | 0.25–0.5 | 0.25 | >50 | >64 | 1 | >50 | >50 | 7.5 |
| **10** | 4 | 4 | 2–3 | 0.125–0.25 | 0.125 | 0.08 | 0.125–0.25 | 0.2 | 0.5–1 | 0.25–0.5 | >50 | >64 | 1–2 | >50 | >50 | 4.0 |
| **11** | n.t. | 8 | 2–4 | n.t. | 0.25 | 0.08 | n.t. | 0.2 | n.t. | 0.25 | >50 | n.t. | n.t. | >50 | >50 | 2.2 |
| **12** | 4 | 2–4 | 2–4 | 0.125–0.25 | 0.125 | 0.08 | 0.125 | 0.2 | 0.25–0.5 | 0.25 | >50 | >64 | 1 | >50 | >50 | 2.1 |
| **13** | 4 | 4 | 1–1.5 | 0.125–0.25 | 0.125 | 0.08 | 0.125 | 0.2 | 0.25–0.5 | 0.125–0.25 | >50 | >64 | 1 | >50 | >50 | 3.4 |
| **14** | 4 | 4 | 32–64 | 0.25 | 0.125 | 0.08 | 0.125–0.5 | 0.2 | 0.25–0.5 | 0.25–0.5 | >50 | >64 | 1–2 | >50 | >50 | 3.9 |
| **15** | n.t. | 4 | 2–3 | n.t. | 0.25 | 0.08 | n.t. | 0.2 | n.t. | 0.5 | >50 | n.t. | n.t. | >50 | >50 | 3.8 |
| **16** | 2 | 2 | 1.5–2 | 0.125–0.25 | 0.06–0.125 | 0.08 | 0.125 | 0.2 | 0.25 | 0.5 | >50 | >64 | 0.5–1 | >50 | >50 | 2.7 |
| **17** | 2 | 2 | 2–5 | 0.125–0.25 | 0.06–0.125 | 0.08 | 0.125 | 0.2 | 0.25 | 0.25 | >50 | >64 | 0.5 | >50 | >50 | 3.4 |
| **18** | 2–4 | 2 | 1–1.5 | 0.125 | 0.06 | 0.08 | 0.125 | 0.2 | 0.25 | 0.25 | >50 | >64 | 0.5–1 | >50 | >50 | 3.6 |
| **19** | 4–8 | 8 | 2–3 | 0.125 | 0.125 | 0.08 | 0.125 | 0.2 | 0.5 | 0.25 | >50 | >64 | 1–4 | >50 | >50 | 2.5 |
| **20** | n.t. | 2 | 1.5–2 | n.t. | 0.125–0.25 | 0.08 | n.t. | 0.2 | n.t. | 0.25 | >50 | n.t. | n.t. | >50 | >50 | 4.8 |
| **21** | n.t. | 8 | >8 | 1–2 | 0.5 | 0.63 | 8 | 1.3 | n.t. | 2 | >50 | >64 | 4–8 | >50 | >50 | 7.0 |
| **22** | n.t. | n.t. | >8 | n.t. | n.t. | 0.63 | n.t. | 1.3 | n.t. | n.t. | >50 | n.t. | n.t. | >50 | >50 | 7.5 |
| **23** | n.t. | 4–8 | 2–4 | 0.5 | 0.5 | 0.31 | 1 | 0.63 | n.t. | n.t. | >50 | >64 | 2 | >50 | >50 | 5.9 |
| **24** | n.t. | n.t. | >8 | n.t. | n.t. | 0.31 | n.t. | 0.63 | n.t. | n.t. | >50 | n.t. | n.t. | >50 | >50 | 6.7 |
| **25** | 4 | 2 | 2–4 | 0.125 | 0.125 | 0.26 | 0.125 | 0.57 | 0.25 | 0.25 | >50 | >64 | 1–2 | >50 | >50 | 2.9 |
| **26** | 4 | 2 | 0.5–1 | 0.125 | 0.125 | 0.08 | 0.125 | 0.2 | 0.5 | 0.125 | >50 | >64 | 2–4 | >50 | >50 | 1.8 |
| **27** | 8 | 2 | 1–2 | 1–4 | 0.5–2 | 0.63 | 2–8 | 1.6 | 2–8 | 4–8 | >50 | >64 | 64 | >50 | >50 | 7.3 |
| **28** | 16 | 16 | >16 | 4 | 4–8 | 3.2 | 4–8 | 6.23 | 4 | 4 | >50 | >64 | 4 | >50 | >50 | 37.3 |
| **29** | 64 | 64 | >16 | 2–4 | 4 | >50 | 4–8 | >50 | 2–8 | 2 | >50 | >64 | 8–32 | >50 | >50 | 16.5 |
| **30** | >64 | >64 | >16 | 4–8 | >64 | >50 | >64 | >50 | 4 | 2–4 | >50 | >64 | >64 | >50 | >50 | 17.5 |
| **POS** | 2.0 [c] | >64 [c] | 0.024–0.036 [d]<br>0.125–0.25 [e]<br>0.02–0.08 [f]<br>0.2–0.8 [g] | 0.5 [c] | 1.0 [c] | 2.0 [c,h] | 16 [c] | 2.0 [c,h] | <0.03 [i] | >64 [i] | 0.0062 [j, k,l]<br>62.5 [c] | 0.003 [j] | 0.003 [j] | 1.25 [j]<br>0.62 [k] | 0.025 [l]<br>250 [c] | 3.9 [m] |

[a] Abbreviations: VREF = vancomycin-resistant *E. faecium*, MSSA = methicillin-sensitive *S. aureus*, MRSA = methicillin-resistant *S. aureus*, VISA = vancomycin-intermediate *S. aureus*, PRSP = penicillin-resistant *S. pneumoniae*, PMBN = polymyxin B nonapeptide, KRKP = kanamycin-resistant *K. pneumoniae*, MDR = multi-drug resistant (for detailed resistance profile, see Pretsch *et al*. [4]), n.t. = not tested, POS = positive control; [b] Equivalent data for **10–30** have previously been reported; For further details, see Preisitsch *et al*. [5] (**10–25**) and Preisitsch *et al*. [6] (**26–30**); [c] vancomycin; [d] delamanid; [e] pretomanid (formerly known as PA-824); [f] isoniazid; [g] rifampicin; [h] fusidic acid; [i] ampicillin; [j] ciprofloxacin; [k] moxifloxacin; [l] levofloxacin; [m] mitoxantrone.

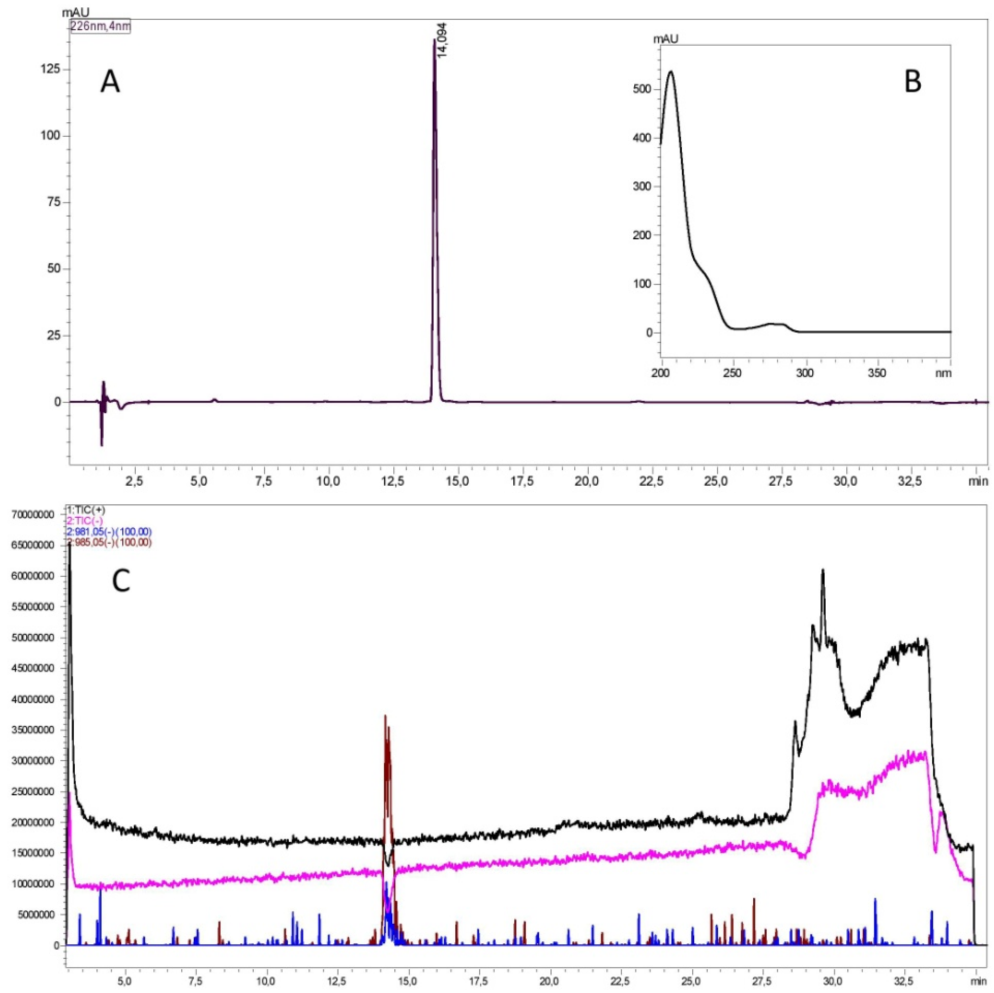


**Figure S4.** *Cont.*

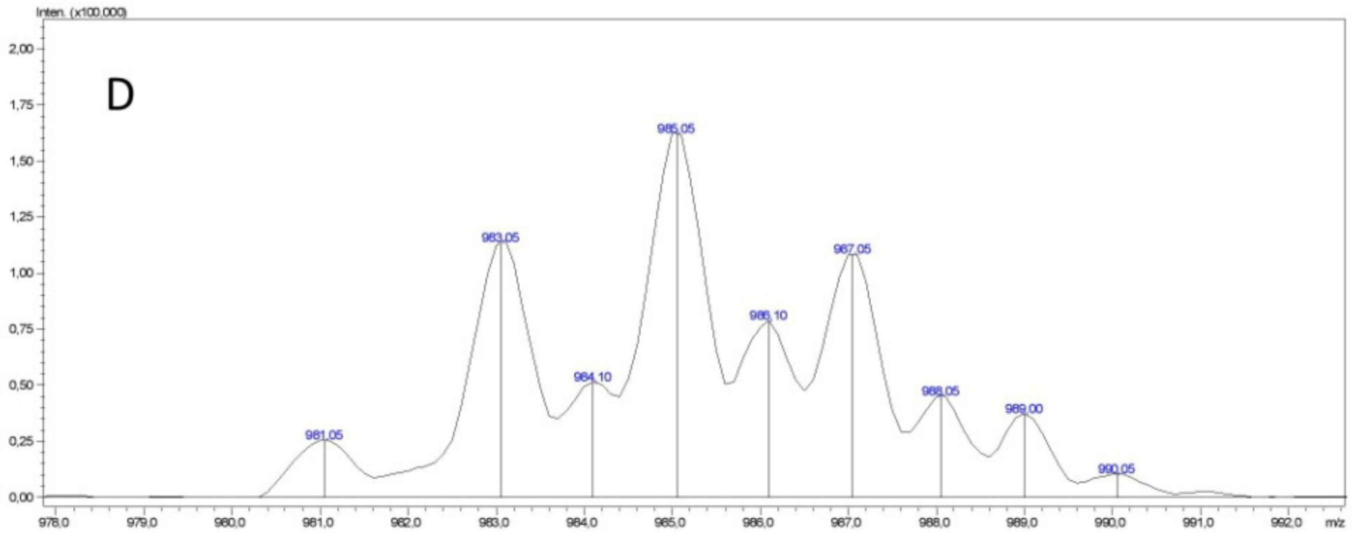


**Figure S4.** Identity and stability control of carbamidocyclophane Q (**5**) after the antimycobacterial bioactivity assay. HPLC-UV-MS analysis was performed on a Shimadzu LC-20A Prominence liquid chromatography system with a SPD-M20A diode array detector (DAD) coupled to a Shimadzu LCMS-8030 triple quadrupole (QqQ) mass spectrometer using a Phenomenex Kinetex PFP column (100 × 4.6 mm, 2.6 µm, 100 Å) and a binary gradient of MeOH in deionized $H_2O$ with a flow rate of 0.8 mL/min from 60% to 80.3% MeOH in 26 min at 40 °C. Sample: 0.1 mg of **5** in 1.0 mL MeOH, 10 µL injection, solvent flow split of 10:1 after the DAD analysis and prior to QqQ measurement. (**A**) HPLC chromatogram of **5** detected at wavelength $\lambda$ = 226 nm; (**B**) Online UV spectrum ($\lambda$ from 200 to 400 nm) of **5** at retention time 14.1 min; (**C**) Total ion chromatograms (TICs) of **5** (positive mode in black and negative mode in magenta) and its extracted ion chromatograms (EICs) for *m*/*z* 981.05 and 985.05 (both negative mode), consistent with the monoisotopic mass peak $[M - H]^-$ and the most abundant isotopic mass peak $[M + 4 - H]^-$, respectively; (**D**) Measured isotopic distribution pattern of **5** referring to the $[M - H]^-$ ion.

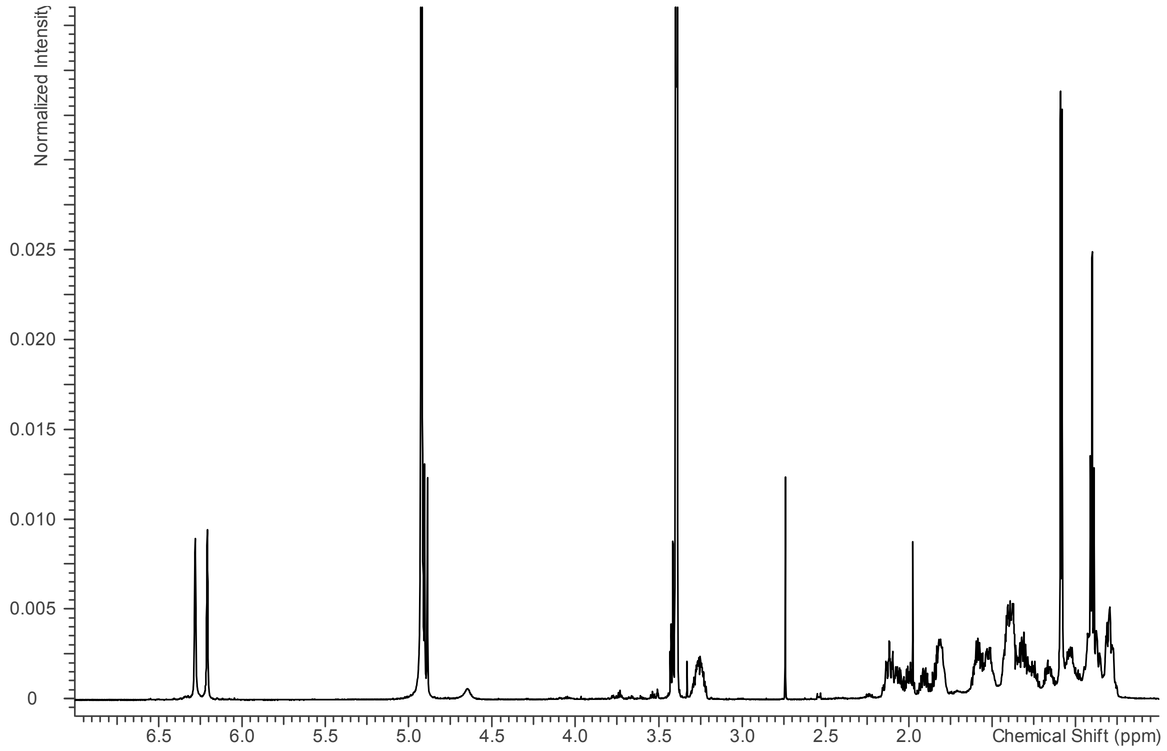


**Figure S5.** $^{1}$H NMR spectrum (600 MHz, MeOH-$d_4$) of carbamidocyclophane M (**1**).

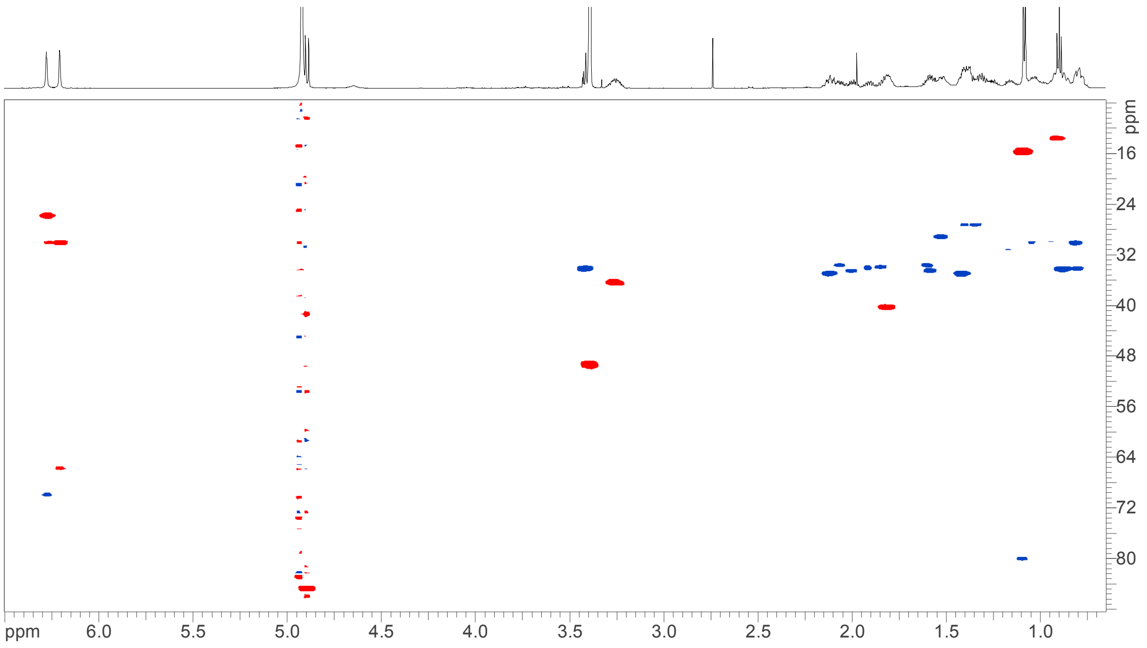


**Figure S6.** HMQC-DEPT spectrum (600 MHz, MeOH-$d_4$) of carbamidocyclophane M (**1**). Red signals are attributed to CH or $CH_3$ groups (positively phased) and blue signals to $CH_2$ groups (negatively phased).

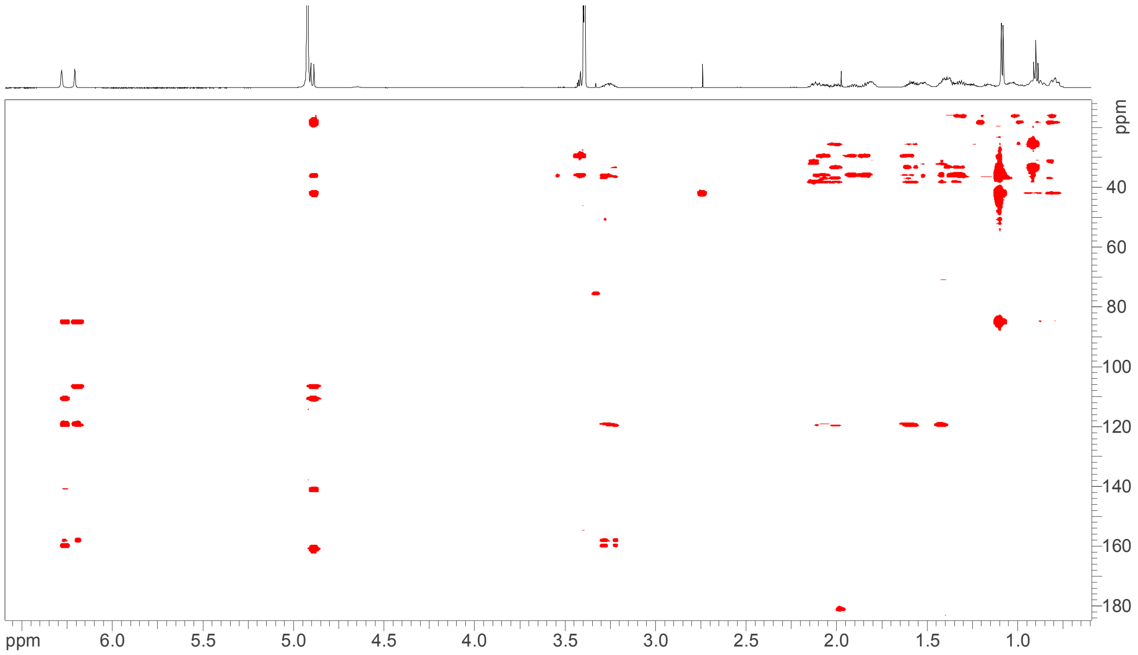


**Figure S7.** HMBC spectrum (600 MHz, MeOH-$d_4$) of carbamidocyclophane M (**1**).

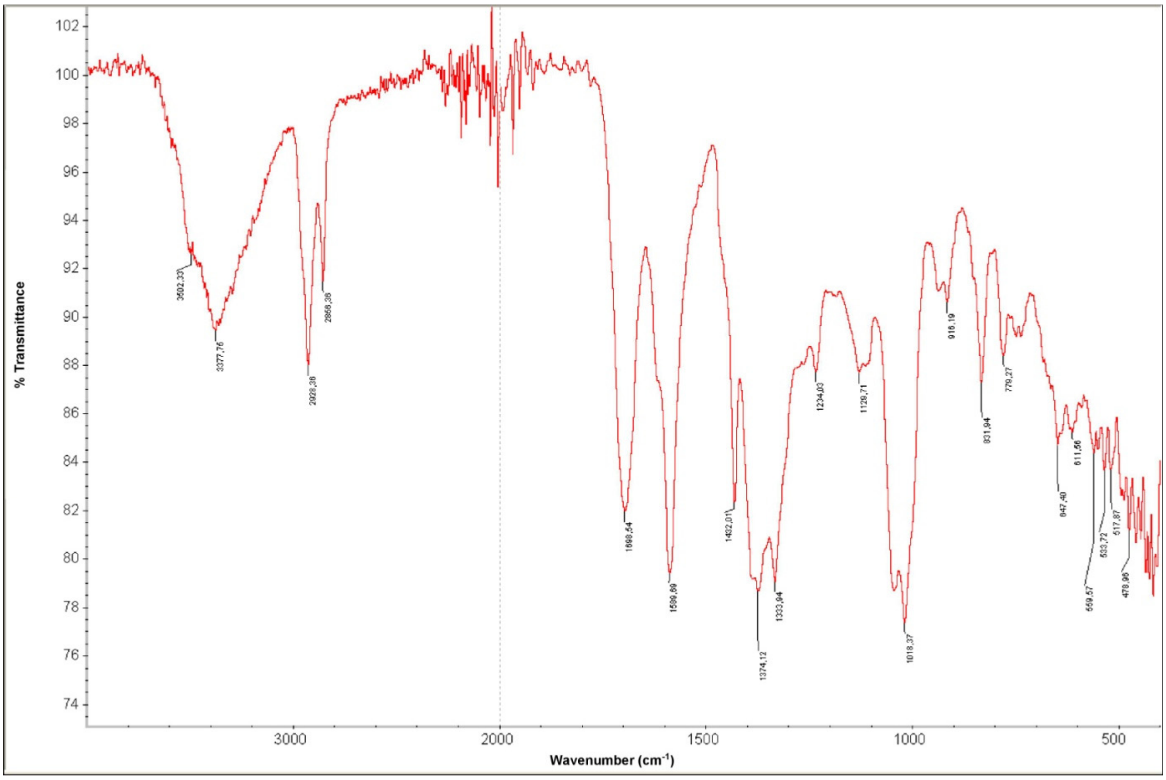


**Figure S8.** ATR-IR (film) spectrum of carbamidocyclophane M (**1**).

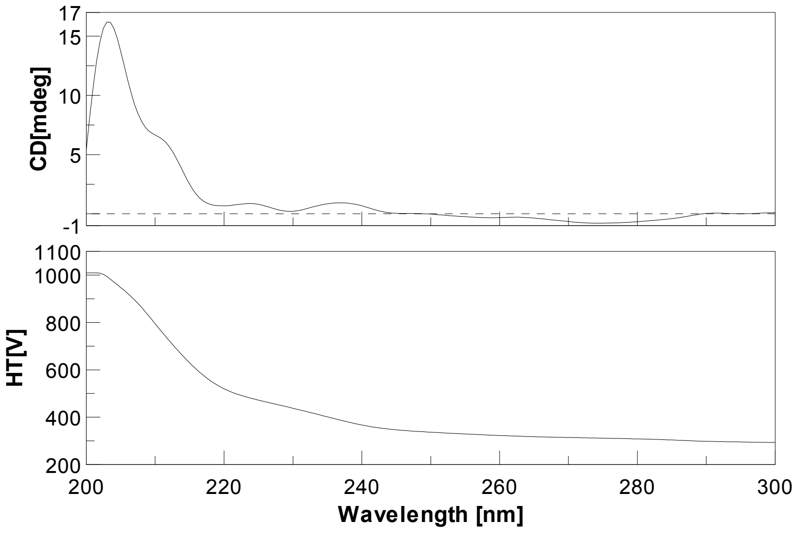


**Figure S9.** ECD spectrum of carbamidocyclophane M (**1**).

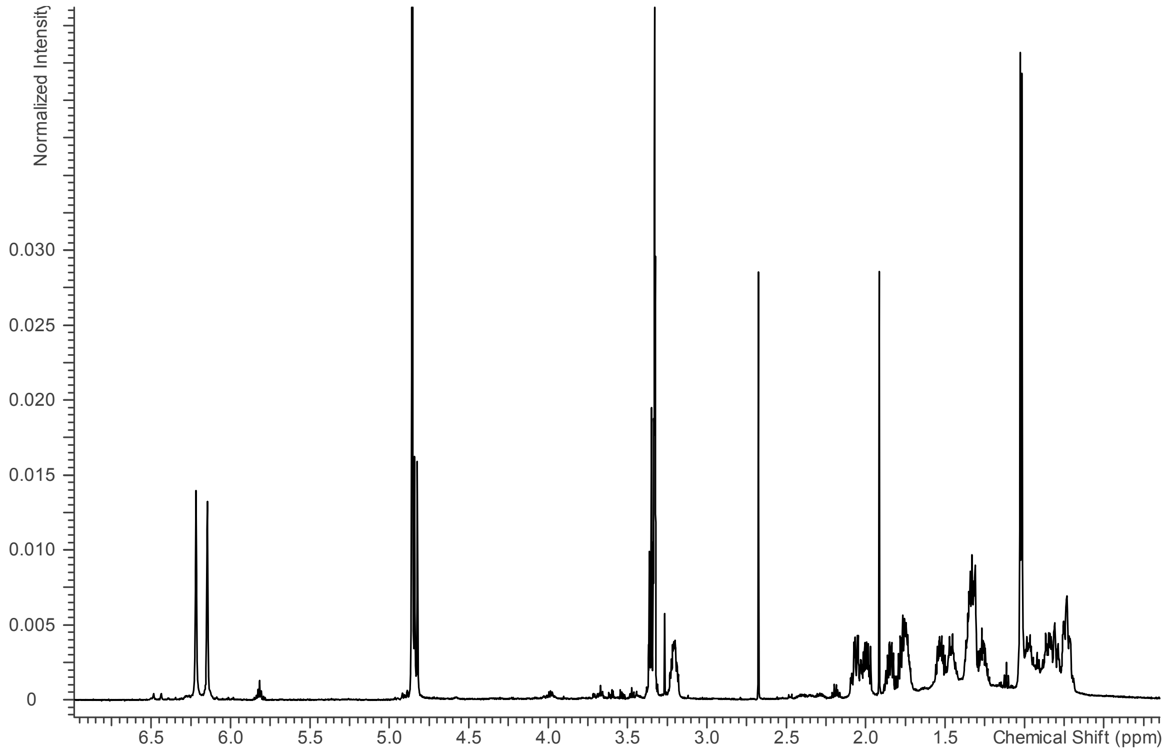


**Figure S10.** $^{1}$H NMR spectrum (600 MHz, MeOH-$d_4$) of carbamidocyclophane N (**2**).

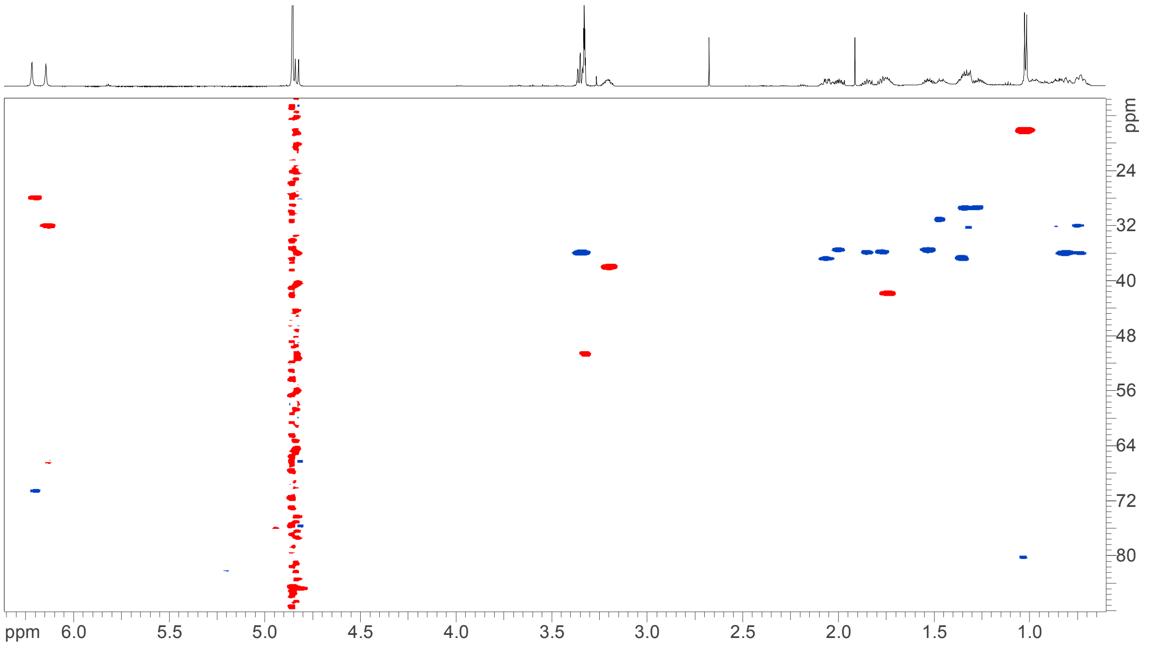


**Figure S11.** HMQC-DEPT spectrum (600 MHz, MeOH-*d*4) of carbamidocyclophane N (**2**). Red signals are attributed to CH or $CH_3$ groups (positively phased) and blue signals to $CH_2$ groups (negatively phased).

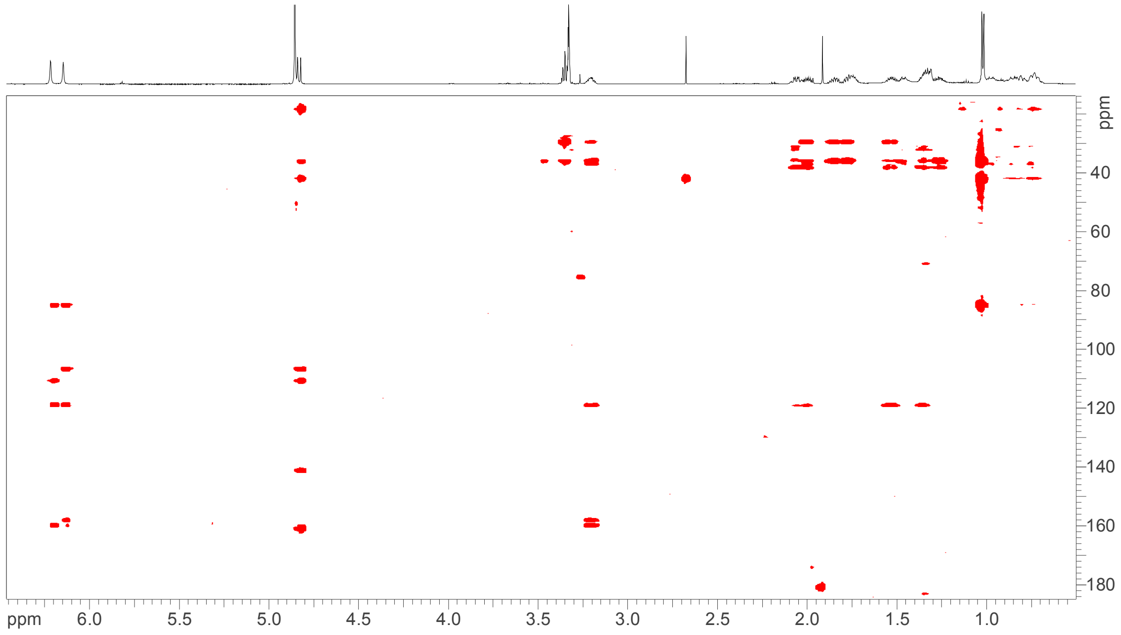


**Figure S12.** HMBC spectrum (600 MHz, MeOH-*d*4) of carbamidocyclophane N (**2**).

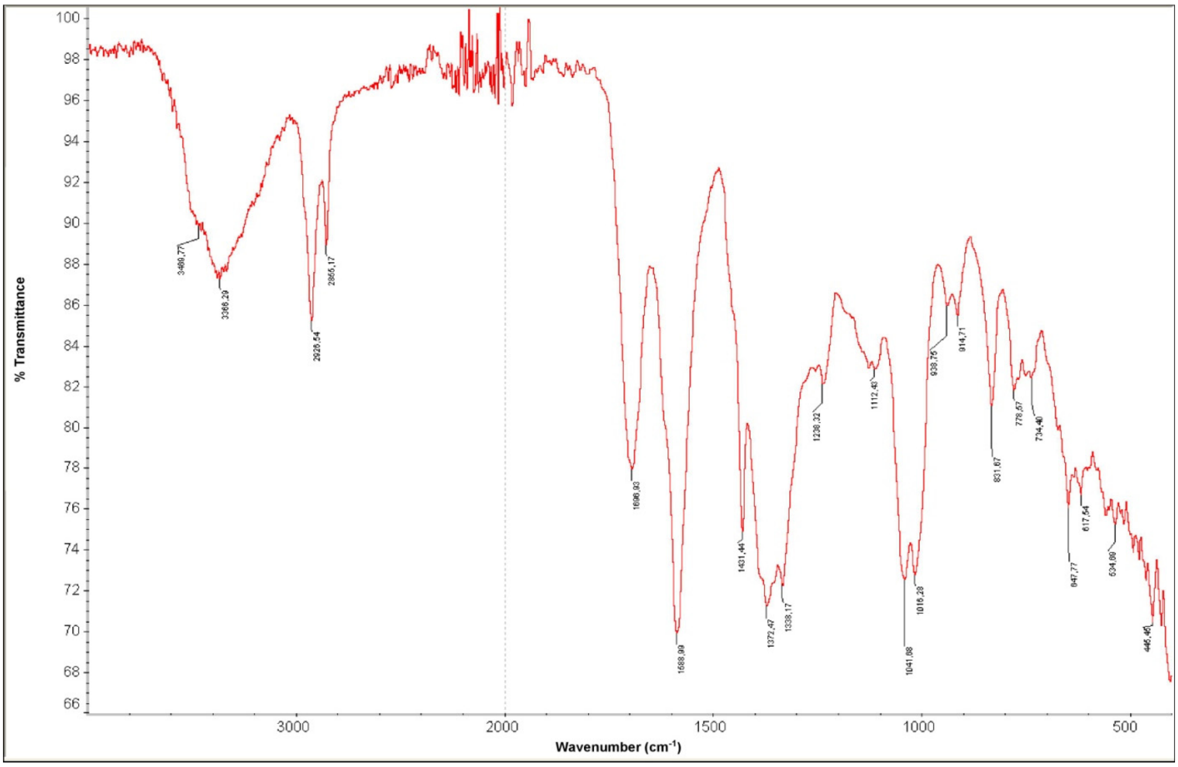


**Figure S13.** ATR-IR (film) spectrum of carbamidocyclophane N (**2**).

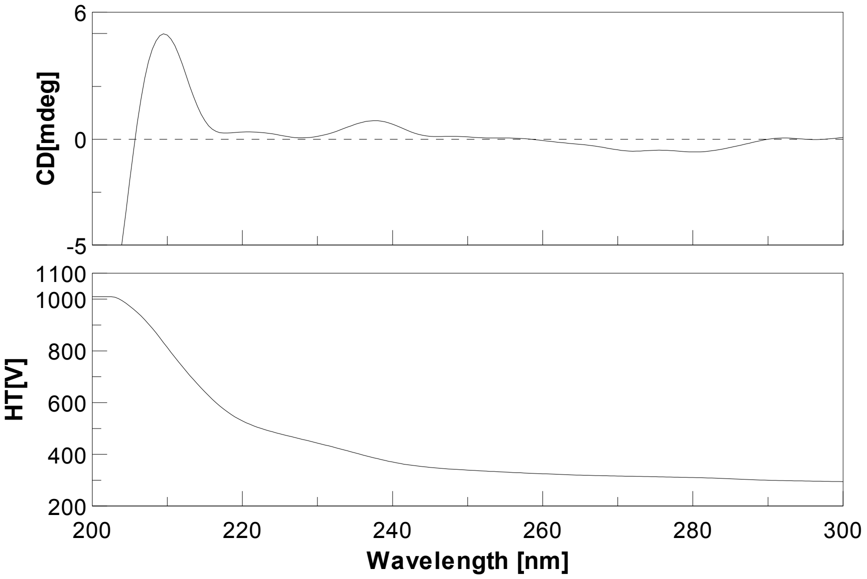


**Figure S14.** ECD spectrum of carbamidocyclophane N (**2**).

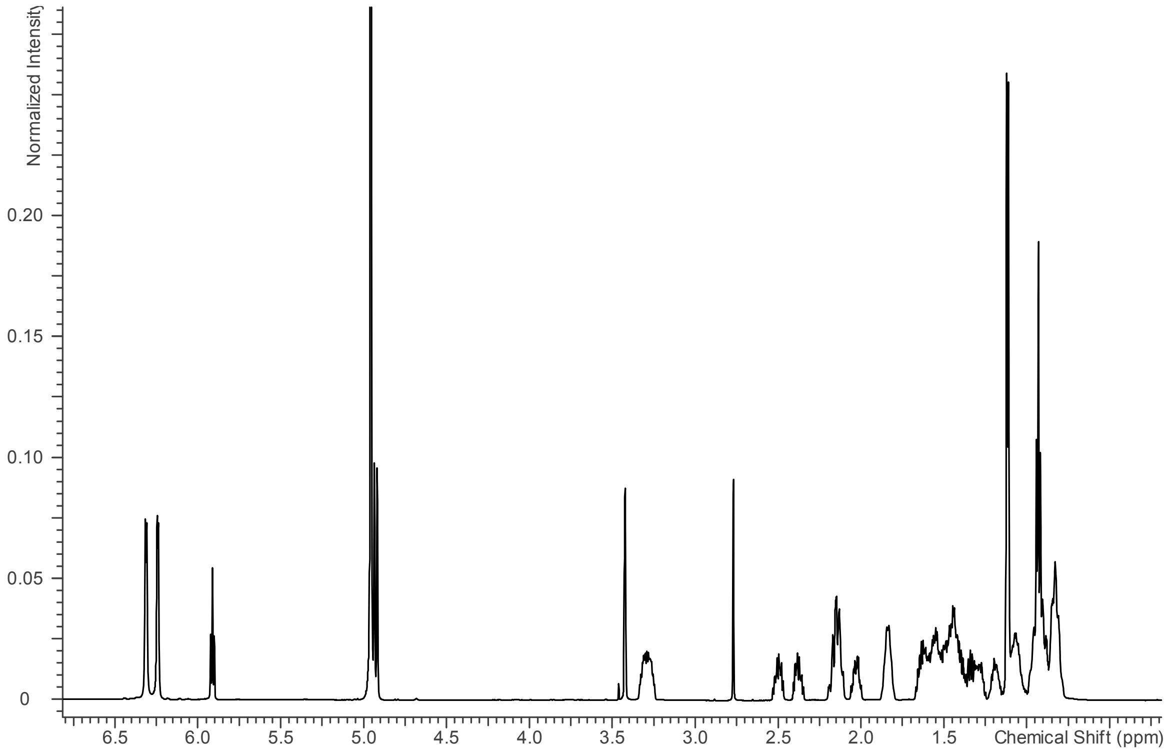


**Figure S15.** $^{1}$H NMR spectrum (600 MHz, MeOH-*d*4) of carbamidocyclophane O (**3**).

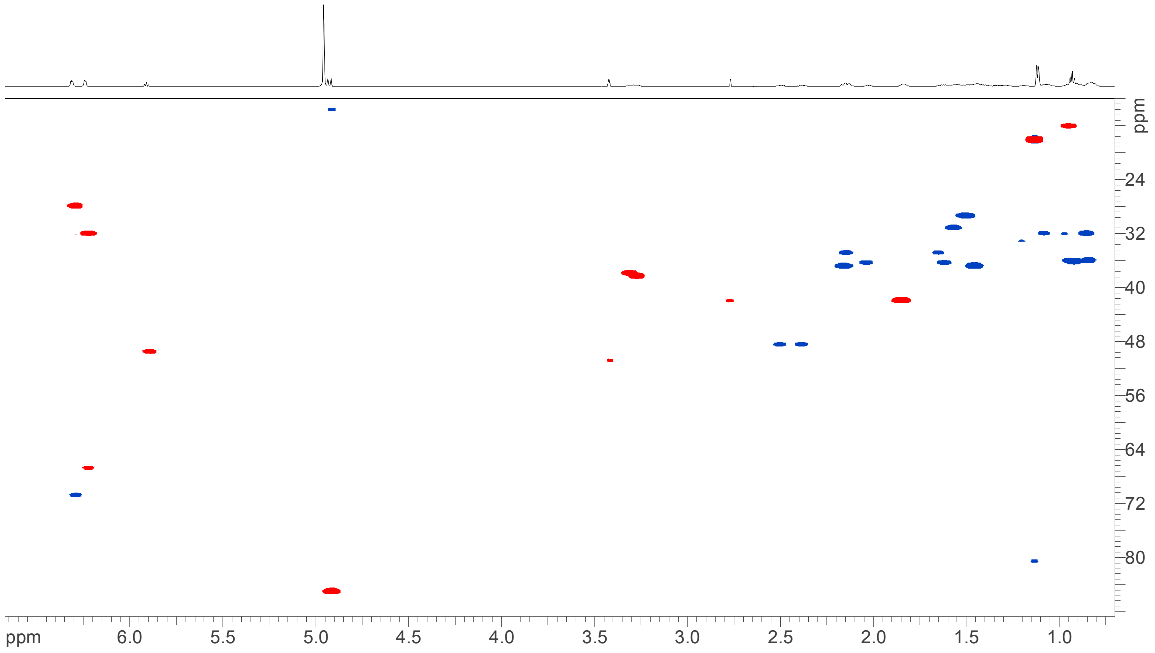


**Figure S16.** HMQC-DEPT spectrum (600 MHz, MeOH-*d*4) of carbamidocyclophane O (**3**). Red signals are attributed to CH or $CH_3$ groups (positively phased) and blue signals to $CH_2$ groups (negatively phased).

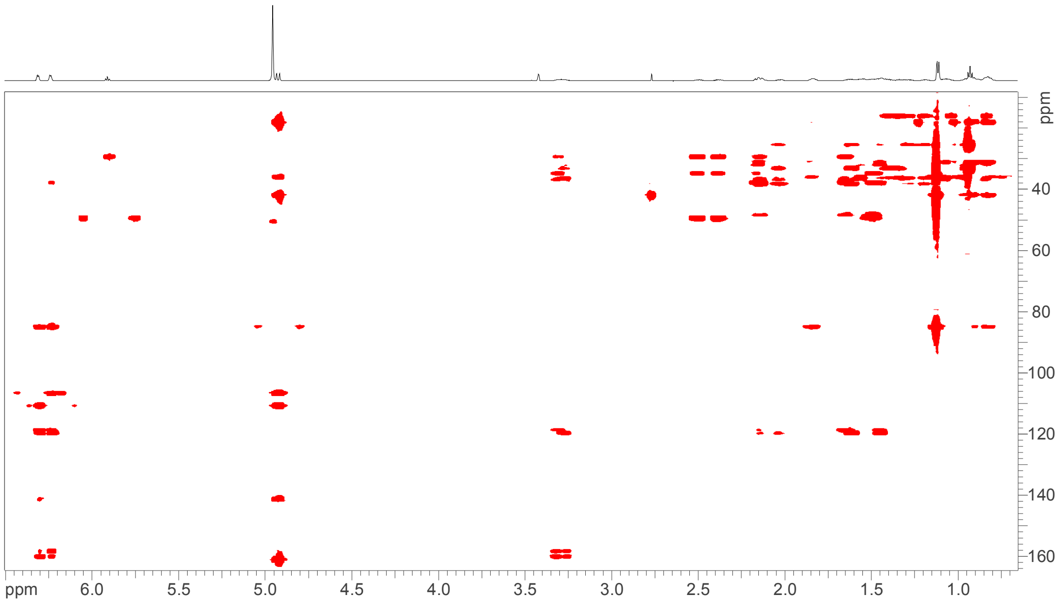


**Figure S17.** HMBC spectrum (600 MHz, MeOH-$d_4$) of carbamidocyclophane O (**3**).

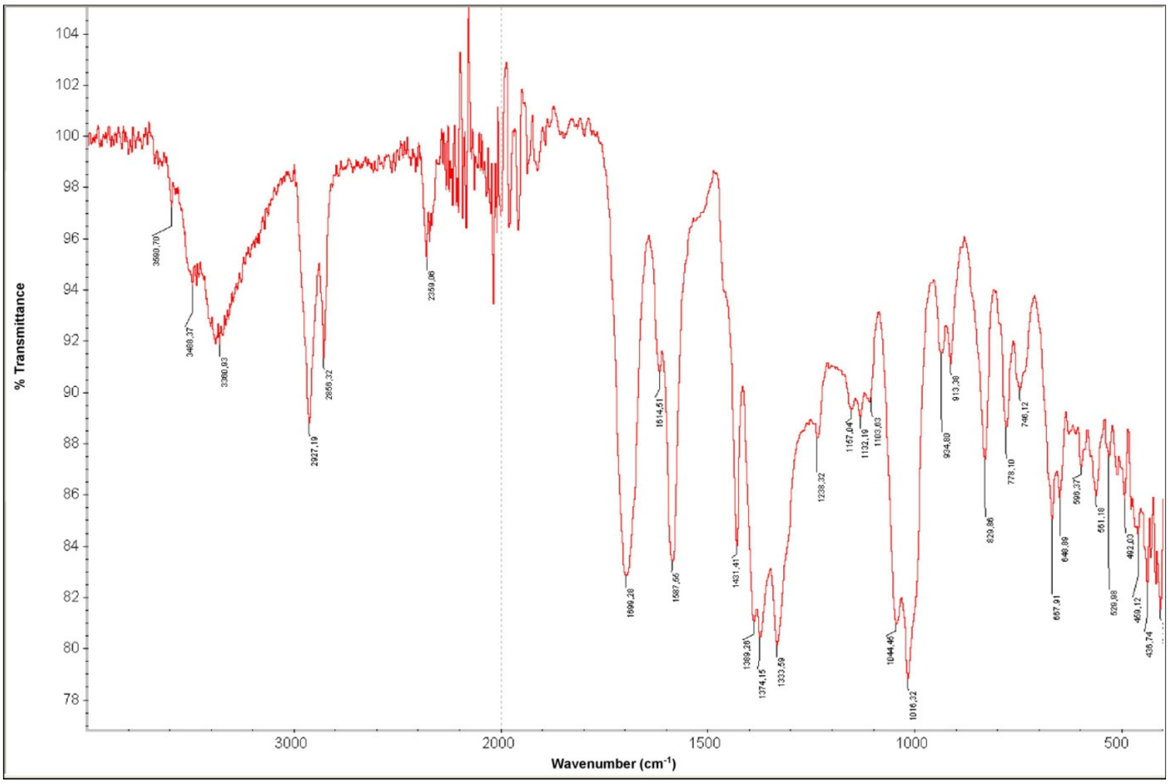


**Figure S18.** ATR-IR (film) spectrum of carbamidocyclophane O (**3**).

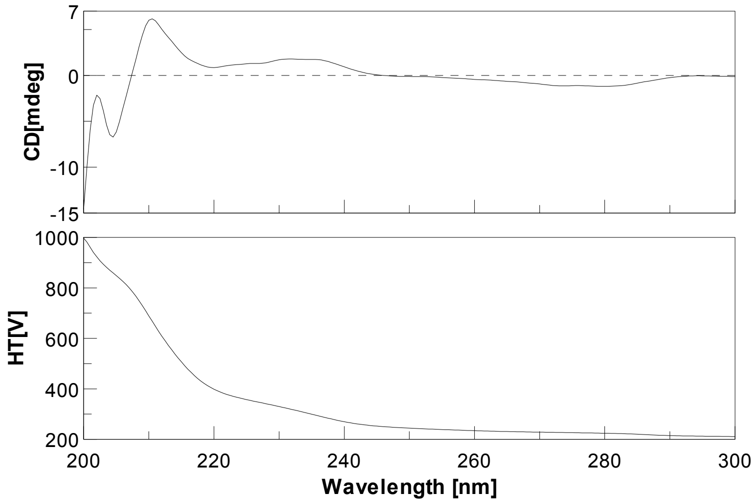


**Figure S19.** ECD spectrum of carbamidocyclophane O (**3**).

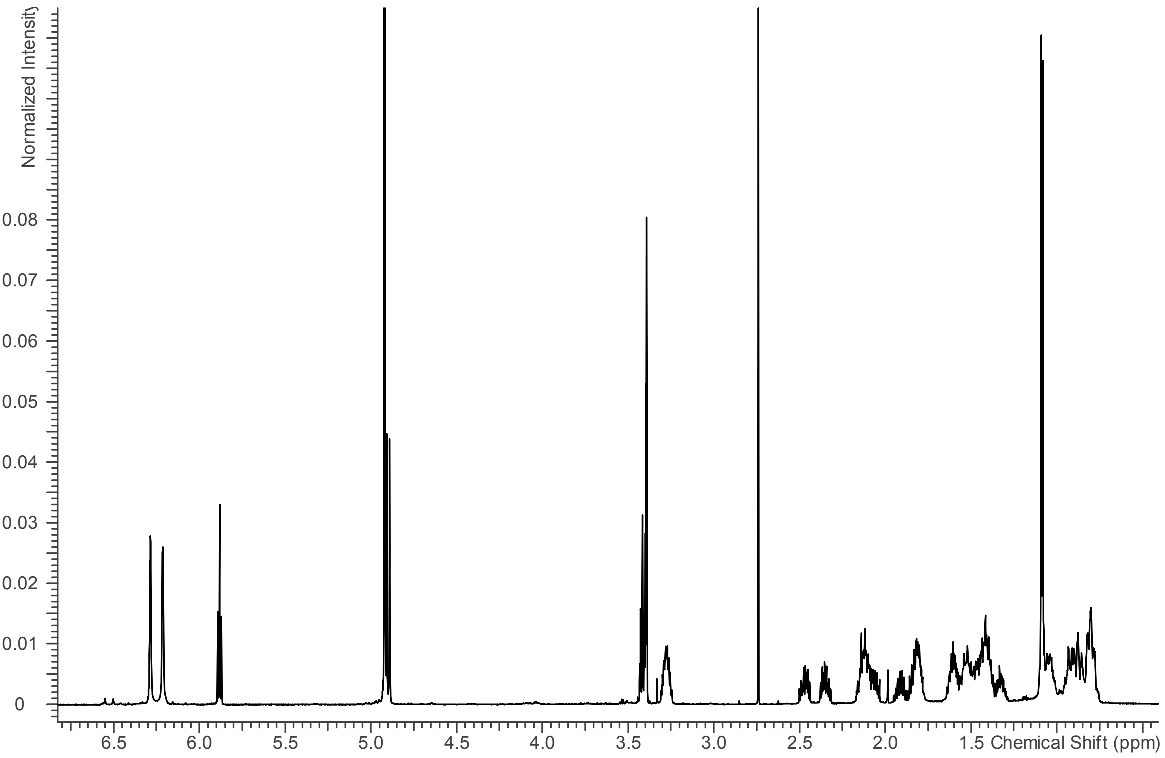


**Figure S20.** $^{1}$H NMR spectrum (600 MHz, MeOH-$d_4$) of carbamidocyclophane P (**4**).

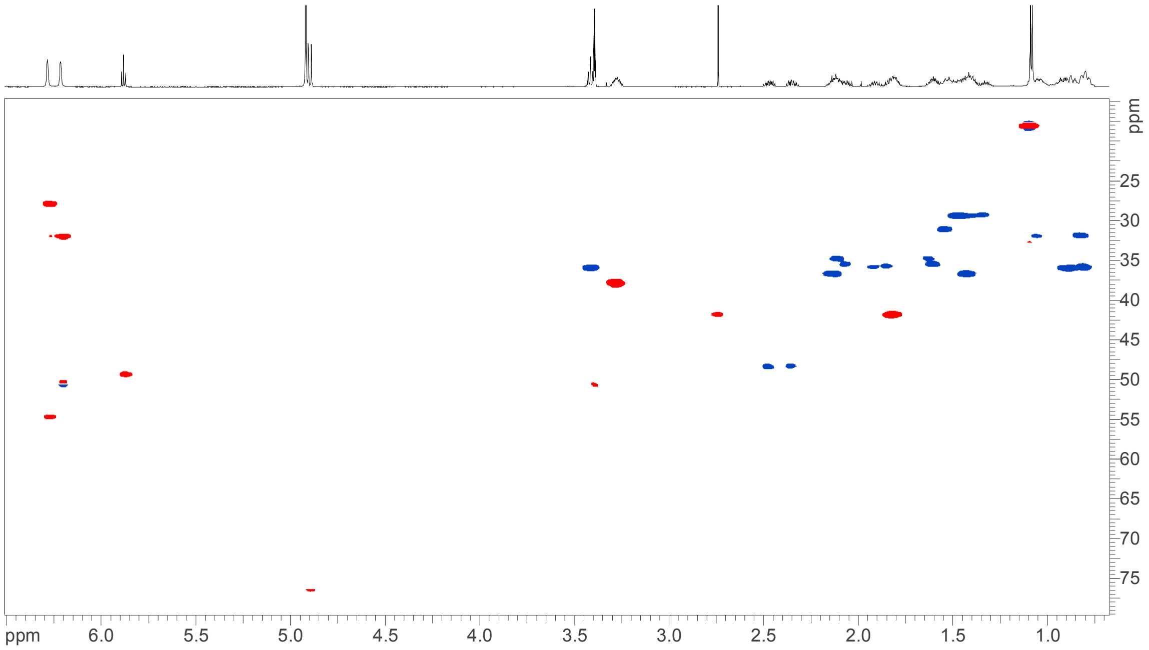


**Figure S21.** HMQC-DEPT spectrum (600 MHz, MeOH-*d*4) of carbamidocyclophane P (**4**). Red signals are attributed to CH or $CH_3$ groups (positively phased) and blue signals to $CH_2$ groups (negatively phased).

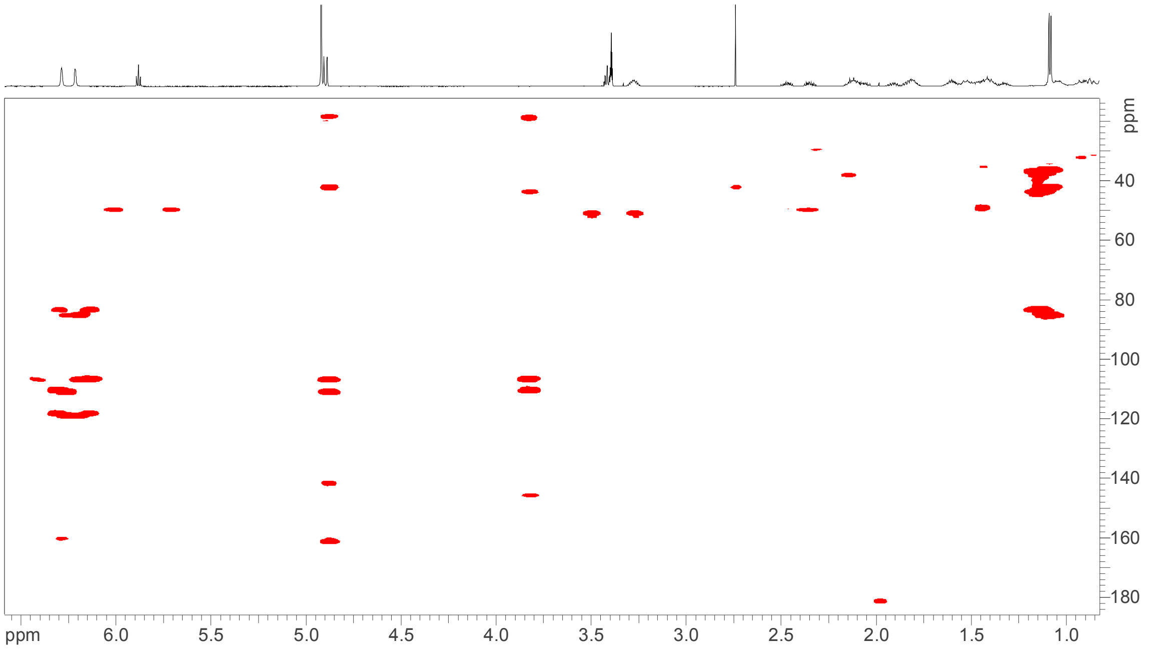


**Figure S22.** HMBC spectrum (600 MHz, MeOH-*d*4) of carbamidocyclophane P (**4**).

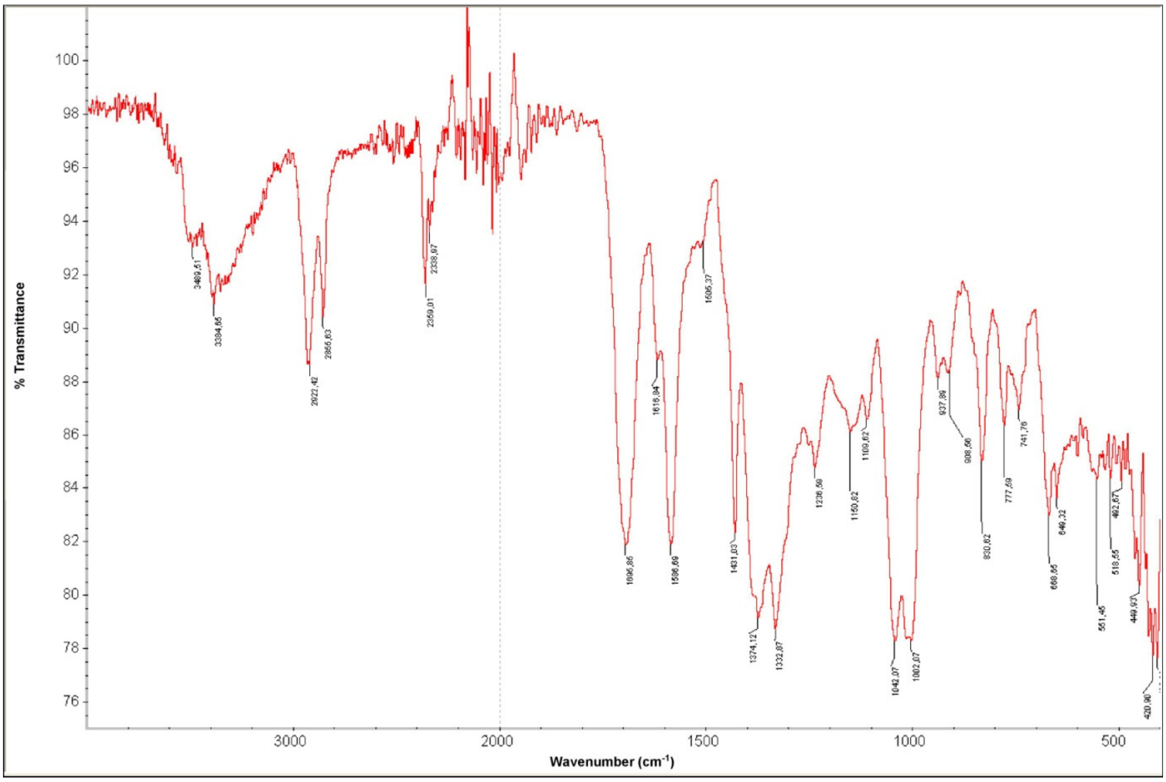


**Figure S23.** ATR-IR (film) spectrum of carbamidocyclophane P (**4**).

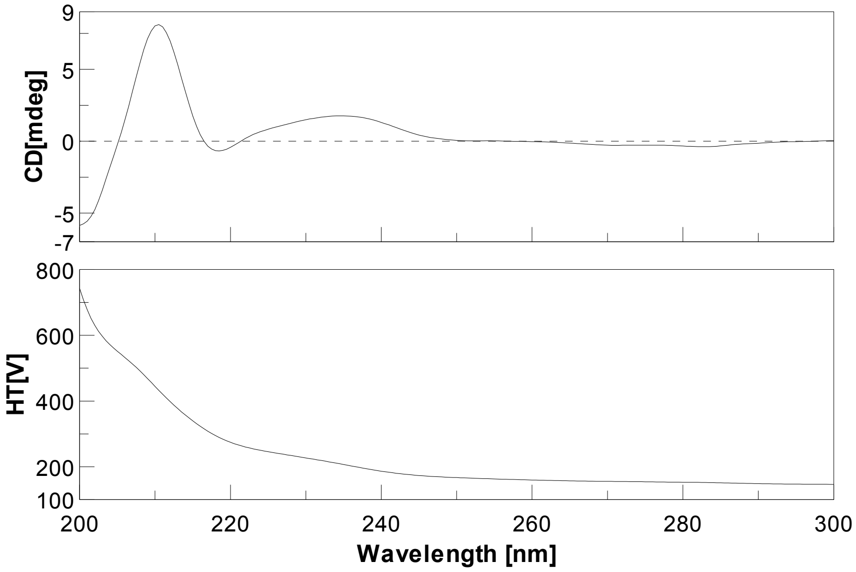


**Figure S24.** ECD spectrum of carbamidocyclophane P (**4**).

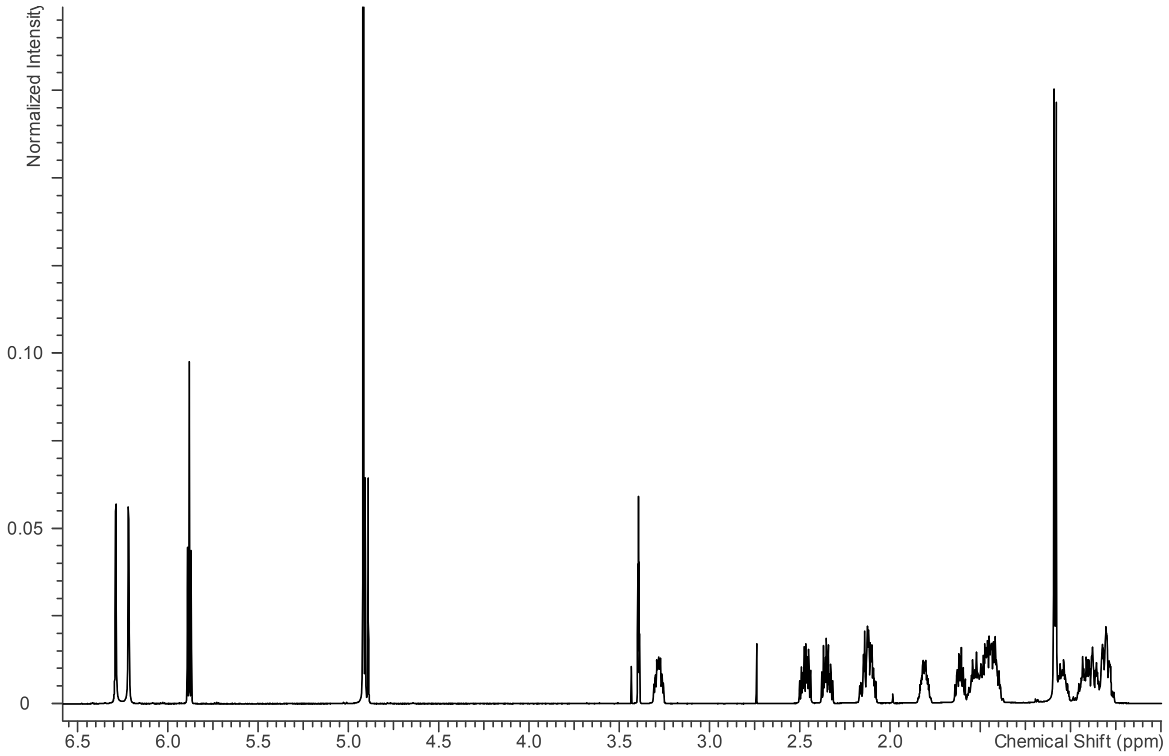


**Figure S25.** $^{1}H$ NMR spectrum (600 MHz, MeOH-$d_4$) of carbamidocyclophane Q (**5**).

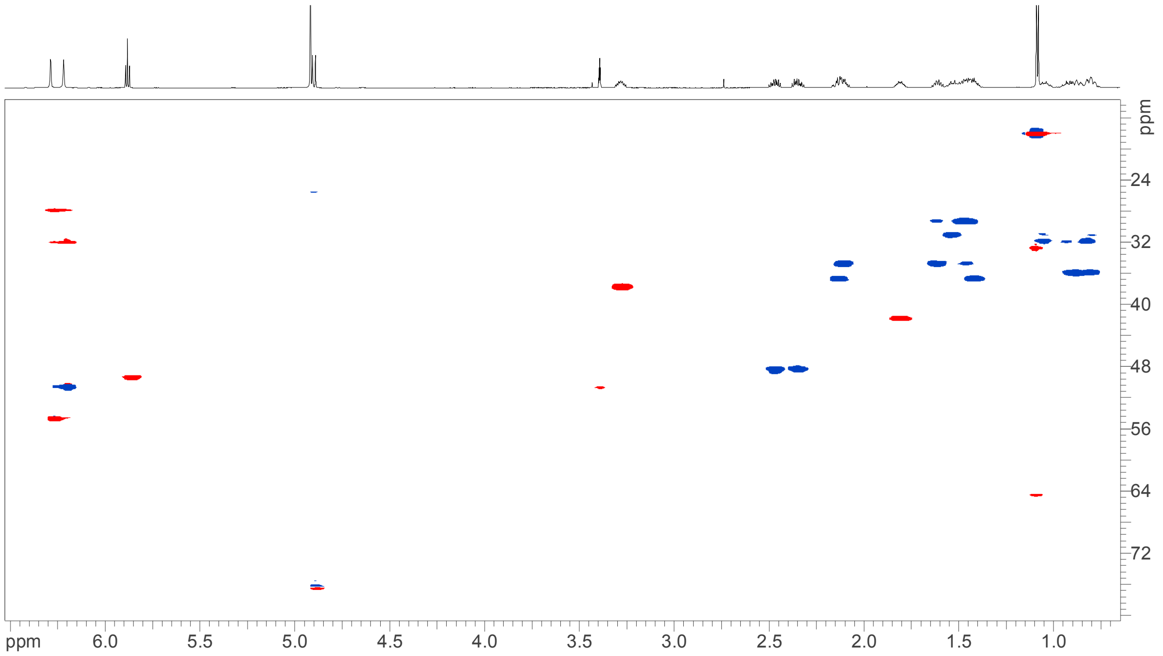


**Figure S26.** HMQC-DEPT spectrum (600 MHz, MeOH-$d_4$) of carbamidocyclophane Q (**5**). Red signals are attributed to CH or $CH_3$ groups (positively phased) and blue signals to $CH_2$ groups (negatively phased).

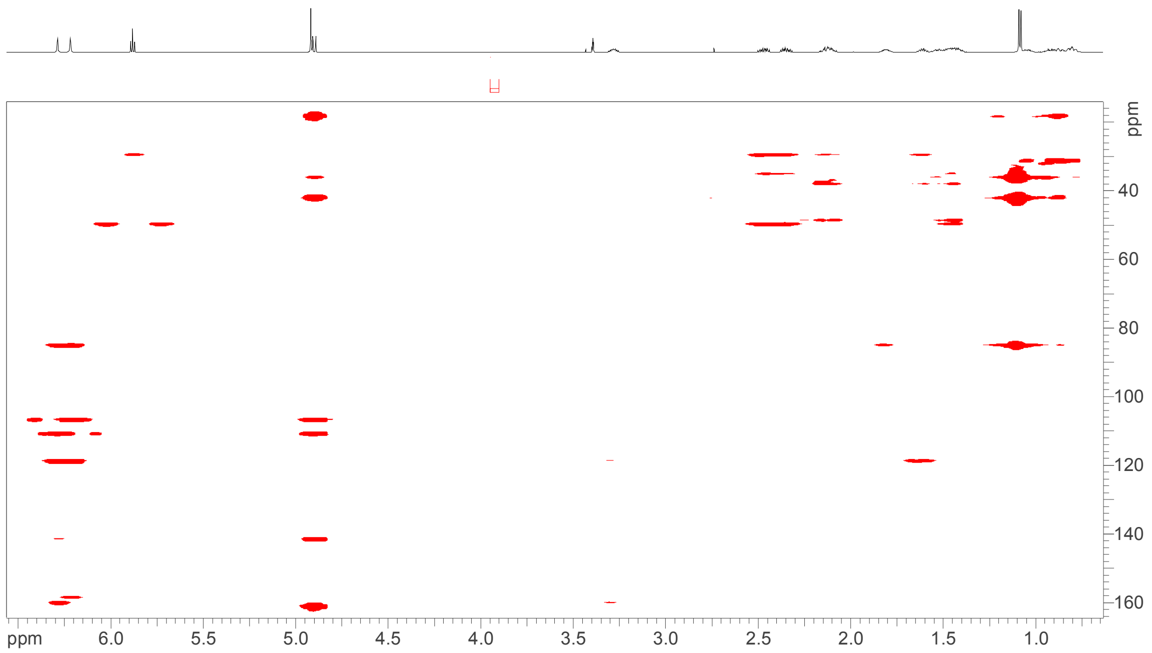


**Figure S27.** HMBC spectrum (600 MHz, MeOH-*d*4) of carbamidocyclophane Q (**5**).

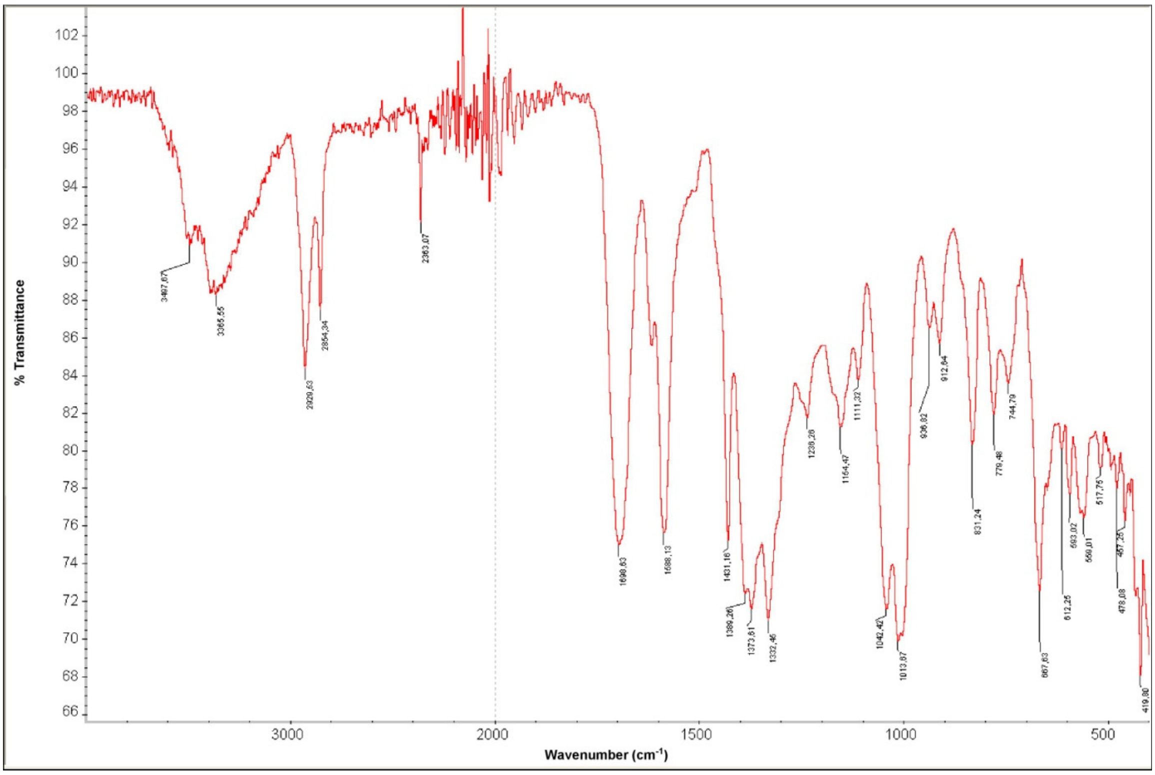


**Figure S28.** ATR-IR (film) spectrum of carbamidocyclophane Q (**5**).

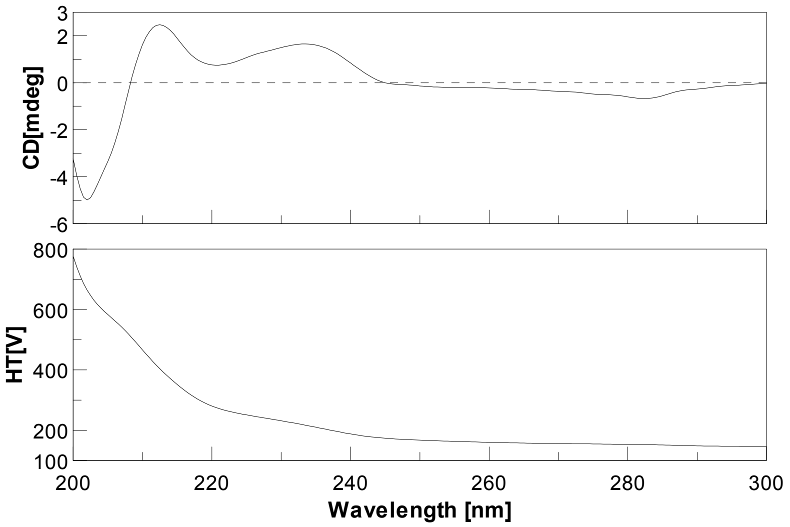


**Figure S29**. ECD spectrum of carbamidocyclophane Q (**5**).

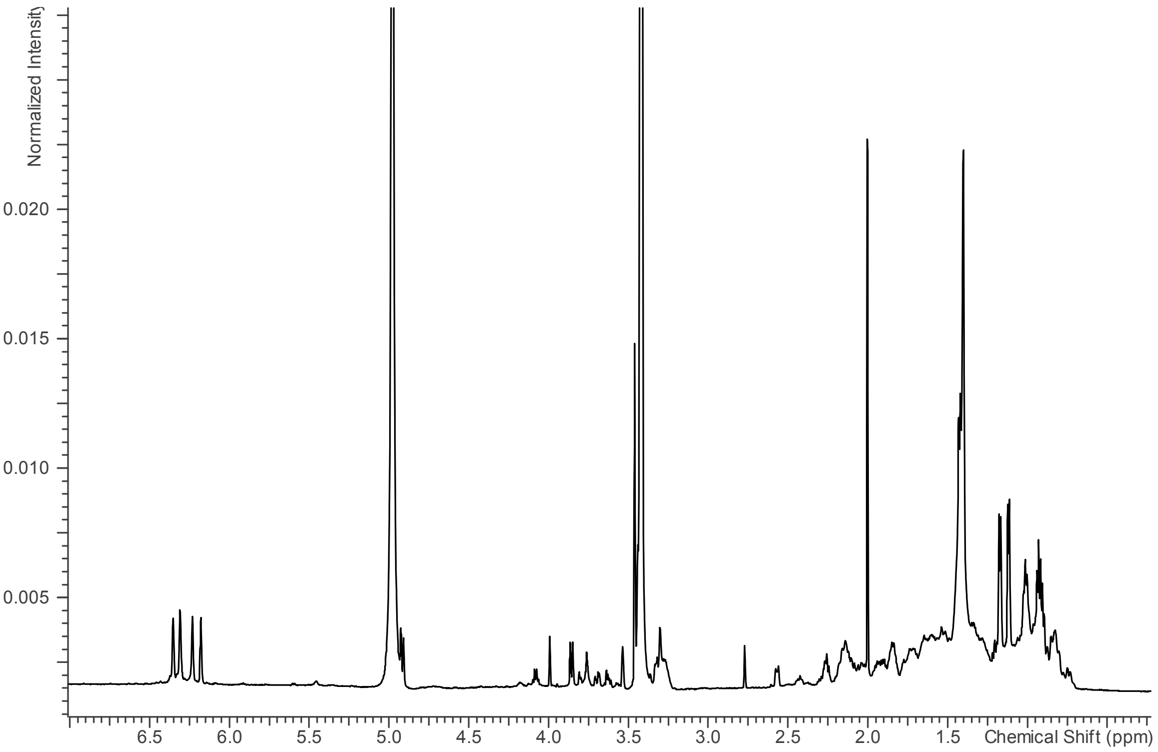


**Figure S30.** $^1$H NMR spectrum (600 MHz, MeOH-$d_4$) of carbamidocyclophane R (**6**).

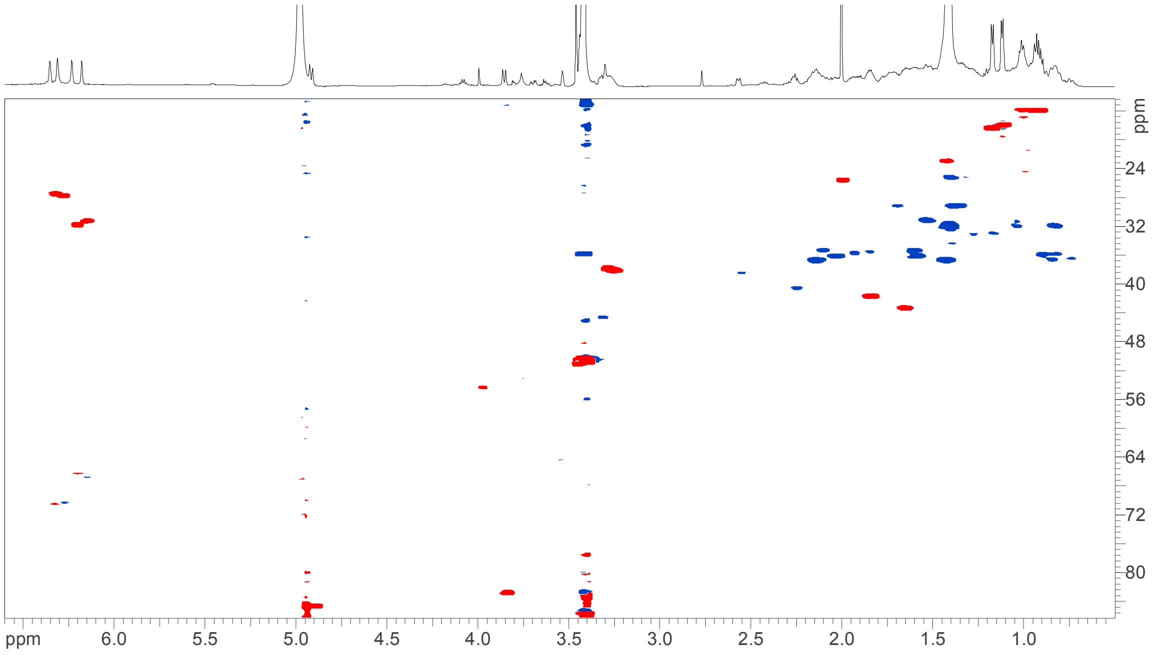


**Figure S31.** HMQC-DEPT spectrum (600 MHz, MeOH-*d*4) of carbamidocyclophane R (**6**). Red signals are attributed to CH or $CH_3$ groups (positively phased) and blue signals to $CH_2$ groups (negatively phased).

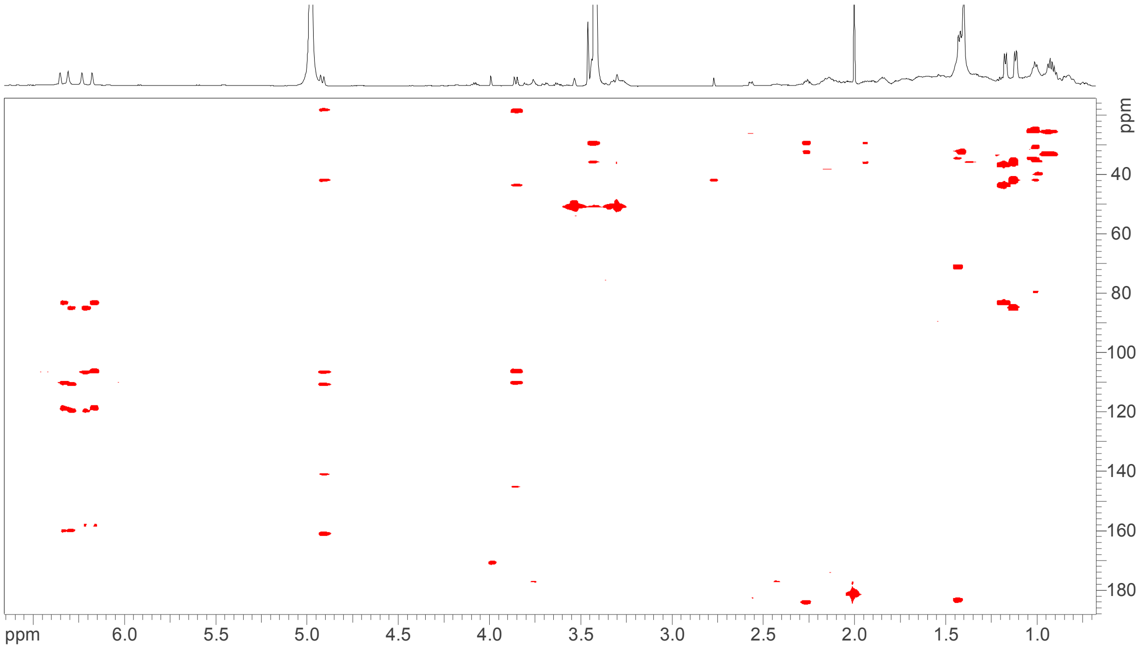


**Figure S32.** HMBC spectrum (600 MHz, MeOH-*d*4) of carbamidocyclophane R (**6**).

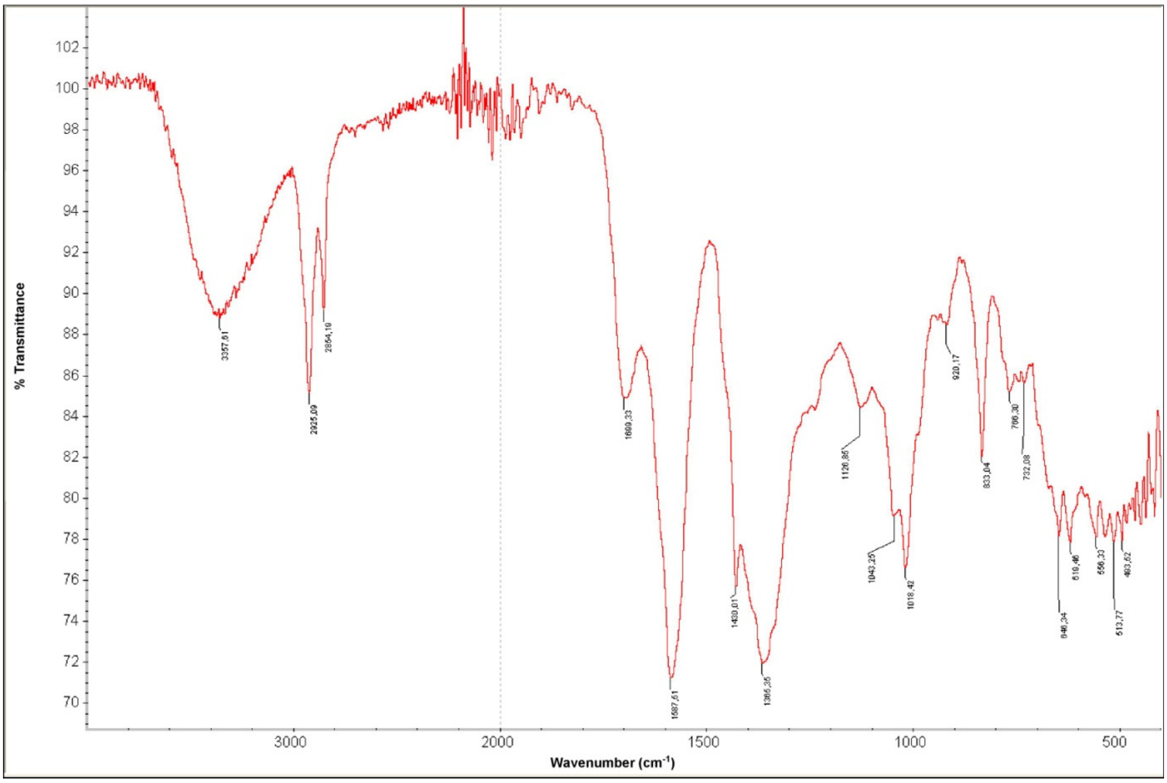


**Figure S33.** ATR-IR (film) spectrum of carbamidocyclophane R (**6**).

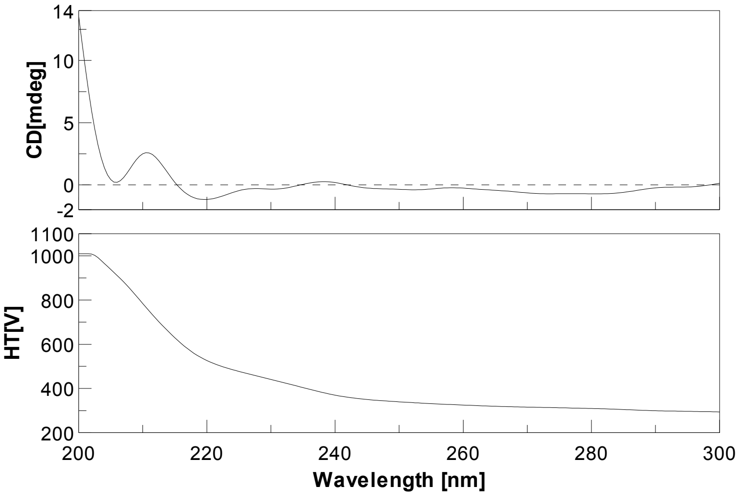


**Figure S34.** ECD spectrum of carbamidocyclophane R (**6**).

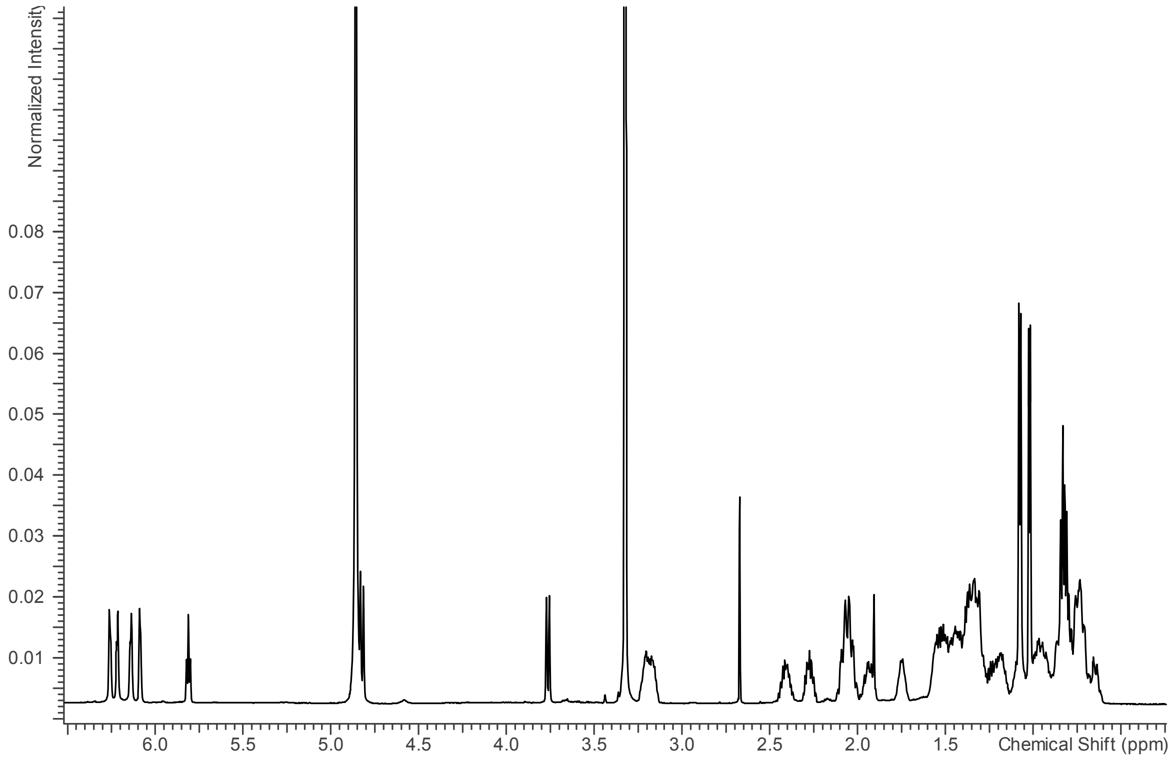


**Figure S35.** $^{1}$H NMR spectrum (600 MHz, MeOH-$d_4$) of carbamidocyclophane S (**7**).

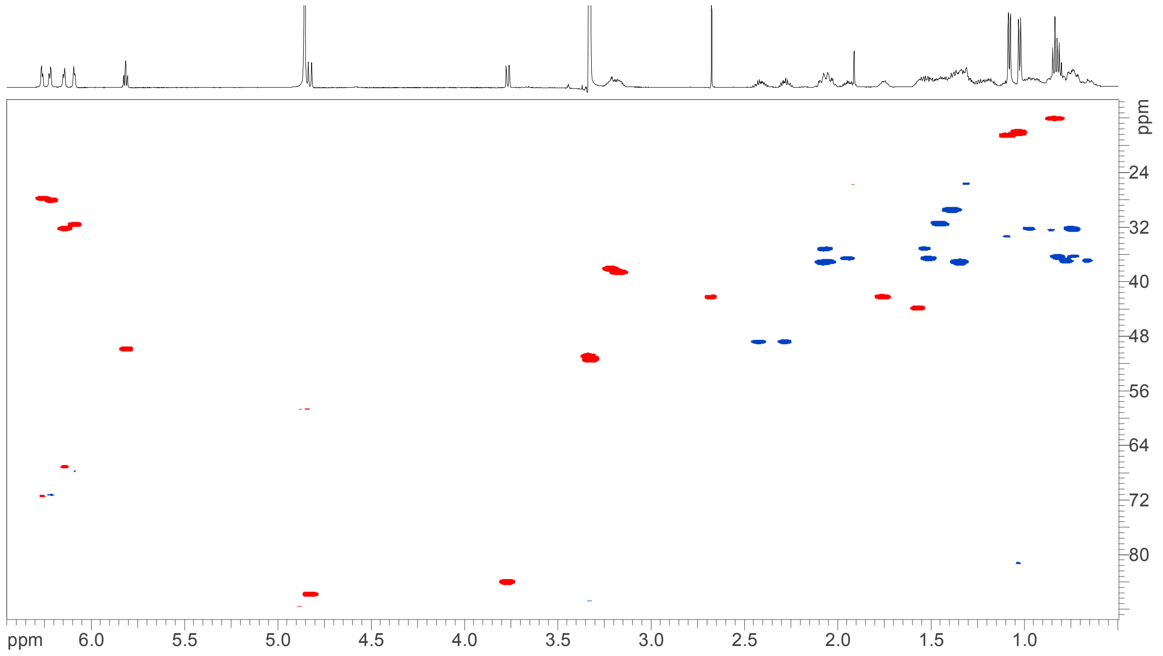


**Figure S36.** HMQC-DEPT spectrum (600 MHz, MeOH-$d_4$) of carbamidocyclophane S (**7**). Red signals are attributed to CH or $CH_3$ groups (positively phased) and blue signals to $CH_2$ groups (negatively phased).

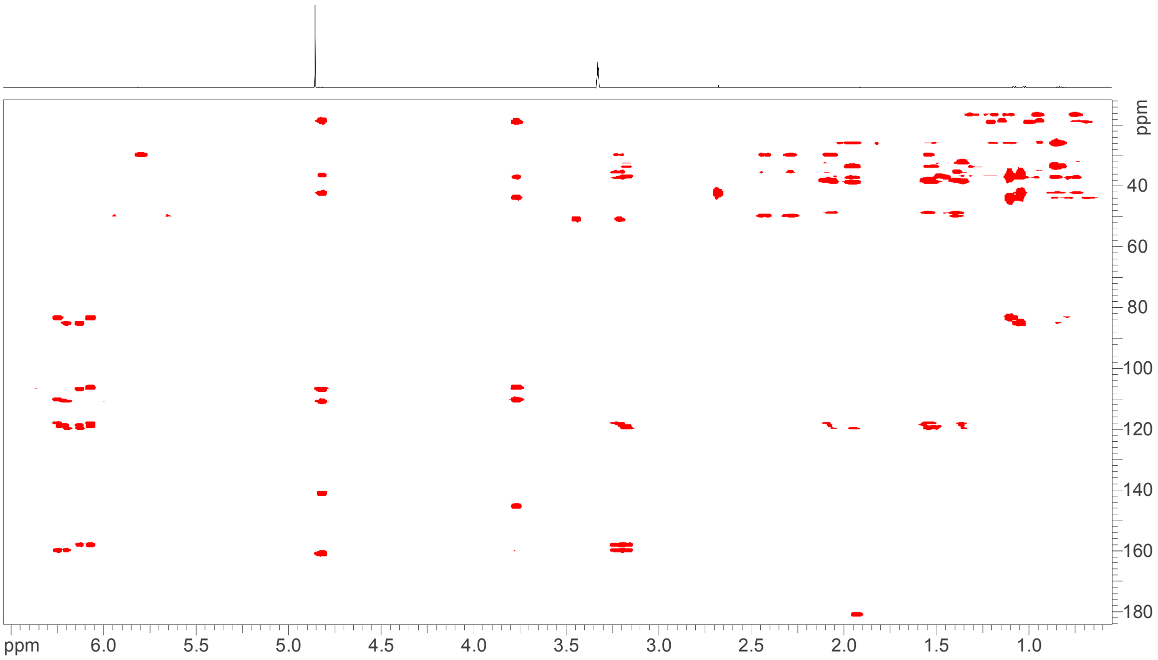


**Figure S37.** HMBC spectrum (600 MHz, MeOH-*d*4) of carbamidocyclophane S (**7**).

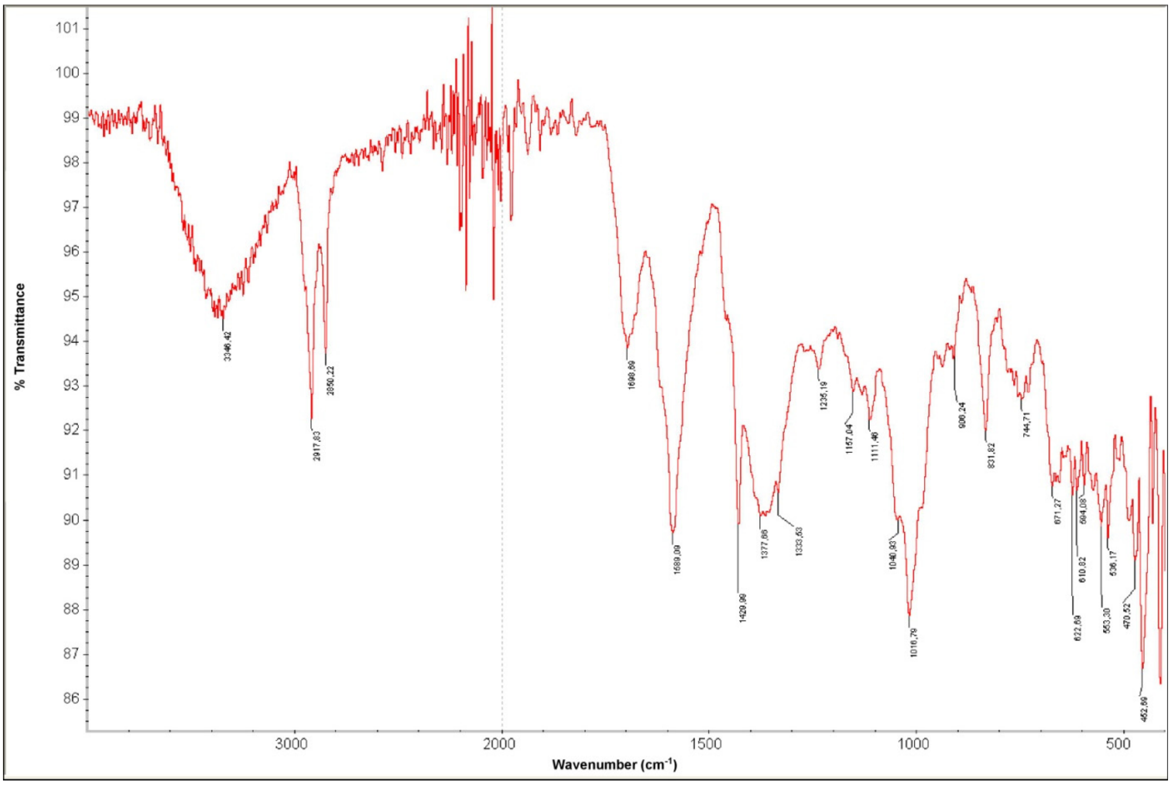


**Figure S38.** ATR-IR (film) spectrum of carbamidocyclophane S (**7**).

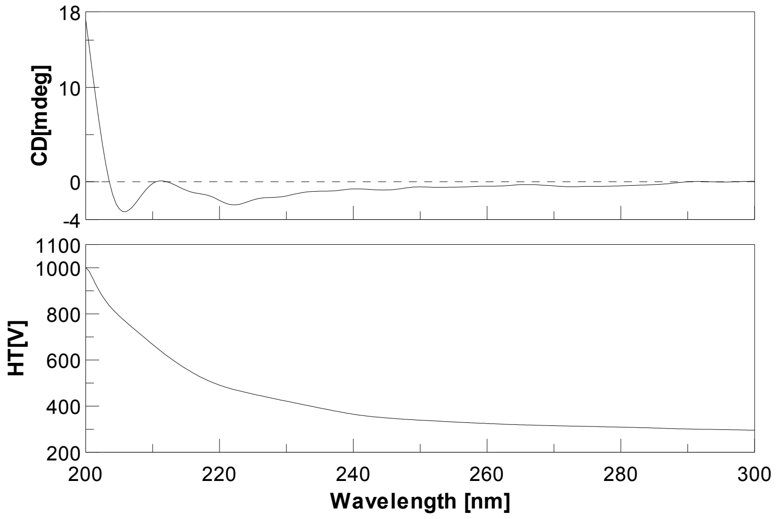


**Figure S39.** ECD spectrum of carbamidocyclophane S (**7**).

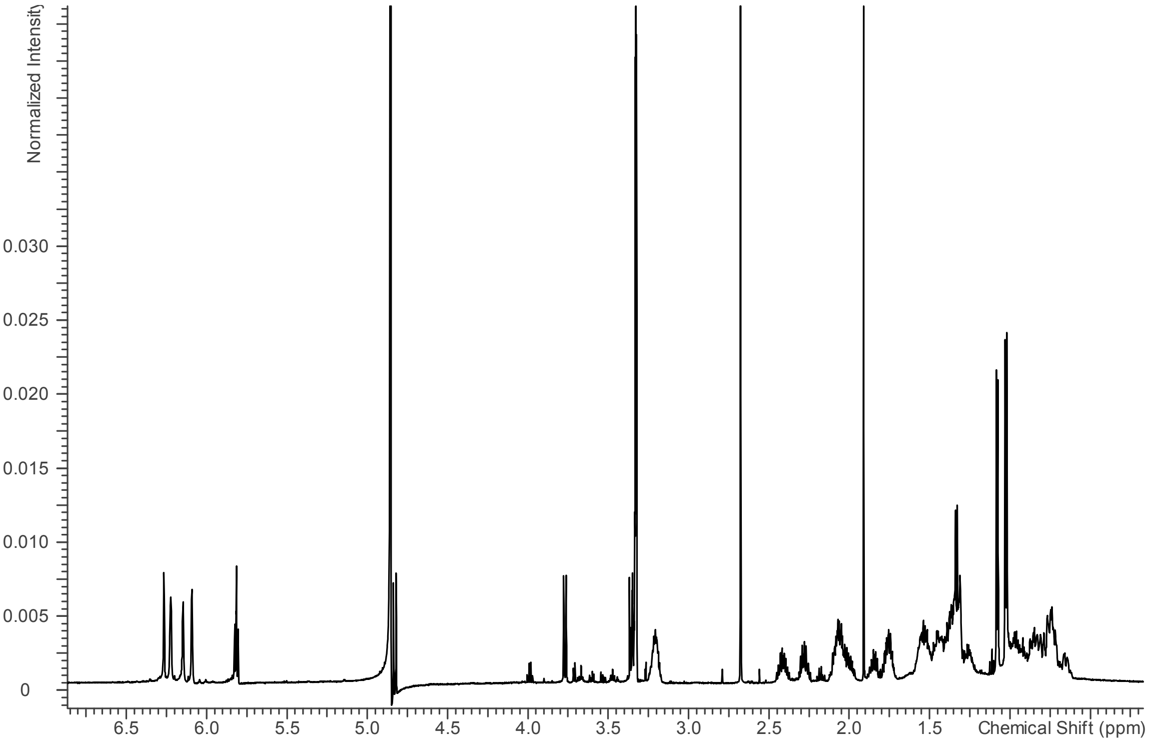


**Figure S40.** $^{1}$H NMR spectrum (600 MHz, MeOH-$d_4$) of carbamidocyclophane T (**8**).

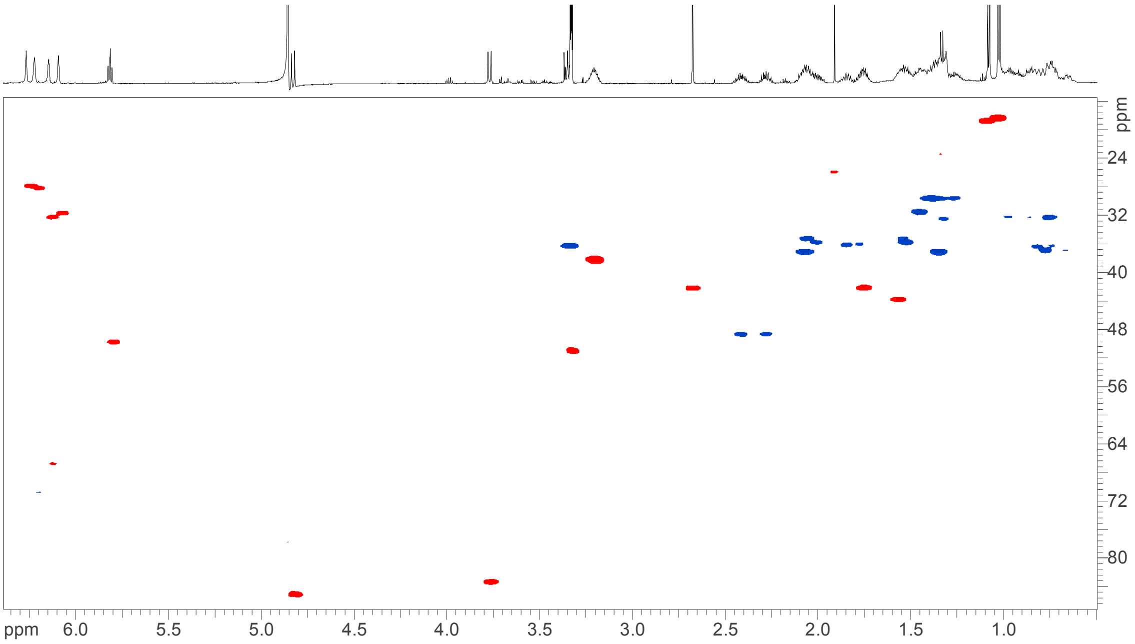


**Figure S41.** HMQC-DEPT spectrum (600 MHz, MeOH-*d*4) of carbamidocyclophane T (**8**). Red signals are attributed to CH or $CH_3$ groups (positively phased) and blue signals to $CH_2$ groups (negatively phased).

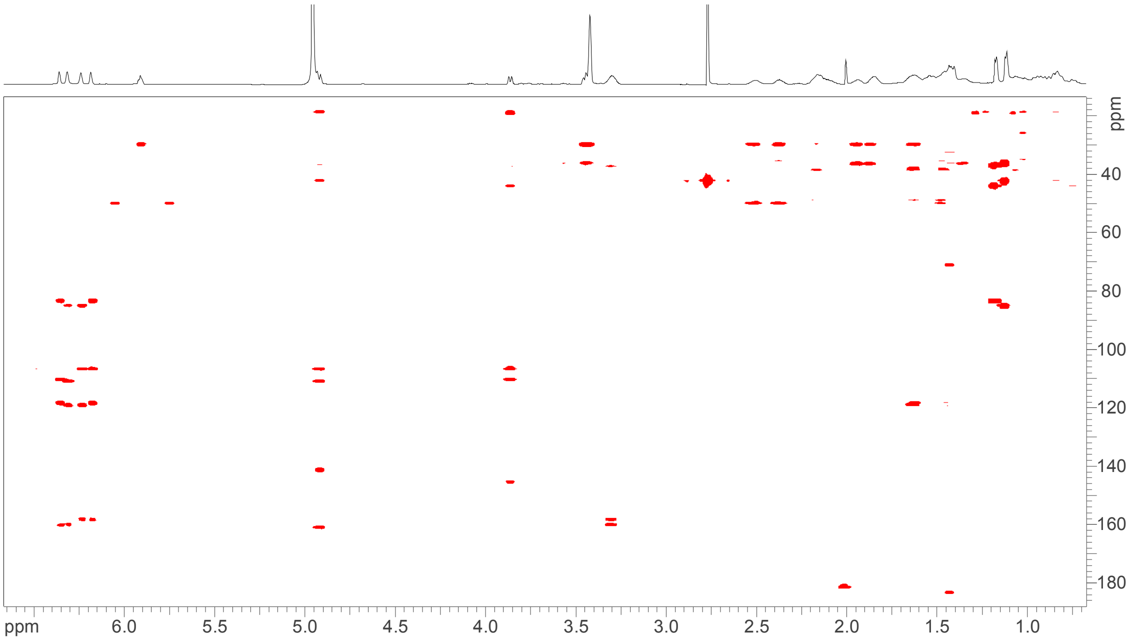


**Figure S42.** HMBC spectrum (600 MHz, MeOH-*d*4) of carbamidocyclophane T (**8**).

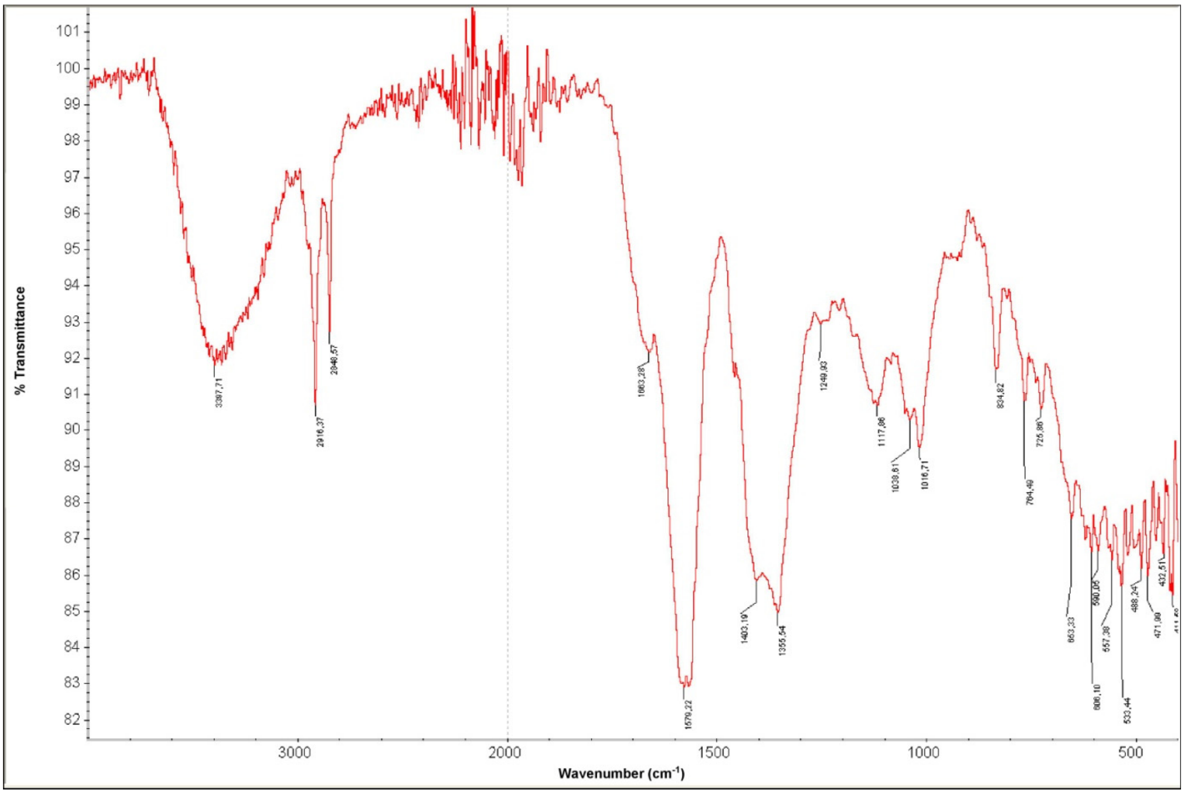


**Figure S43.** ATR-IR (film) spectrum of carbamidocyclophane T (**8**).

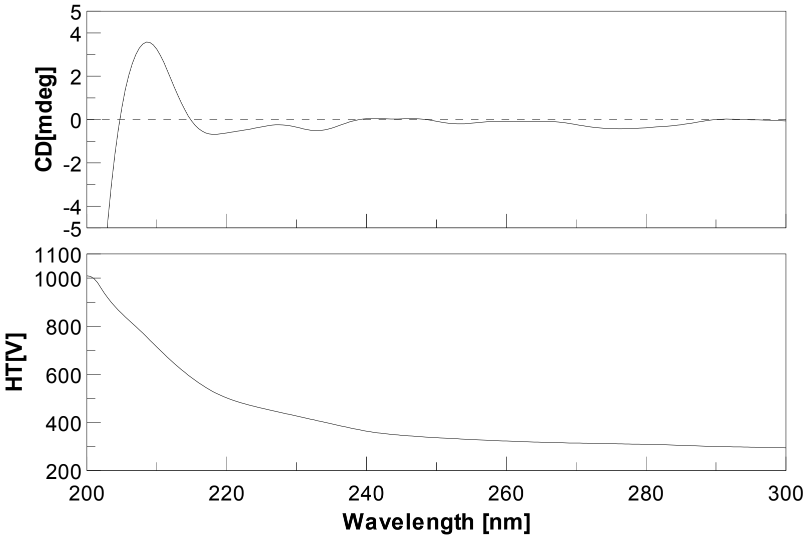


**Figure S44.** ECD spectrum of carbamidocyclophane T (**8**).

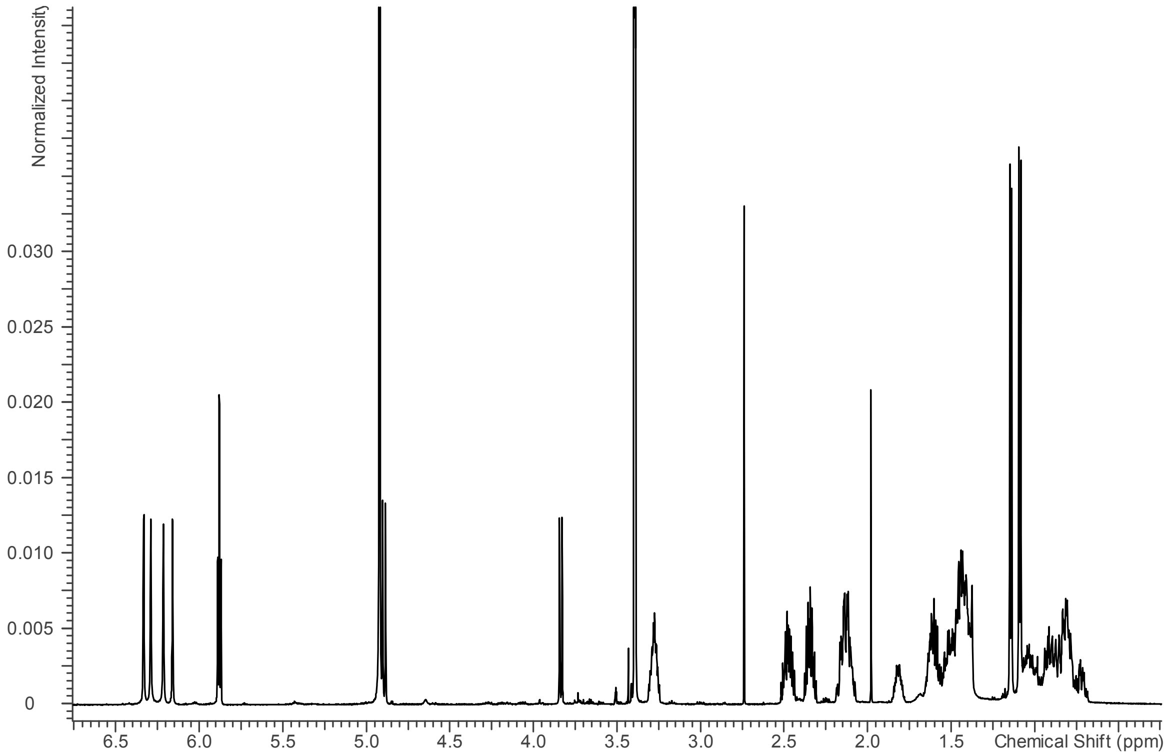


**Figure S45.** $^{1}H$ NMR spectrum (600 MHz, MeOH-$d_4$) of carbamidocyclophane U (**9**).

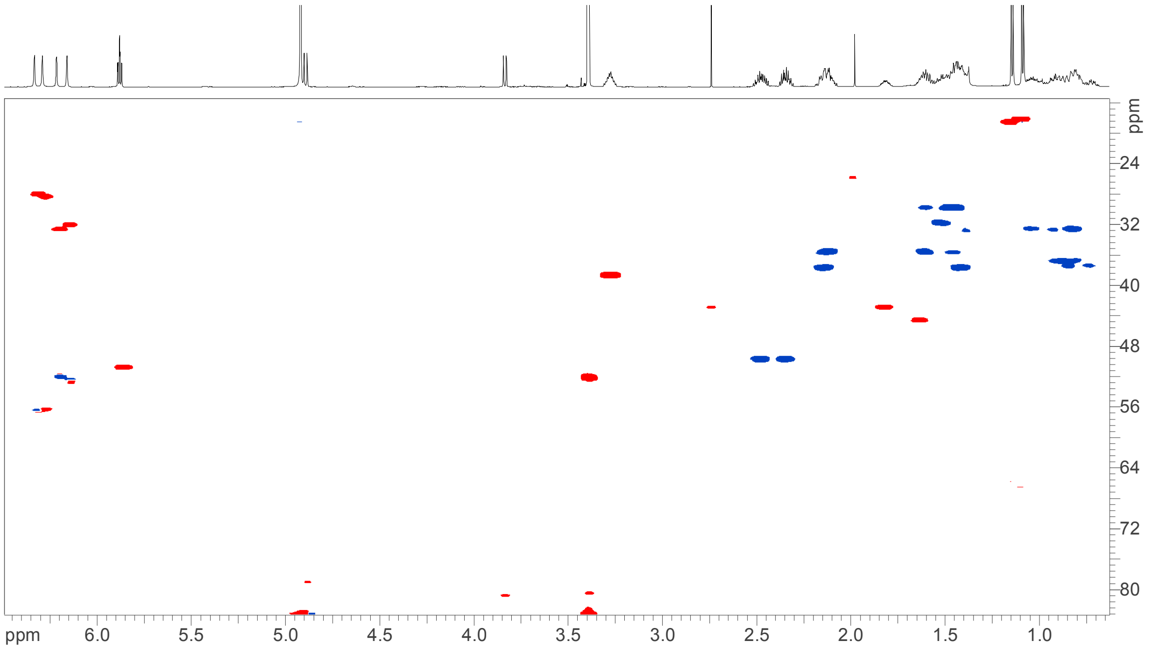


**Figure S46.** HMQC-DEPT spectrum (600 MHz, MeOH-$d_4$) of carbamidocyclophane U (**9**). Red signals are attributed to CH or $CH_3$ groups (positively phased) and blue signals to $CH_2$ groups (negatively phased).

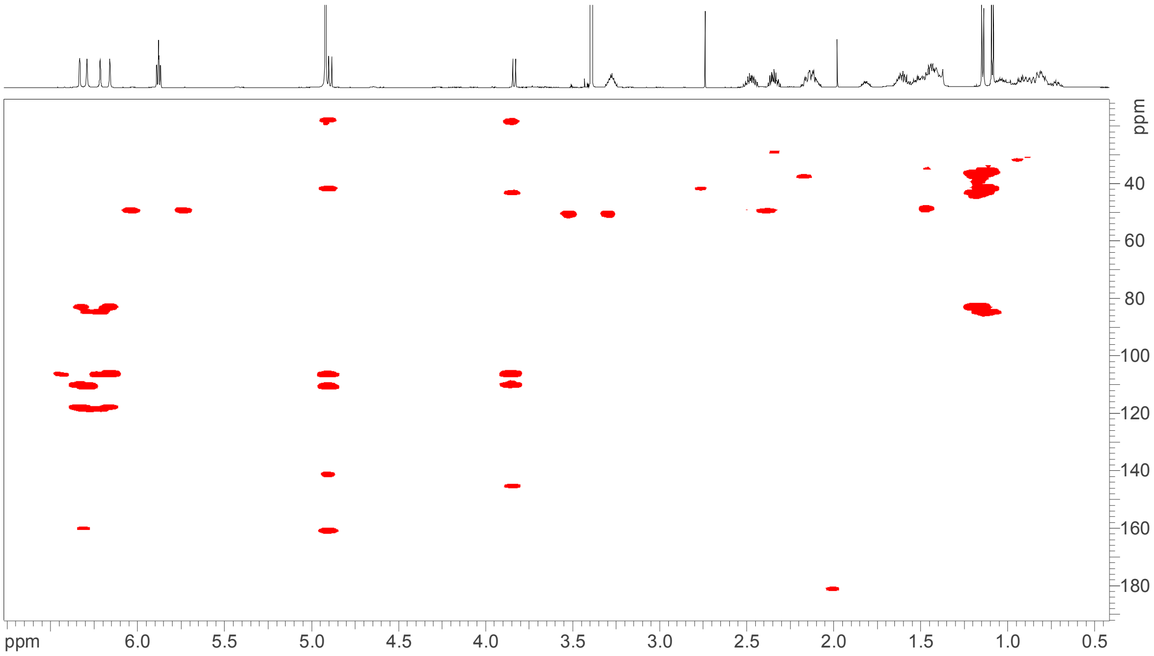


**Figure S47.** HMBC spectrum (600 MHz, MeOH-*d*4) of carbamidocyclophane U (**9**).

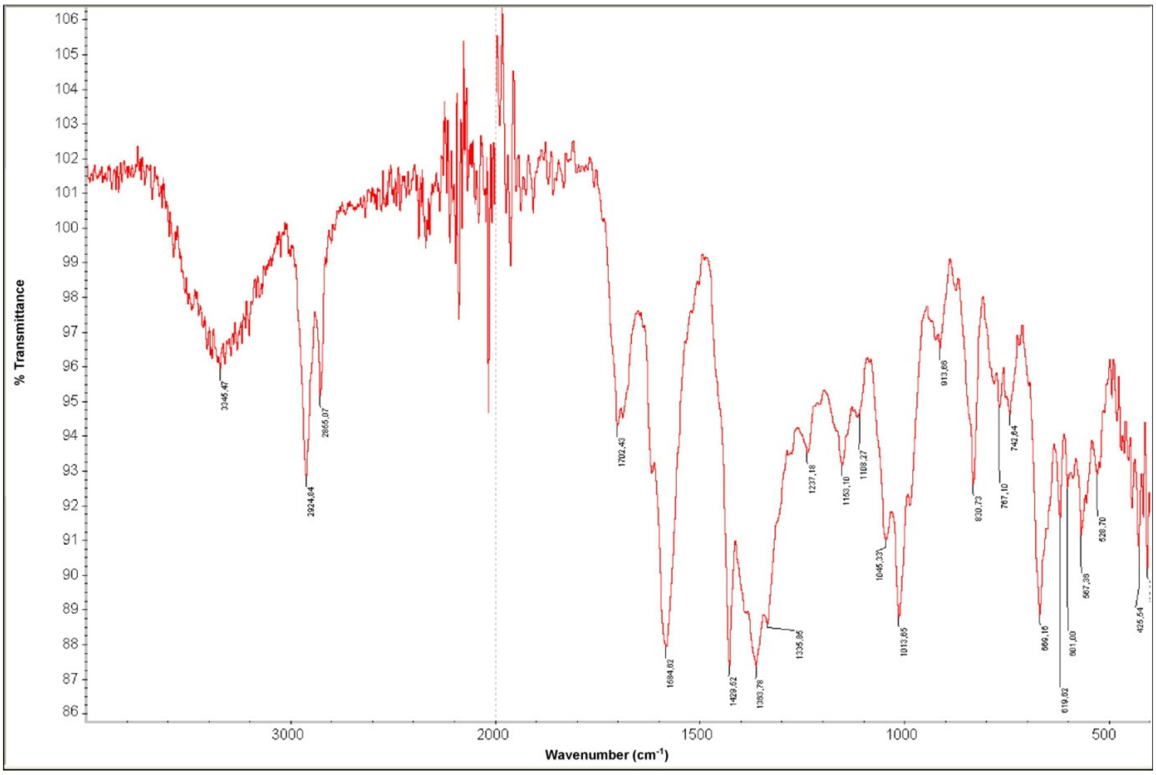


**Figure S48.** ATR-IR (film) spectrum of carbamidocyclophane U (**9**).

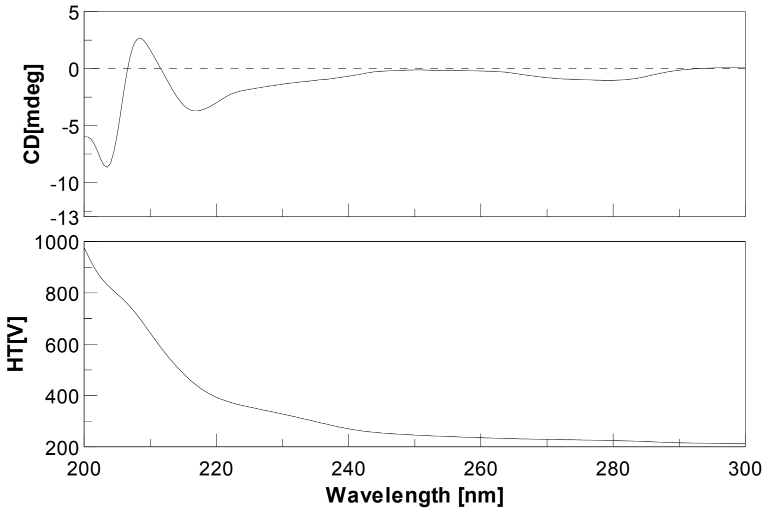


**Figure S49.** ECD spectrum of carbamidocyclophane U (**9**).

## References

1. Falch, B.S.; König, G.M.; Wright, A.D.; Sticher, O.; Angerhofer, C.K.; Pezzuto, J.M.; Bachmann, H. Biological activities of cyanobacteria: evaluation of extracts and pure compounds. *Planta Med.* **1995**, *61*, 321–328.
2. Hughes, E.O.; Gorham, P.R.; Zehnder, A. Toxicity of a unialgal culture of *Microcystis aeruginosa*. *Can. J. Microbiol.* **1958**, *4*, 225–236.
3. Meffert, M.E. Cultivation and growth of two planktonic *Oscillatoria* spec. *Mitt. Int. Ver. Theor. Angew. Limnol.* **1971**, *19*, 189–205.
4. Pretsch, A.; Nagl, M.; Schwendinger, K.; Kreiseder, B.; Wiederstein, M.; Pretsch, D.; Genov, M.; Hollaus, R.; Zinssmeister, D.; Debbab, A.; *et al*. Antimicrobial and anti-inflammatory activities of endophytic fungi *Talaromyces wortmannii* extracts against acne-inducing bacteria. *PLoS ONE* **2014**, *9*, e97929.
5. Preisitsch, M.; Harmrolfs, K.; Pham, H.T.L.; Heiden, S.E.; Füssel, A.; Wiesner, C.; Pretsch, A.; Swiatecka-Hagenbruch, M.; Niedermeyer, T.H.J.; Müller, R., et al. Anti-MRSA-acting carbamidocyclophanes H-L from the Vietnamese cyanobacterium Nostoc sp. CAVN2. *J. Antibiot*. **2015**, *68*, 165–177.
6. Preisitsch, M.; Niedermeyer, T.H.J.; Heiden, S.E.; Neidhardt, I.; Kumpfmüller, J.; Wurster, M.; Harmrolfs, K.; Wiesner, C.; Enke, H.; Müller, R., *et al.* Cylindrofridins A–C, linear cylindrocyclophane-related alkylresorcinols from the cyanobacterium Cylindrospermum stagnale. *J. Nat. Prod.* **2015**, doi:10.1021/acs.jnatprod.5b00768.
